# Sustainable approach for synthesis of new coumarin-linked Schiff bases in DABCO-based ionic liquid and their identification as aldose reductase inhibitors

**DOI:** 10.1038/s41598-025-97949-6

**Published:** 2025-04-24

**Authors:** Marium Ishtiaq, Naved Iqbal, Aqeel Imran, Mariya al-Rashida, Sobia Rana, Maria Aqeel Khan, Jamshed Iqbal, Abdul Hameed

**Affiliations:** 1 Third World Center for Science and Technology, International Center for Chemical and Biological Sciences (ICCBS), University of Karachi, Karachi, 75270 Pakistan; 2 H.E.J. Research Institute of Chemistry, International Center for Chemical and Biological Sciences (ICCBS), University of Karachi, Karachi, 75270 Pakistan; 3 Center for Advanced Drug Research, COMSATS University Islamabad, Abbottabad Campus, Abbottabad, 22060 Pakistan; 4 Department of Chemistry, COMSATS University Islamabad, Abbottabad Campus, Abbottabad, 22060 Pakistan; 5 Department of Chemistry, University of Sahiwal, Sahiwal, 57000 Pakistan; 6 Department of Chemistry, Forman Christian College (A Chartered University), Lahore, Pakistan; 7 Molecular Biology and Human Genetics Laboratory, Dr. Panjwani Center for Molecular Medicine and Drug Research (PCMD), International Center for Chemical and Biological Sciences (ICCBS), University of Karachi, Karachi, 75270 Pakistan

**Keywords:** Ionic liquids, 1,4-Diazabicyclo[2.2.2]octane, Catalysis, Aldose reductase Inhibition, Diabetes, Green chemistry, Medicinal chemistry, Drug discovery, Chemistry

## Abstract

Aldehyde reductase (ALR1) is the enzyme that speeds up the reduction of many types of aldehydes into sorbitol and D-glucose. The essential enzyme of the polyol pathway, aldose reductase (ALR2), is responsible for the development of chronic complications associated with diabetes when activated under hyperglycemic conditions. Since it is a crucial mediator for the oxidative and inflammatory signaling pathways, ALR is thought to be a target for various diseases. Many medicines are available for the treatment of ALR-associated issues but due to their long term side-effects they are not effectively used. Coumarin is a naturally occurring compound, and its derivatives are widely used in the treatment of many ailments. Therefore, in the pursuit to find potential alternate candidates as drug leads, we have prepared new coumarin-based Schiff base analogues using DABCO-*C*_*7*_-F ionic liquid and compared with conventional method. The sustainable approach making use of DABCO-*C*_*7*_-F ionic liquid, not only made the synthesis easier but also it is cost- and time-effective. The synthesized analogues were further examined for their potentials against ALR2 (IC_50_ = 1.61 to 11.20 *µ*M) as well as checked selectivity *via* screening against ALR1 enzyme. Moreover, the molecular docking study was performed to elucidate the binding interactions of active compounds. The results showed that the synthesized compounds may have the potential to be further studied as new and selective anti-diabetic agents.

**Supplementary Information:**

The online version contains supplementary material available at 10.1038/s41598-025-97949-6.

## Introduction

**Fig. 1:**
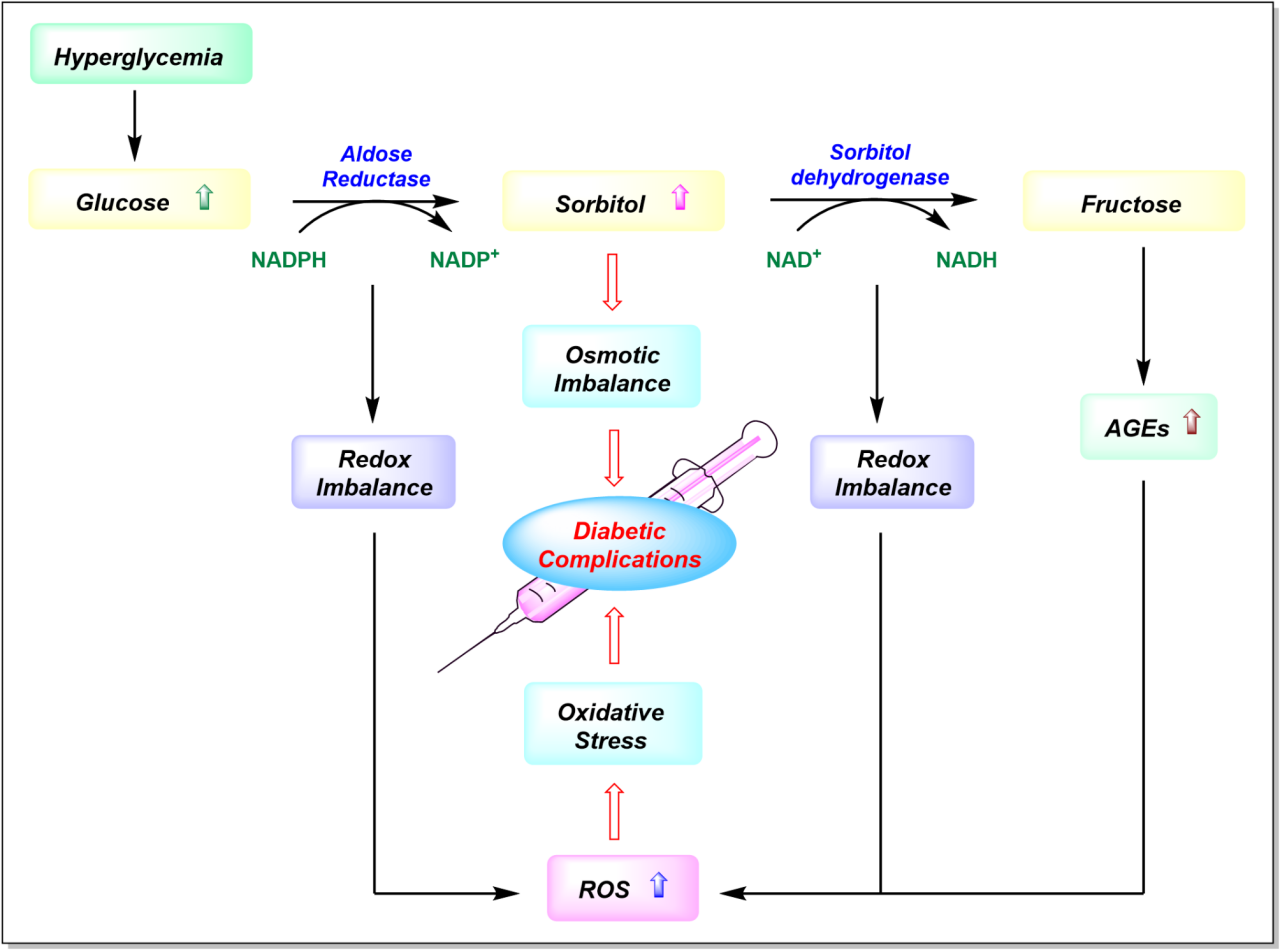
Polyol pathway of glucose metabolism and pathogenesis of diabetic complications.

Globally, almost 537 million adults with the age of 20–79 years are living with diabetes. The current statistics of diabetes mellitus (DM) incidence is predicted to rise 643 million (11.3%) by 2030, and to 783 million (12.2%) by 2045. This chronic condition may lead to fatality in later coming years. To overcome this issue, there are several targets working under different pathways to treat type II DM, such as aldose reductase, DPP-IV, *α*-amylase, anti-glycation and *α*-glucosidase enzyme inhibitors^1,2^, *etc*. Amid them, aldose reductase inhibitors are new attractive drug target, and is being focused since last few decades in the clinical management of diabetic complications. Aldose reductase is a cytoplasmic reductase enzyme, responsible for eliminating toxic carbonyl compounds from the body, generated *via* peroxidation of lipid biomolecules. It is involved in the conversion of the glucose into sorbitol through polyol pathway. In normal condition, the insulin secretion from the pancreas′ *β-*cells converts the glucose into glycogen, and eventually store it in the form of starch^3^. In case of impaired activity of *β-*cells (most probably in case of type-2 diabetes mellitus), the glucose level increases tremendously and activates polyol pathway, which is a minor route for the conversion of glucose into fructose in two steps. In the first step, the glucose is converted into sorbitol by the action of aldose reductase (AR) utilizing NADPH as coenzyme; in the second step, sorbitol is converted in fructose in the presence of sorbitol dehydrogenase (SDH) enzyme using NAD^+^ as coenzyme (Fig. 1), under euglycemic condition; this is a minor glucose metabolism pathway^4^.

Under hyperglycemic condition, the polyol pathway is more active^5^. The conversion of glucose to sorbitol leads to the accumulation of sorbitol that result in osmotic stress, cellular damage, specifically to that of lenses^6^. Moreover, this step is NADPH-dependent which causes decrease in the availability of NADPH for other NADPH-dependent enzymes such as glutathione reductase^3^; thus, resulting in the imparity between the antioxidant defense system of the cell and the intracellular reactive oxygen species (ROS). Consequently, depletion of NAD^+^ due to the SDH-catalyzed transformation of sorbitol to fructose give rise to pseudohypoxia, worsening redox imbalance, and resulting in further metabolic and signaling variations. Elevated level of fructose is also one of main causes for the formation of Advanced glycation products (AGEs), which changes the functions of protein leading to the productions of intercellular ROS^3,7^.

Besides, aldose reductase (AR) is not only a part of the aldo-keto reductase family that is involved in the pathogenesis of severe problems arises with type-2 diabetes mellitus (T2DM). However, it is also involved in other serious diseases like asthma, sepsis, cancer, cardiovascular diseases and rheumatoid arthritis. It acts as a key mediator for oxidative and inflammatory signaling pathway, and this is the reason that AR considered as a target for multiple diseases^7,8^, particularly for controlling diabetes.

AR inhibitors are mainly categorized into two main classes: carboxylic acid derivatives and non-carboxylic acid derivatives (thiazolidinedones, 5-phenyl pyrroles, spirofluorene-imidazolidines, sucinimides, *etc*.) that presented IC_50_ values ranging between 0.015 and 1 *µ*M^9,10^. Many marketed drugs are available as inhibitors of aldose reductase enzyme that belongs to these classes; among which imirestat (spirofluorene-imidazolidine analogue), sorbinil (spirofluorene-imidazolidine analogue), alrestatin (benzo[*de*]isoquinolin-2(3*H*)-yl)acetic acid analogue), epalrestat (thiazole-linked carboxylic acid derivative) and minalrestat (spiro[isoquinoline-pyrrolidine derivative), *etc*. are well-known. They are used to selectively suppress the secondary complications caused by diabetes, like in those tissues where the absorption of glucose is not insulin dependent (*i.e*., the lens, neural tissues and glomeruli). Though AR therapy is one of the interesting methods to suppress diabetic complications; however, most of the drugs have been eliminated because of the reported antagonistic effects of the drugs, which includes toxic epidermal necrolysis, diarrhea, fever, skin rashes, hepatic necrosis, splenomegaly and Stevens-Johnson syndrome^11^. They showed promising results in *in-vitro *and *in-vivo* studies but when subjected to clinical trial with humans, they offered aforementioned side effects; such as sorbinil was discontinued due to hypersensitivity reactions, tolrestat showed liver toxicity, *etc*. To date, epalrestat is successfully marketed in few countries for the treatment of neuropathy associate with diabetes^10^. Thus, this opens a window to explore new, selective, and safe classes of ALR inhibitors.

**Fig. 2:**
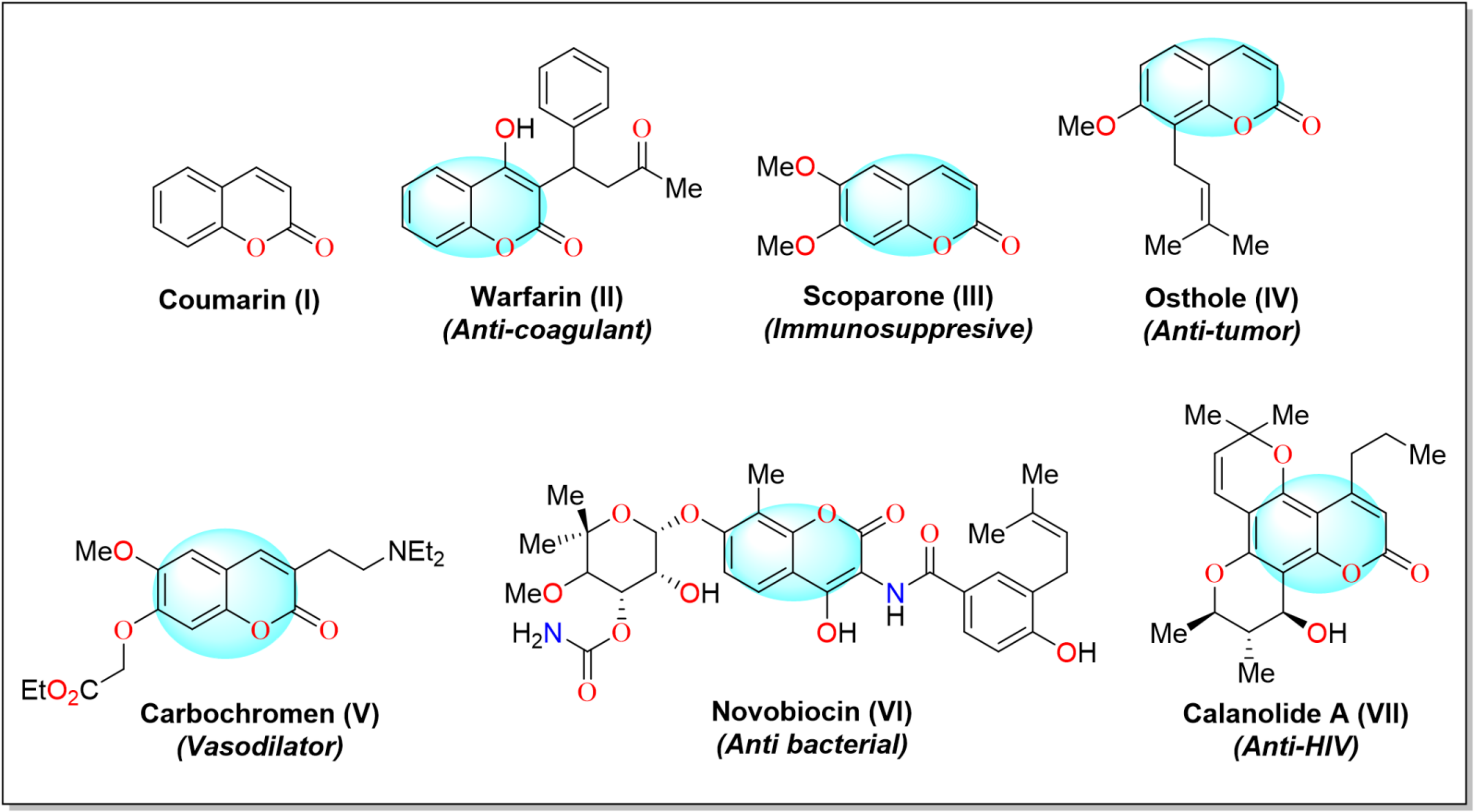
Coumarin (**I**) and its bioactive analogues (**II-VII**).

Coumarin (**I**) is a large class of organic aromatic compounds distributed extensively in nature. Structurally coumarin is composed of 1-benzopyran-2-one motif, which is consisted of fused pyrone and benzene with the pyrone carbonyl at 2-position. According to IUPAC nomenclature, it is named as 2*H*-chromen-2-one^12^. Coumarin and its derivatives (**II-VII**) are widely studied compounds due to their broad biological and pharmacological activities. Among them, key potencies include the anti-proliferative, anti-cancer, anti-bacterial, anti-neurodegenerative, anti-coagulant, anti-HIV, anti-viral, and anti-inflammatory agents, as illustrated in Fig. 2^12–14^.

**Fig. 3:**
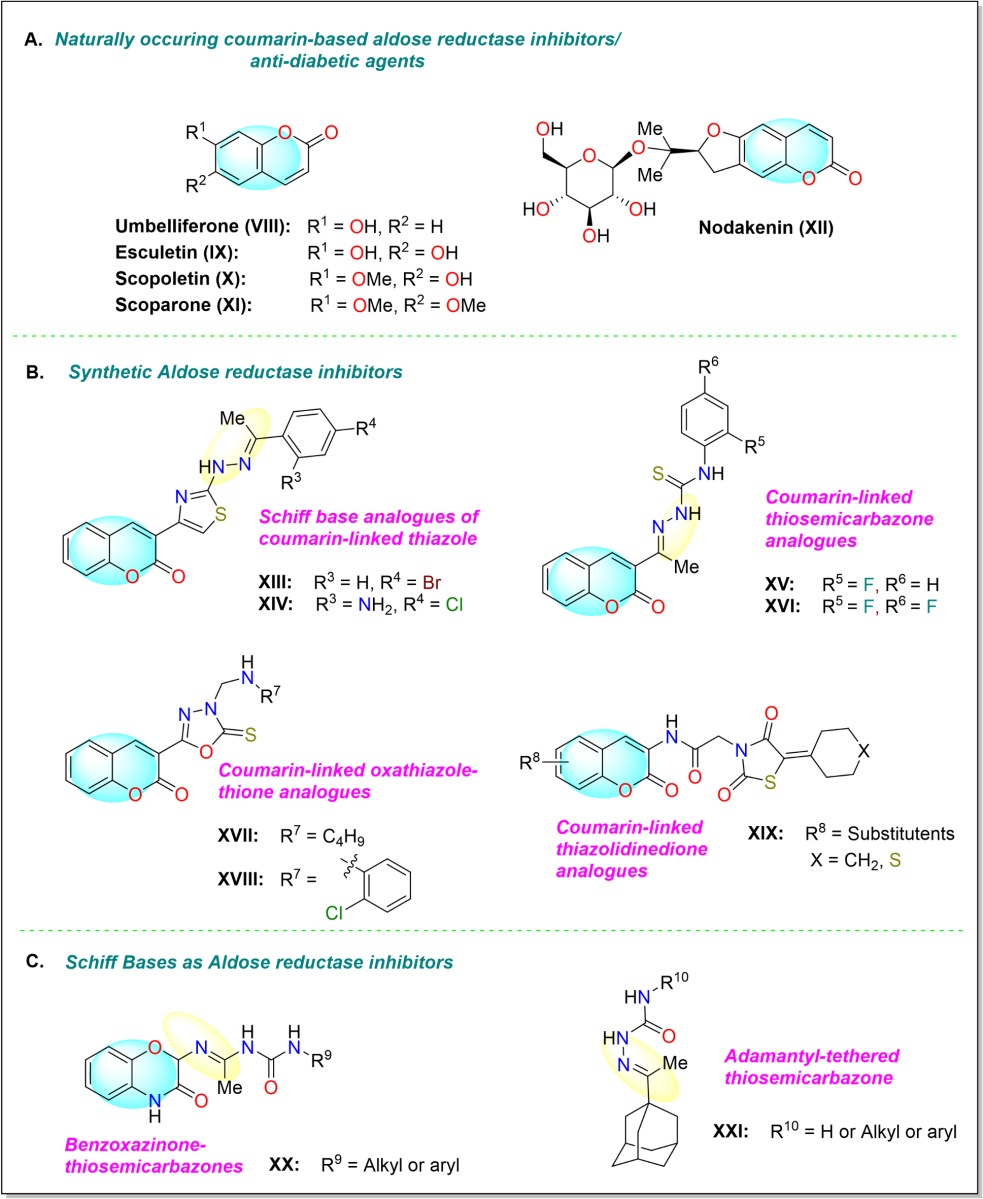
Coumarin derivatives as aldose reductase inhibitors or anti-diabetic agents.

Besides, coumarin derivatives isolated from different natural sources have shown promising activity against diabetes. Esculetin (**IX**)^15^, nodakenin (**XII**)^16^, scoparone (**XI**), umbelliferone (**VIII**) and scopoletin (**X**)^17^ are such examples of naturally-occuring coumarins (Fig. 3). In addition, studies revealed that coumarin scaffold in combination with other moieties such as thiazole, oxadiazole derivatives^18^, thiosemicarbazone^19^, and thiazolidinedione^20^ have significantly enhancing activity against ALR enzyme, as shown in Fig. 3. Ibrar and co-workers reported coumarin-oxadiazolethione analogue (**XVII** and **XVIII**) as new class of ALR inhibitor, which showed IC_50_ values ranging between 0.31 and 57.5 *µ*M. The same group reported another library of coumarin-thiazole analogues (**XIII** and **XIV**) with IC_50_ values ranging between 0.11 and 6.35 *µ*M^18^. A series of coumarin-tethered thiosemicarbazone derivatives (**XV** and **XVI**) exhibited promising aldose reductase inhibition (2–28 *µ*M). Among the series, 2-fluorophenyl substituted analogue (**XV**) was the most inhibitor of ALR2 with IC_50 _value of 2.07 *µ*M. It also exhibited high selectivity relative to ALR1 enzyme^19^. Pasala group reported coumarin-thiazolidinedione analogues (**XIX**) as new and interesting class of ALR inhibitors. SAR studies suggested that compound with cyclopentyl ring on the 1,3-thiazolidine-2,4-dione ring was found active analogue with IC_50_ value of 3.89 ± 0.55 *µ*M^20^. It is noteworthy that compounds **XIII**-**XVI** comprised of Schiff base moiety in addition to heterocyclic scaffolds; moreover, Schiff base analogues have also shown tremendous activity against aldose reductase enzyme^21,22^. In particular, thiosemicarbazone derivatives of benzoxazinone (**XX**) were synthesized as potential inhibitor of aldose reductase enzyme with IC_50_ values ranging between 0.19 and 10.6 *µ*M against sorbinil inhibitor (IC_50_ = 3.14 ± 0.02 *µ*M), and can acts as antidiabetic leads. Most active analogue was found to be dual inhibitor of ALR2 and ALR1 enzymes with IC_50_ values of 0.19 ± 0.03 and 1.72 ± 0.02 *µ*M, respectively^22^. Adamantyl-tethered thiosemicarbazone derivatives (**XXI**) were also found to show significant aldose reductase (ALR2) inhibition with IC_50_ values ranging between 1.37 and 38.4 *µ*M. Amid them, most active compound in the series was found dual inhibitor of both enzymes with IC_50_ values of 1.37 ± 0.92 (ALR2) and 7.04 ± 2.23 *µ*M (ALR1), respectively^23^. On the basis of above mentioned studies, our group has designed new coumarin-based Schiff base hybrids and planned to evaluate the obtained products for their biologically active potential against ALR1 and ALR2 enzymes.

The use of ionic liquids (ILs) in terms of green chemistry has opened new horizons in the research field. They are getting attention due to their robust and environmental friendly effects including cost efficiency and recyclability^24^. Initially, the successful transformations for the synthesis of Aldol-type reactions were the primary goals after the discovery of ILs. Later than, successful Michael addition reactions carried out in the presence of ILs are also reported^25^. The gradual yet eccentric performance of ILs has made the foundation in organic synthesis to perform the organic transformations in very facile way. The reactions performed earlier using classical strategies have now been replaced by the use of ILs. Recently, the facile synthesis of heterocyclic analogues using new hydrogen bond-rich glucose-based ionic liquids describes the promising use of sustainable approach in the field of organic chemistry^26^. Moreover, another report showed promising utilization of slightly different D-glucose DABCO-based ionic liquid by using the microwave-assisted organic transformation, which has proven to be yet another excellent sustainable approach for the synthesis of different compounds^27^. As natural sugars are widely abundant in good quantities and are inexpensive, another fascinating approach is reported where sugar-based ionic liquids are used as catalysts to perform different synthetic methodologies, chiral recognition and organocatalysis^28,29^. In the current study, we aim to prepare the designed coumarin-Schiff base compounds as ALR inhibitors using ionic liquid in order to promote sustainable synthetic methodologies.

## Methods

### Chemicals and characterization

All the chemicals were commercially purchased from Sigma Aldrich and were used without any purification. The Bruker Vector-22 spectrometer was used to record infrared (IR) spectra. The Bruker spectrometers were used to record ^1^H-NMR at 300, and 400 MHz; ^13^C-NMR spectra on 75 and 125 MHz; and 2D-NMR (HSQC, HMBC, COSY, NOESY and TOCSY) on 600 MHz in DMSO-*d*_*6*_ solvents using trimethylsilane (TMS) as an internal solvent. The Finnigan MAT-321 A, Germany, spectrometer was used to perform mass spectrometry analysis. The BÜCHI M65 instrument was used to get the melting points of all solid compounds. The silica gel 60 aluminum-backed plates 0.063–0.200 mm were used to perform TLC to monitor the reaction and purification. Short-wavelength UV radiation at 254 nm was used for visualization of TLC plates.

### General procedure for the synthesis of DABCO-F ionic liquids (2,3)

An oven-dried round-bottomed flask was charged with DABCO (4.45 mmol) with a mixture of solvents (dichloromethane and toluene) in a ratio of 5:2 mL. 1-Bromopentane or 1-bromoheptane (17.8 mmol) was added to the solution and allowed to reflux at 118–120 °C. After the completion of the reaction as per TLC analysis, the resulting ionic liquid was cooled down at room temperature. It was further washed using cold toluene (10 mL×3) and dried under vacuum to get the concentrated DABCO-Br based ionic salt. The formation of DABCO-Br was confirmed by performing ^1^H-NMR spectroscopy. Next, the DABCO-Br product was further treated with silver fluoride (4.45 mmol) for the counter anionic part change with fluoride to afford corresponding DABCO-F based ionic liquids. The synthesized product was characterized by spectroscopic techniques and compared to the reported data^24^.

### Procedure for the synthesis of coumarin aldehyde (3)

Following the known procedure^30^, to the solution of salicylaldehyde (50 mmol) in methanol (50 mL), triethylamine (50 mmol) was added and stirred at room temperature. After that, methyl acrylate (150 mmol) was added dropwise to the reaction mixture and stirred for 24 h. Precipitates were formed, which were filtered from the reaction mixture and thoroughly washed with methanol to obtain pure product^31^ (**3**).

#### 2′-((2-oxo-2*H*-chromen-3-yl)methoxy)benzaldehyde (3)

White solid; Yield: 74%; m.p.: 188–190 °C (lit: 190 °C); IR (*ν*_max_, cm^−1^): (KBr disc): 3073, 3039, 2919 (H‒CO), 1717 (C = O), 1682 (C=O), 1601 (C=C), 1485 (C=C), 1453 (C=C), 1402, 1288, 1247 (C‒O), 1179 (C‒O), 1082 (C‒O), 1024 (C‒O); ^1^H-NMR (300 MHz, DMSO-*d*_*6*_): *δ*_H_ 10.51 (s, 1H, C*H*O-7′), 8.27 (s, 1H, H-4), 7.82 (d, 1H, *J*_3′,4′_ = 7.8 Hz, H-3′), 7.74 (dd, 1H, *J*_5,6_ = 7.5 Hz, *J*_5,7_ = 1.5 Hz, H-5), 7.70–7.60 (m, 2H, H-7, H-5′), 7.45 (d, 1H, *J*_8,7_ = 8.1 Hz, H-8), 7.40–7.34 (m, 2H, H-6, H-6′), 7.13 (t, 1H, *J*_4′,(3′,5′)_ = 7.5 Hz, H-4′), 5.14 (s, 2H, C*H*_2_-9); EI-MS *m/z* (%): 280 (M^+^, 3), 159 (100), 131 (38), 115 (68), 103 (21), 77 (54), 51 (26).

### General procedure for the synthesis of coumarin-linked Schiff-base hybrids (5–22)

**Method-A**: To the solution of coumarin aldehyde (1 mmol) in ethanol (EtOH), substituted phenylhydrazine hydrochlorides (1.5 mmol) were added followed by 10 mol% *p*-TsOH as a catalyst. The resulting reaction mixture was allowed to reflux at 70–80 ^º^C. The progress of the reaction was observed by TLC using a solvent system of EtOAc and petroleum ether (20%). Upon completion, the reaction mixture was cooled at room temperature for precipitation of the product. The obtained precipitates were then filtered and washed thoroughly with cold ethanol to impurity; affording corresponding coumarin-linked Schiff-base analogues (**5–22**).

**Method-B**: A 10 mL oven-dried round bottom flask was charged with 2 mol% DABCO-*C*_*7*_-F catalyst. Coumarin aldehyde (1 mmol), substituted phenylhydrazine hydrochlorides (1.1 mmol) were added using EtOH as solvent. The resulting reaction mixture was heated at 70–80 °C. The progress of the reaction was observed by TLC using a solvent system of EtOAc and petroleum ether (20%). Upon completion, the reaction mixture was cooled at room temperature. Later, crushed ice was added to the flask to quench the reaction resulting in the precipitation of the product. The obtained precipitates were then filtered and washed thoroughly with cold distilled water to remove the catalyst and get the pure desired products (**5–22**).

#### 3-((2′-((2-Phenylhydrazineylidene)methyl)phenoxy)methyl)-2*H*-chromen-2-one (5)

Yellow solid; Yield: 79% (Method-A)/ 88% (Method-B); m.p.: 178–180 °C; IR (*ν*_max_, cm^−1^): (KBr disc): 3264 (N‒H), 3053 (sp^2^ C‒H), 2884 (sp^3^ C‒H), 1720 (C=O), 1603, 1491 (C=C), 1283 (C‒O), 1225, 1188, 1123, 1079; UV (MeOH) nm: 353, 285, 259, 235, 216; ^1^H-NMR (400 MHz, DMSO-*d*_6_): *δ*_H_ 10.32 (s, 1H, *N*H-8′), 8.25 (1H, s, H-7′), 8.20 (s, 1H, H-4), 7.89 (dd, 1H, *J*_3′,4′_ = 8.0 Hz, *J*_3′,5′_ = 4.0 Hz, H-3′), 7.79 (dd, 1H, *J*_5,6_ = 8.0 Hz, *J*_5,7_ = 4.0 Hz, H-5), 7.65 (td, 1H, *J*_7,(6,8)_ = 8.0 Hz, *J*_7,5_ = 4.0 Hz, H-7), 7.47 (d, 1H, *J*_8,7_ = 8.0 Hz, H-8), 7.40 (td, 1H, *J*_6,(5,7)_ = 8.0 Hz, *J*_6,8_ = 4.0 Hz, H-6), 7.28 (td, 1H, *J*_5′,(4′,6′)_ = 8.0 Hz, *J*_5′,3′_ = 4.0 Hz, H-5′), 7.20–7.15 (m, 3H, H-6′, H-3′′/H-5′′), 7.03 (d, 3H, *J*_4′,(3′,5′)_ = 6.0 Hz, *J*_2′′,3′′/6′′,5′′_ = 8.0 Hz, H-4′, H-2′′/H-6′′), 6.76 (t, 1H, *J*_4′′,(3′′,5′′)_ = 8.0 Hz, H-4′′), 5.03 (s, 2H, C*H*_2_-9); EI-MS *m/z* (%): 370 (M^+^, 62), 278 (5), 263 (19), 211 (100), 184 (43), 159 (35), 115 (16), 77 (15), 51 (5); HREI-MS: calcd for C_23_H_18_N_2_O_3_ (M): *m/z* 370.1317, found: 370.1309.

#### 3-((2′-((2-(4′′-Fluorophenyl)hydrazineylidene)methyl)phenoxy)methyl)-2*H*-chromen-2-one (6)

Yellow solid; Yield: 73% (Method-A)/ 77% (Method-B); m.p.: 157–161 °C; IR (*ν*_max_, cm^−1^): (KBr disc): 3437 (N‒H), 3043 (sp^2^ C‒H), 2881 (sp^3^ C‒H), 1719 (C=O), 1605, 1489 (C=C), 1280 (C‒O), 1216 (C‒N), 1122, 1081; UV (MeOH) nm: 354, 315, 287, 257, 214; ^1^H-NMR (400 MHz, DMSO-*d*_6_): *δ*_H_ 10.31 (s, 1H, *N*H-8′), 8.23 (s, 1H, H-7′), 8.19 (s, 1H, H-4), 7.88 (dd, 1H, *J*_3′,4′_ = 8.0 Hz, *J*_3′,5′_ = 4.0 Hz, H-3′), 7.79 (dd, 1H, *J*_5,6_ = 8.0 Hz, *J*_5,7_ = 4.0 Hz, H-5), 7.65 (td, 1H, *J*_7,(6,8)_ = 8.0 Hz, *J*_7,5_ = 4.0 Hz, H-7), 7.47 (d, 1H, *J*_8,7_ = 8.0 Hz, H-8), 7.40 (td, 1H, *J*_6,(5,7)_ = 8.0 Hz, *J*_6,8_ = 4.0 Hz, H-6), 7.26 (td, 1H, *J*_5′,(4′,6′)_ = 8.0 Hz, *J*_5′,3′_ = 4.0 Hz, H-5′), 7.17 (d, 1H, *J*_6′,5′_ = 8.0 Hz, H-6′), 7.03–6.99 (m, 5H, H-4′, H-2′′/H-6′′, H-3′′/H-5′′), 5.03 (s, 2H, C*H*_2_-9); ^13^C-NMR (75 MHz, DMSO-*d*_6_): *δ*_C_ 159.6 (C-2), 157.3/154.3 (^1^*J* = 232.3 Hz, C-4′′), 155.2 (C-1′), 152.9 (C-8a), 142.0/141.91 (^4^*J* = 1.7 Hz, C-1′′), 140.6 (CH-4), 132.0 (CH-7), 131.9 (C-7′), 129.2 (C-5′), 128.6 (C-5), 124.8 (C-3′), 124.7 (C-6), 124.2 (C-2′), 124.0 (C-3), 121.3 (C-4′), 118.7 (C-4a), 116.2 (C-8), 115.7/115.5 (^2^*J* = 22.2 Hz, C-3′′/C-5′′), 113.0 (C-6′), 112.8/112.7 (^3^*J* = 7.2 Hz, C-2′′/C-6′′), 65.4 (C-9); EI-MS *m/z* (%): 388 (M^+^, 76), 278 (5), 263 (16), 229 (100), 202 (33), 159 (87), 115 (32), 77 (16), 51 (4); HREI-MS: calcd for C_23_H_17_N_2_O_3_F (M): *m/z* 388.1223, found: 388.1229.

#### 3-((2′-((2-(2′′-Chlorophenyl)hydrazineylidene)methyl)phenoxy)methyl)-2*H*-chromen-2-one (7)

White solid; Yield: 80% (Method-A)/ 96% (Method-B); m.p.: 182–185 °C; IR (*ν*_max_, cm^−1^): (KBr disc): 3305 (N‒H), 3061 (sp^2^ C‒H), 2877 (sp^3^ C‒H), 1722 (C=O), 1594, 1450 (C=C), 1246 (C‒O), 1176, 1096, 1021, 746 (C‒Cl); UV (MeOH) nm: 345, 213; ^1^H-NMR (400 MHz, DMSO-*d*_6_): *δ*_H_ 9.96 (s, 1H, *N*H-8′), 8.68 (1H, s, H-7′), 8.25 (s, 1H, H-4), 7.93 (dd, 1H, *J*_3′,4′_ = 8.6 Hz, *J*_3′,5′_ = 1.0 Hz, H-3′), 7.80 (dd, 1H, *J*_5,6_ = 8.3 Hz, *J*_5,7_ = 1.1 Hz, H-5), 7.64 (td, 1H, *J*_7,(6,8)_ = 8.8 Hz, *J*_7,5_ = 2.5 Hz, H-7), 7.56 (dd, 1H, *J*_3′′,4′′_ = 8.3 Hz, *J*_3′′,5′′_ = 0.9 Hz, H-3′′), 7.47 (d, 1H, *J*_8,7_ = 8.0 Hz, H-8), 7.40 (t, 1H, *J*_6,(5,7)_ = 8.3 Hz, H-6), 7.35–7.29 (m, 2H, H-5′, H-6′′), 7.22 (t, 1H, *J*_5′′,6′′_ = 9.0 Hz, H*-*5′′), 7.19 (d, 1H, *J*_6′,5′_ = 8.0 Hz, H-6′), 7.04 (t, 1H, *J*_4′,(3′,5′)_ = 4.8 Hz, H-4′), 6.77 (td, 1H, *J*_4′′,(3′′,5′′)_ = 8.2 Hz, *J*_4′′,6′′_ = 1.0 Hz, H-4′′), 5.04 (s, 2H, C*H*_2_-9); ^13^C-NMR (125 MHz, DMSO-*d*_6_): *δ*_C_159.5 (C-2), 155.6 (C-1′), 152.9 (C-8a), 141.5 (C-1′′), 140.3 (C-4), 135.7 (C-3′′), 131.9 (C-7), 129.8 (C-7′), 129.3 (C-5′), 128.6 (C-5), 127.9 (C-3′), 125.1 (C-6), 124.7 (C-2′), 124.0 (C-3), 121.3 (C-4′), 119.5 (C-5′′), 118.8 (C-4a), 116.2 (C-8), 116.0 (C-2′′), 114.0 (C-6′′), 113.0 (C-6′), 65.3 (C-9); EI-MS *m/z* (%): 406 (M^+^+2, 17), 404 (M^+^, 46), 278 (9), 263 (29), 245 (100), 210 (45), 159 (51), 115 (21), 77 (13); HREI-MS: calcd. for C_23_H_17_O_3_N_2_Cl (M^+^): *m/z* 404.0928, found: 404.0929.

#### 3-((2′-((2-(3′′-Chlorophenyl)hydrazineylidene)methyl)phenoxy)methyl)-2*H*-chromen-2-one (8)

White solid; Yield: 69% (Method-A)/ 87% (Method-B); m.p.: 157–159 °C; IR (*ν*_max_, cm^−1^): (KBr disc): 3445 (N‒H), 3074 (sp^2^ C‒H), 2875 (sp^3^ C‒H), 1722 (C=O), 1600, 1484 (C=C), 1289 (C‒O), 1174, 1135, 1082, 1015, 750 (C‒Cl); UV (MeOH) nm: 353, 216; ^1^H-NMR (400 MHz, DMSO-*d*_6_): *δ*_H_ 10.53 (s, 1H, *N*H-8′), 8.26 (s, 1H, H-7′), 8.19 (s, 1H, H-4), 7.90 (d, 1H, *J*_3′,4′_ = 7.6 Hz, H-3′), 7.78 (d, 1H, *J*_5,6_ = 7.6 Hz, H-5), 7.65 (t, 1H, *J*_7,(6,8)_ = 7.6 Hz, H-7), 7.47 (d, 1H, *J*_8,7_ = 8.0 Hz, H-8), 7.39 (t, 1H, *J*_6,(5,7)_ = 7.4 Hz, H-6), 7.31 (t, 1H, *J*_5′,(4′,6′)_ = 7.3 Hz, H-5′), 7.21–7.16 (m, 2H, H-6′, H-5′′), 7.06 (s, 1H, H*-*2′′), 7.03 (t, 1H, *J*_4′,(3′,5′)_ = 7.2 Hz, H-4′), 6.93 (d, 1H, *J*_4′′,(3′′,5′′)_ = 8.0 Hz, H-4′′), 6.74 (d, 1H, *J*_6′′,5′′_ = 7.2 Hz, H-6′′), 5.04 (s, 2H, C*H*_2_-9); ^13^C-NMR (125 MHz, DMSO-*d*_6_): *δ*_C_ 159.2 (C-2), 155.4 (C-1′), 152.9 (C-8a), 146.7 (C-1′′), 140.7 (C-4), 133.8 (C-3′′), 133.4 (C-5′′), 132.0 (C-7), 130.7 (C-7′), 129.6 (C-5′), 128.6 (C-5), 124.9, (C-3′), 124.8 (C-6), 123.9 (C-2′), 123.8 (C-3), 121.4 (C-4′), 118.7 (C-4a), 117.9 (C-4′′), 116.2 (C-8), 113.0 (C-6′), 111.0 (C-2′′), 110.6 (C-6′′), 65.4 (C-9); EI-MS *m/z* (%): 406 (M^+^+2, 8), 404 (M^+^, 24), 278 (6), 263 (17), 247 (35), 245 (100), 159 (63), 115 (24), 77 (13), 51 (5); HREI-MS: calcd. for C_23_H_17_O_3_N_2_Cl (M^+^): *m/z* 404.0928, found: 404.0923.

#### 3-((2′-((2-(4′′-Chlorophenyl)hydrazineylidene)methyl)phenoxy)methyl)-2*H*-chromen-2-one (9)

Yellow solid; Yield: 87% (Method-A)/ 96% (Method-B); m.p.: 203–205 °C; IR (*ν*_max_, cm^−1^): (KBr disc): 3250 (N‒H), 3038 (sp^2^ C‒H), 2876 (sp^3^ C‒H), 1710 (C=O), 1595, 1486 (C=C), 1280 (C‒O), 1086, 746 (C‒Cl); UV (MeOH) nm: 355, 316, 263, 231, 213; ^1^H-NMR (400 MHz, DMSO-*d*_6_): *δ*_H_ 10.46 (s, 1H, *N*H-8′), 8.25 (1H, s, H-7′), 8.19 (s, 1H, H-4), 7.88 (dd, 1H, *J*_3′,4′_ = 8.3 Hz, *J*_3′,5′_ = 0.9 Hz, H-3′), 7.79 (dd, 1H, *J*_5,6_ = 8.6 Hz, *J*_5,7_ = 1.3 Hz, H-5), 7.65 (td, 1H, *J*_7,(6,8)_ = 8.1 Hz, *J*_7,5_ = 2.0 Hz, H-7), 7.47 (d, 1H, *J*_8,7_ = 8.2 Hz, H-8), 7.39 (t, 1H, *J*_6,(5,7)_ = 7.1 Hz, H-6), 7.30 (td, 1H, *J*_5′,(4′,6′)_ = 9.0 Hz, *J*_5′,3′_ = 1.3 Hz, H-5′), 7.22 (d, 2H, *J*_3′′,2′′/5′′,6′′_ = 8.7 Hz, H-3′′/H*-*5′′), 7.17 (d, 1H, *J*_6′,5′_ = 8.4 Hz, H-6′), 7.04 (*app*. t, 3H, *J*_4′(3′,5′)/2′′,3′′/6′′,5′′_ = 12.0 Hz, H-4′, H-2′′/H-6′′), 5.03 (s, 2H, C*H*_2_-9); ^13^C-NMR (125 MHz, DMSO-*d*_6_): *δ*_C_ 159.6 (C-2), 155.3 (C-1′), 152.9 (C-8a), 144.2 (C-1′′), 140.7 (C-4), 132.7 (C-3′′/C-5′′), 132.0 (C-7), 129.4 (C-7′), 128.9 (C-5′), 128.6 (C-5), 124.8 (C-6), 124.7 (C-2′), 124.0 (C-3), 123.9 (C-4′′), 121.8 (C-2′′/C-6′′), 121.3 (C-4′), 118.7 (C-4a), 116.2 (C-8), 113.3 (C-8), 113.0 (C-6′), 65.3 (C-9); EI-MS *m/z* (%): 406 (M^+^+2, 8), 404 (M^+^, 25), 278 (2), 263 (8), 245 (100), 247 (31), 210 (25), 159 (33), 115 (12), 77 (8), 51 (2); HREI-MS: calcd. for C_23_H_17_O_3_N_2_Cl (M^+^): *m/z* 404.0928, found: 404.0934.

#### 3-((2′-((2-(2′′-Bromophenyl)hydrazineylidene)methyl)phenoxy)methyl)-2*H*-chromen-2-one (10)

Off-white solid; Yield: 76% (Method-A)/ 94% (Method-B); m.p.: 208–210 °C; IR (*ν*_max_, cm^−1^): (KBr disc): 3425 (N‒H), 3092 (sp^2^ C‒H), 2878 (sp^3^ C‒H), 1724 (C=O), 1604, 1444 (C=C), 1247 (C‒O), 1178, 1090, 1019; UV (MeOH) nm: 346, 215; ^1^H-NMR (400 MHz, DMSO-*d*_6_): *δ*_H_ 9.76 (s, 1H, *N*H-8′), 8.69 (s, 1H, H-7′), 8.25 (s, 1H, H-4), 7.93 (dd, 1H, *J*_3′,4′_ = 8.1 Hz, *J*_3′,5′_ = 1.1 Hz, H-3′), 7.80 (d, 1H, *J*_5,6_ = 7.4 Hz, H-5), 7.64 (td, 1H, *J*_7,(6,8)_ = 8.0 Hz, *J*_7,5_ = 1.4 Hz, H-7), 7.54 (d, 1H, *J*_3′′,4′′_ = 7.0 Hz, H-3′′), 7.47 (d, 2H, *J*_8,7/6′′,5′′_ = 8.5 Hz, H-8, H-6′′), 7.40 (t, 1H, *J*_6,(5,7)_ = 8.0 Hz, H-6), 7.33 (t, 1H, *J*_5′′,(4′′,6′′)_ = 8.5 Hz, H-5′′), 7.26 (t, 1H, *J*_5′,(4′,6′)_ = 8.2 Hz, H-5′), 7.19 (d, 1H, *J*_6′,5′_ = 8.1 Hz, H-6′), 7.03 (t, 1H, *J*_4′,(3′,5′)_ = 9.0 Hz, H-4′), 6.72 (td, 1H, *J*_4′′,(3′′,5′′)_ = 8.8 Hz, *J*_4′′,6′′_ = 1.2 Hz, H-4′′), 5.04 (s, 2H, C*H*_2_-9); ^13^C-NMR (75 MHz, DMSO-*d*_6_): *δ*_C_ 159.6 (C-2), 155.6 (C-1′), 152.9 (C-8a), 142.5 (C-1′′), 140.4 (C-4), 135.8 (C-2′′), 132.6 (C-6′′), 131.9 (C-7), 129.9 (C-7′), 128.6 (C-5′, C-5), 128.5 (C-3′), 125.2 (C-6), 124.8 (C-2′), 124.0 (C-3), 121.4 (C-4′), 120.2 (C-3′′), 118.8 (C-4a), 116.2 (C-8), 114.6 (C-4′′), 113.0 (C-6′), 105.9 (C-5′′), 65.3 (C-9); EI-MS *m/z* (%): 450 (M^+^+2, 36), 448 (M^+^, 37), 291 (77), 289 (83), 278 (10), 210 (100), 159 (72), 159 (72), 115 (31), 77 (18), 51 (7); HREI-MS: calcd. for C_23_H_17_O_3_N_2_Br (M^+^): *m/z* 448.0423, found: 448.0419.

#### 3-((2′-((2-(4′′-Bromophenyl)hydrazineylidene)methyl)phenoxy)methyl)-2*H*-chromen-2-one (11)

Yellow solid; Yield: 89% (Method-A)/ 94% (Method-B); m.p.: 208–210 °C; IR (*ν*_max_, cm^−1^): (KBr disc): 3249 (N‒H), 1711 (C=O), 1642, 1586, 1527, 1481, 1456, 1441 (C=C), 1407, 1388, 1316, 1279 (C‒O), 1255, 1229, 1187, 1121, 1064; UV (MeOH) nm: 357, 215; ^1^H-NMR (400 MHz, DMSO-*d*_6_): *δ*_H_ 10.47 (s, 1H, *N*H-8′), 8.25 (s, 1H, H-7′), 8.19 (s, 1H, H-4), 7.88 (dd, 1H, *J*_3′,4′_ = 8.0 Hz, *J*_3′,5′_ = 1.2 Hz, H-3′), 7.79 (dd, 1H, *J*_5,6_ = 8.0 Hz, *J*_5,7_ = 4.0 Hz, H-5), 7.64 (td, 1H, *J*_7,(6,8)_ = 10.0 Hz, *J*_7,5_ = 4.0 Hz, H-7), 7.47 (d, 1H, *J*_8,7_ = 8.0 Hz, H-8), 7.39 (t, 1H, *J*_6,(5,7)_ = 8.0 Hz, H-6), 7.34–7.28 (m, 3H, *J*_5′,(4′,6′)_ = 6.0 Hz, *J*_3′′,2′′/5′′,6′′_ = 6.0 Hz, H-5′, H-3′′/H-5′′), 7.17 (d, 1H, *J*_6′,5′_= 8.0 Hz, H-6′), 7.03–6.97 (d + t, 3 H, *J*_4′,(3′,5′)_ = 6.0 Hz, *J*_2′′,3′′/6′′,5′′_ = 6.0 Hz, H-4′, H-2′′/H-6′′), 5.03 (s, 2 H, C*H*_2_-9); EI-MS *m/z* (%): 450 (M^+^+2, 32), 448 (M^+^, 36), 329 (3), 293 (2), 291 (74), 263 (17), 210 (100), 182 (27), 159 (89), 115 (29), 77 (17), 51 (6); HREI-MS: calcd. for C_23_H_17_O_3_N_2_Br (M^+^): *m/z* 448.0423, found: 448.0384.

#### 3-((2′-((2-(*o*-Tolyl)hydrazineylidene)methyl)phenoxy)methyl)-2*H*-chromen-2-one (**12**)

Off-white solid; Yield: 79% (Method-A)/ 90% (Method-B); m.p.: 182–184 °C; IR (*ν*_max_, cm^−1^): (KBr disc): 3427 (N‒H), 3068 (sp^2^ C‒H), 2874 (sp^3^ C‒H), 1724 (C=O), 1601, 1454 (C=C), 1253 (C‒O), 1177 (C‒O), 1146, 1109, 1086, 1019; UV (MeOH) nm: 352, 285, 258, 234, 213; ^1^H-NMR (400 MHz, DMSO-*d*_6_): *δ*_H_ 9.59 (s, 1H, *N*H-8′), 8.51 (s, 1H, H-7′), 8.22 (s, 1H, H-4), 7.92 (dd, 1H, *J*_3′,4′_ = 8.8 Hz, *J*_3′,5′_ = 1.9 Hz, H-3′), 7.79 (dd, 1H, *J*_5,6_ = 8.2 Hz, *J*_5,7_ = 1.3 Hz, H-5), 7.64 (td, 1H, *J*_7,(6,8)_ = 9.1 Hz, *J*_7,5_ = 1.0 Hz, H-7), 7.47 (d, 1H, *J*_8,7_ = 8.3 Hz, H-8), 7.39 (*app*. t, 2H, *J*_6,(5,7)/3′′,4′′_ = 8.4 Hz, H-6, H-3′′), 7.29 (td, 1H, *J*_5′,(4′,6′)_ = 9.1 Hz, *J*_5′,3′_ = 1.1 Hz, H-5′), 7.17 (d, 1H, *J*_6′,5′_ = 8.2 Hz, H-6′), 7.07 (t, 1H, *J*_5′′,(4′′,6′′)_ = 7.9 Hz, H-5′′), 7.02 (d + d, 2H, *J*_4′,3′/6′′,5′′_ = 8.0 Hz, H-4′, H*-*6′′), 6.68 (t, 1H, *J*_4′′,(3′′,5′′)_ = 7.6 Hz, H*-*4′′), 5.04 (s, 2H, C*H*_2_-9), 2.19 (s, 3H, C*H*_3_*-*7′′); EI-MS *m/z* (%): 384 (M^+^, 74), 278 (5), 263 (22), 225 (100), 210 (12), 159 (41), 115 (18), 77 (22), 51 (6); HREI-MS: calcd. for C_24_H_20_O_3_N_2_ (M^+^): *m/z* 384.1474, found: 384.1443.

#### 3-((2′-((2-(*p*-Tolyl)hydrazineylidene)methyl)phenoxy)methyl)-2*H*-chromen-2-one (**13**)

Off-white solid; Yield: 88% (Method-A)/ 95% (Method-B); m.p.: 175–178 °C; IR (*ν*_max_, cm^−1^): (KBr disc): 3426 (N‒H), 3021 (sp^2^ C‒H), 2887 (sp^3^ C‒H), 1724 (C=O), 1605, 1520 (C=C), 1253 (C‒O), 1173, 1139, 1096, 1045, 1015; UV (MeOH) nm: 358, 269; ^1^H-NMR (400 MHz, DMSO-*d*_6_): *δ*_H_ 10.21 (s, 1H, *N*H-8′), 8.21 (s, 1H, H-7′), 8.19 (s, 1H, H-4), 7.87 (dd, 1H, *J*_3′,4′_ = 8.0 Hz, *J*_3′,5′_ = 1.7 Hz, H-3′), 7.79 (dd, 1H, *J*_5,6_ = 8.0 Hz, *J*_5,7_ = 1.4 Hz, H-5), 7.64 (td, 1H, *J*_7,(6,8)_ = 9.0 Hz, *J*_7,5_ = 4.0 Hz, H-7), 7.46 (d, 1H, *J*_8,7_ = 8.0 Hz, H-8), 7.39 (td, 1H, *J*_6,(5,7)_ = 8.0 Hz, *J*_6,8_ = 1.3 Hz, H-6), 7.25 (td, 1H, *J*_5′,(4′,6′)_ = 12.0 Hz, *J*_5′,3′_ = 2.0 Hz, H-5′), 7.15 (d, 1H, *J*_6′,5′_ = 8.0 Hz, H-6′), 7.02–6.99 (m, 3H, H-4′, H-3′′/H-5′′), 6.93 (d, 2H, *J*_2′′,3′′/6′′,5′′_ = 8.0 Hz, H-2′′/H*-*6′′), 5.03 (s, 2H, C*H*_2_-9), 2.19 (s, 3H, C*H*_3_*-*7′′); ^13^C-NMR (75 MHz, DMSO-*d*_6_): *δ*_C_ 159.6 (C-2), 155.1 (C-1′), 152.9 (C-8a), 143.1 (C-1′′), 140.5 (CH-4), 132.0 (CH-7), 131.2 (C-7′), 129.5 (C-3′′/C-5′′), 128.9 (C-5′), 128.6 (C-5), 127.1 (C-4′′), 124.8 (C-3′), 124.6 (C-6), 124.5 (C-2′), 124.1 (C-3), 121.3 (C-4′), 118.8 (C-4a), 116.2 (C-8), 112.9 (C-6′), 111.9 (C-2′′/C-6′′), 65.3 (C-9), 20.2 (C*-*7′′); EI-MS *m/z* (%): 384 (M^+^, 56), 278 (4), 263 (15), 225 (100), 210 (15), 159 (78), 115 (26), 77 (25); HREI-MS: calcd. for C_24_H_20_O_3_N_2_ (M^+^): *m/z* 384.1474, found: 384.1475.

#### 3-((2′-((2-(2′′-Ethylphenyl)hydrazineylidene)methyl)phenoxy)methyl)-2*H*-chromen-2-one (**14**)

Off-white solid; Yield: 72% (Method-A)/ 84% (Method-B); m.p.: 140–143 °C; IR (*ν*_max_, cm^−1^): (KBr disc): 3057 (N‒H), 2970 (sp^2^ C‒H), 2876 (sp^3^ C‒H), 1725 (C=O), 1592, 1451 (C=C), 1248 (C‒O), 1179, 1098, 1021; UV (MeOH) nm: 351, 283, 215; ^1^H-NMR (400 MHz, DMSO-*d*_6_): *δ*_H_ 9.65 (s, 1H, *N*H-8′), 8.51 (s, 1H, H-7′), 8.21 (s, 1H, H-4), 7.91 (dd, 1H, *J*_3′,4′_ = 8.2 Hz, *J*_3′,5′_ = 1.4 Hz, H-3′), 7.78 (dd, 1H, *J*_5,6_ = 7.6 Hz, *J*_5,7_ = 1.2 Hz, H-5), 7.64 (td, 1H, *J*_7,(6,8)_ = 8.0 Hz, *J*_7,5_ = 1.8 Hz, H-7), 7.47–7.42 (m, 2H, H-8, H-5′′), 7.39 (t, 1H, *J*_6,(5,7)_ = 9.0 Hz, H-6), 7.28 (td, 1H, *J*_5′,(4′,6′)_ = 8.5 Hz, *J*_5′,3′_ = 1.6 Hz, H-5′), 7.17 (d, 1H, *J*_6′,5′_ = 8.4 Hz, H-6′), 7.09-7.00 (m, 3H, H-4′, H-3′′, H-6′′), 6.72 (t, 1H, *J*_4′′,(3′′,5′′)_ = 7.2 Hz, H-4′′), 5.05 (s, 2H, C*H*_2_-9), 2.59 (q, 2H, *J*_7′′,8′′_ = 14.8 Hz, C*H*_2_-7′′), 1.15 (t, 3H, *J*_8′′,7′′_ = 7.6 Hz, C*H*_3_-8′′); ^13^C-NMR (75 MHz, DMSO-*d*_6_): *δ*_C_ 159.6 (C-2), 155.3 (C-1′), 152.9 (C-8a), 142.5 (C-1′′), 140.6 (C-4), 133.1 (C-3′′), 132.0 (C-7), 129.2 (C-7′), 128.6 (C-5′), 128.3 (C-5), 126.6 (C-6′′), 126.5 (C-5′′), 124.8 (C-3′), 124.7 (C-6), 124.5 (C-2′), 124.0 (C-3), 121.3 (C-4′), 118.9 (C-4a), 118.7 (C-2′′), 116.2 (C-8), 112.9 (C-6′), 112.5 (C-4′′), 65.3 (C-9), 20.1 (C-7′′), 13.9 (C-8′′); EI-MS *m/z* (%): 398 (M^+^, 56), 239 (100), 210 (9), 159 (20), 77 (11), 51 (2); HREI-MS: calcd. for C_25_H_22_O_3_N_2_ (M^+^): *m/z* 398.1630, found: 398.1614.

#### 3-((2′-((2-(4′′-Methoxyphenyl)hydrazineylidene)methyl)phenoxy)methyl)-2*H*-chromen-2-one (**15**)

Yellow solid; Yield: 85% (Method-A)/ 89% (Method-B); m.p.: 165–168 °C; IR (*ν*_max_, cm^-1^): (KBr disc): 3265 (N‒H), 3046 (sp^2^ C‒H), 2827 (sp^3^ C‒H), 1720 (C=O), 1607, 1497 (C=C), 1235 (C‒O), 1184, 1121, 1039, 1002; UV (MeOH) nm: 363, 316, 259, 213; ^1^H-NMR (400 MHz, DMSO-*d*_6_): *δ*_H_ 10.11 (s, 1H, *N*H-8′), 8.19 (s, 2H, H-4, H-7′), 7.86 (dd, 1H, *J*_3′,4′_ = 7.6 Hz, *J*_3′,5′_ = 1.2 Hz, H-3′), 7.79 (dd, 1H, *J*_5,6_ = 8.4 Hz, *J*_5,7_ = 1.0 Hz, H-5), 7.64 (td, 1H, *J*_7,(6,8)_ = 8.4 Hz, *J*_7,5_ = 1.1 Hz, H-7), 7.47 (d, 1H, *J*_8,7_ = 8.0 Hz, H-8), 7.39 (td, 1H, *J*_6,(5,7)_ = 7.6 Hz, *J*_6,8_ = 1.2 Hz, H-6), 7.26 (td, 1H, *J*_5′,(4′,6′)_ = 8.4 Hz, *J*_5′,3′_ = 1.4 Hz, H-5′), 7.15 (d, 1H, *J*_6′,5′_ = 8.4 Hz, H-6′), 7.02–6.96 (d + t, 3H, *J*_4′,(3′,5′)_ = 7.6 Hz, *J*_2′′,3′′/6′′,5′′_ = 9.0 Hz, H-4′, H-2′′/H-6′′), 6.80 (d, 2H, *J*_3′′,2′′/5′′,6′′_ = 8.8 Hz, H-3′′/H-5′′), 5.02 (s, 2H, C*H*_2_-9), 3.67 (s, 3H, OC*H*_3_-7′′); EI-MS *m/z* (%): 400 (M^+^, 31), 384 (1), 263 (4), 241 (100), 159 (43), 115 (16), 77 (12), 51 (4); HREI-MS: calcd. for C_24_H_20_O_4_N_2_ (M^+^): *m/z* 400.1423, found: 400.1411.

#### 4′′-(2-(2′-((2-Oxo-2*H*-chromen-3-yl)methoxy)benzylidene)hydrazineyl)benzonitrile (**16**)

Off white solid; Yield: 71% (Method-A)/ 79% (Method-B); m.p.: 255–257 °C; IR (*ν*_max_, cm^-1^): (KBr disc): 3282 (N‒H), 3041 (sp^2^ C‒H), 2809 (sp^3^ C‒H), 2211 (C ≡ N), 1728 (C=O), 1609, 1535, 1489 (C=C), 1453, 1366, 1331, 1256 (C‒O), 1216, 1170, 1089, 1023; UV (MeOH) nm: 358, 214; ^1^H-NMR (300 MHz, DMSO-*d*_6_): *δ*_H_ 10.94 (s, 1H, *N*H-8′), 8.35 (s,1H, H-7′), 8.20 (s, 1H, H-4), 7.91 (d, 1H, *J*_3′,4′_ = 6.6 Hz, H-3′), 7.79 (d, 1H, *J*_5,6_ = 6.9 Hz, H-5), 7.65 (t, 1H, *J*_7,(6,8)_ = 7.2 Hz, H-7), 7.60 (m, 2H, *J*_3′′,2′′/5′′,6′′_ = 8.2 Hz, H-3′′/H-5′′), 7.47 (d, 1H, *J*_8,7_ = 8.1 Hz, H-8), 7.42–7.32 (m, 2H, H-6, H*-*5′), 7.20 (d, 1H, *J*_6′,5′_ = 8.4 Hz, H-6′), 7.12 (d, 2H, *J*_2′′,3′′/6′′,5′′_ = 8.7 Hz, H-2′′/H-6′′), 7.04 (t, 1H, *J*_4′,(3′,5′)_ = 7.5 Hz, H-4′), 5.05 (s, 2H, C*H*_2_-9); EI-MS *m/z* (%): 395 (M^+^, 52), 278 (5), 263 (11), 236 (79), 159 (100), 115 (19), 77 (12); HREI-MS: calcd. for C_24_H_17_O_3_N_3_ (M^+^): *m/z* 395.1270, found: 395.1256.

#### 3-((2′-((2-(2′′-Nitrophenyl)hydrazineylidene)methyl)phenoxy)methyl)-2*H*-chromen-2-one (**17**)

Yellow solid; Yield: 63% (Method-A)/ 73% (Method-B); m.p.: 202–205 °C; (IR (*ν*_max_, cm^−1^): (KBr disc) IR (*ν*_max_, cm^−1^): (KBr disc): 3427 (N‒H), 3036 (sp^2^ C‒H), 2883 (sp^3^ C‒H), 1722 (C=O), 1613, 1493 (C=C), 1259 (C‒O), 1218, 1175, 1137, 1073, 1018; UV (MeOH) nm: 444, 327, 274, 215; ^1^H-NMR (400 MHz, DMSO-*d*_6_): *δ*_H_ 11.28 (s, 1H, *N*H-8′), 8.86 (s, 1H, H-7′), 8.29 (s, 1H, H-4), 8.09 (dd, 1H, *J*_3′′,4′′_ = 9.0 Hz, *J*_3′′,5′′_ = 1.2 Hz, H-3′′), 7.99–7.97 (m, 2H, H-3′, H-5′′), 7.81 (d, 1H, *J*_5,6_ = 8.0 Hz, H-5), 7.66–7.60 (m, 2H, H-7, H-6′′), 7.47 (d, 1H, *J*_8,7_ = 9.0 Hz, H-8), 7.40 (t, 2H, *J*_6,(5,7)/5′,(4′,6′)_ = 8.0 Hz, H-6, H-5′), 7.23 (d, 1H, *J*_6′,5′_ = 8.0 Hz, H-6′), 7.07 (t, 1H, *J*_4′,(3′,5′)_ = 10.0 Hz, H-4′), 6.90 (t, 1H, *J*_4′′,(3′′,5′′)_ = 8.6 Hz, H-4′′), 5.07 (s, 2H, C*H*_2_-9); EI-MS *m/z* (%): 415 (M^+^, 31), 295 (7), 278 (5), 263 (33), 256 (49), 239 (5), 210 (27), 210 (27), 159 (100), 115 (34), 77 (11), 51 (4); HREI-MS: calcd. for C_23_H_17_O_5_N_3_ (M^+^): *m/z* 415.1168, found: 415.1174.

#### 3-((2′-((2-(3′′-Nitrophenyl)hydrazineylidene)methyl)phenoxy)methyl)-2*H*-chromen-2-one (**18**)

Orange solid; Yield: 70% (Method-A)/ 76% (Method-B); m.p.: 213–215 °C; IR (*ν*_max_, cm^−1^): (KBr disc): 3296 (N‒H), 3064 (sp^2^ C‒H), 2910 (sp^3^ C‒H), 1714 (C=O), 1608, 1543 (C=C), 1249 (C‒O), 1172, 1096, 1021; UV (MeOH) nm: 339, 273, 212; ^1^H-NMR (400 MHz, DMSO-*d*_6_): *δ*_H_ 10.84 (s, 1H, *N*H-8′), 8.33 (s, 1H, H-7′), 8.20 (s, 1H, H-4), 7.92 (dd, 1H, *J*_3′,4′_ = 8.5 Hz, *J*_3′,5′_ = 1.1 Hz, H-3′), 7.78 (dd + s, 2H, *J*_5,6_ = 8.1 Hz, *J*_5,7_ = 1.4 Hz, H-5, H-2′′), 7.65 (td, 1H, *J*_7,(6,8)_ = 8.3 Hz, *J*_7,5_ = 1.7 Hz, H-7), 7.55 (dd, 1H, *J*_4′′,5′′_ = 8.1 Hz, *J*_4′′,5′′_ = 1.3 Hz, H-4′′), 7.48–7.44 (m, 2H, H-8, H-6′′), 7.41–7.38 (m, 2H, H-6, H-5′′), 7.34 (td, 1H, *J*_5′,(4′,6′)_ = 8.3 Hz, H-5′), 7.21 (d, 1H, *J*_6′,5′_ = 8.0 Hz, H-6′), 7.06 (t, 1H, *J*_4′,(3′,5′)_ = 8.1 Hz, *H*-4′), 5.07 (s, 2H, C*H*_2_-9); EI-MS *m/z* (%): 415 (M^+^, 27), 278 (7), 263 (11), 256 (70), 210 (21), 159 (100), 115 (22), 77 (8), 51 (3); HREI-MS: calcd. for C_23_H_17_O_5_N_3_ (M^+^): *m/z* 415.1168, found: 415.1168.

#### 3-((2′-((2-(2′′,3′′-Dimethylphenyl)hydrazineylidene)methyl)phenoxy)methyl)-2*H*-chromen-2-one (**19**)

Yellow solid; Yield: 74% (Method-A)/ 83% (Method-B); m.p.: 179–182 °C; IR (*ν*_max_, cm^−1^): (KBr disc): 3428 (N‒H), 3068 (sp^2^ C‒H), 2876 (sp^3^ C‒H), 1725 (C=O), 1598, 1446 (C=C), 1252 (C‒O), 1175, 1144, 1106, 1019; UV (MeOH) nm: 352, 258, 212; ^1^H-NMR (400 MHz, DMSO-*d*_6_): *δ*_H_ 9.57 (s, 1H, *N*H-8′), 8.50 (s, 1H, H-7′), 8.22 (s, 1H, H-4), 7.91 (dd, 1H, *J*_3′,4′_ = 8.3 Hz, *J*_3′,5′_ = 1.1 Hz, H-3′), 7.79 (dd, 1H, *J*_5,6_ = 8.0 Hz, *J*_5,7_ = 1.3 Hz, H-5), 7.64 (td, 1H, *J*_7,(6,8)_ = 8.1 Hz, *J*_7,5_ = 1.1 Hz, H-7), 7.47 (d, 1H, *J*_8,7_ = 8.3 Hz, H-8), 7.39 (t, 1H, *J*_6,(5,7)_ = 8.0 Hz, H-6), 7.27 (d + t, 2H, *J*_5′,(4′,6′)/4′′,5′′_ = 8.5 Hz, H-5′, H-4′′), 7.16 (d, 1H, *J*_6′,5′_ = 8.0 Hz, H-6′), 7.01 (t, 1H, *J*_4′,(3′,5′)_ = 8.7 Hz, H-4′), 6.96 (t, 1H, *J*_5′′,(4′′,6′′)_ = 8.6 Hz, H-5′′), 6.62 (d, 1H, *J*_6′′,5′′_ = 8.3 Hz, H-6′′), 5.04 (s, 2H, C*H*_2_-9), 2.20 (s, 3H, C*H*_3_-7′′), 2.09 (s, 3H, C*H*_3_-8′′); ^13^C-NMR (75 MHz, DMSO-*d*_6_): *δ*_C_ 159.6 (C-2), 155.2 (C-1′), 152.9 (C-8a), 143.2 (C-1′′), 140.5 (C-4), 136.3 (C-3′′), 133.0 (C-7), 131.9 (C-7′), 129.1 (C-5′), 128.6 (C-5), 125.9 (C-5′′), 124.8 (C-3′), 124.7 (C-6), 124.5 (C-2′), 124.0 (C-3), 121.3 (C-4′), 120.8 (C-6′′), 119.1 (C-2′′), 118.8 (C-4a), 116.1 (C-8), 112.9 (C-6′), 110.6 (C-4′′), 65.3 (C-9), 20.2 (C-8′′), 12.7 (C-7′′); EI-MS *m/z* (%): 398 (M^+^, 37), 278 (3), 263 (11), 239 (100), 159 (35), 115 (15), 77 (20), 51 (5); HREI-MS: calcd. for C_25_H_22_O_3_N_2_ (M^+^): *m/z* 398.1630, found: 398.1641.

#### 3-((2′-((2-(2′′,4′′-Dimethylphenyl)hydrazineylidene)methyl)phenoxy)methyl)-2*H*-chromen-2-one (**20**)

Yellow solid; Yield: 76% (Method-A)/ 86% (Method-B); m.p.: 176–178 °C; IR (*ν*_max_, cm^-1^): (KBr disc): 3418 (N‒H), 3048 (sp^2^ C‒H), 2873 (sp^3^ C‒H), 1721 (C=O), 1605, 1448 (C=C), 1251 (C‒O), 1175, 1153, 1093, 1020; UV (MeOH) nm: 357, 258, 212; ^1^H-NMR (400 MHz, DMSO-*d*_6_): *δ*_H_ 9.50 (s, 1H, *N*H-8′), 8.46 (s, 1H, H-7′), 8.21 (s, 1H, H-4), 7.89 (dd, 1H, *J*_3′,4′_ = 8.0 Hz, *J*_3′,5′_ = 1.1 Hz, H-3′), 7.78 (d, 1H, *J*_5,6_ = 8.1 Hz, H-5), 7.64 (td, 1H, *J*_7,(6,8)_ = 8.0 Hz, *J*_7,5_ = 1.1 Hz, H-7), 7.47 (d, 1H, *J*_8,7_ = 8.3 Hz, H-8), 7.39 (t, 1H, *J*_6,(5,7)_ = 8.4 Hz, H-6), 7.30–7.25 (m, 2H, H-5′, H-5′′), 7.16 (d, 1H, *J*_6′,5′_ = 8.7 Hz, H-6′), 7.01 (t, 1H, *J*_4′,(3′,5′)_ = 8.0 Hz, H-4′), 6.89 (d, 1H, *J*_6′′,5′′_ = 8.6 Hz, H-6′′), 6.85 (s, 1H, H-3′′), 5.03 (s, 2H, C*H*_2_-9), 2.17 (s, 3H, CH_3_-7′′), 2.17 (s, 3H, CH_3_-8′′); EI-MS *m/z* (%): 398 (M^+^, 37), 279 (4), 263 (13), 239 (100), 159 (54), 77 (23), 51 (6); HREI-MS: calcd. for C_25_H_22_O_3_N_2_ (M^+^): *m/z* 398.1630, found: 398.1638.

#### 3-((2′-((2-(3′′,4′′-Dichlorophenyl)hydrazineylidene)methyl)phenoxy)methyl)-2*H*-chromen-2-one (**21**)

Yellow solid; Yield: 82% (Method-A)/ 85% (Method-B); m.p.: 183–185 °C; IR (*ν*_max_, cm^−1^): (KBr disc): 3428 (N‒H), 3060 (sp^2^ C‒H), 2874 (sp^3^ C‒H), 1696 (C=O), 1596, 1448 (C=C), 1250 (C‒O), 1181, 1136, 1086, 1031, 751 (C‒Cl); UV (MeOH) nm: 348, 215; ^1^H-NMR (400 MHz, DMSO-*d*_6_): *δ*_H_ 10.17 (s, 1H, *N*H-8′), 8.72 (1H, s, H-7′), 8.24 (s, 1H, H-4), 7.95 (dd, 1H, *J*_3′,4′_ = 8.6 Hz, *J*_3′,5′_ = 1.2 Hz, H-3′), 7.79 (dd, 1H, *J*_5,6_ = 8.1 Hz, *J*_5,7_ = 1.6 Hz, H-5), 7.64 (td, 1H, *J*_7,(6,8)_ = 8.2 Hz, *J*_7,5_ = 1.1 Hz, H-7), 7.51 (d, 1H, *J*_2′′,6′′_ = 2.0 Hz, H-2′′), 7.47 (d, 1H, *J*_8,7_ = 8.0 Hz, H-8), 7.40 (t, 1H, *J*_6,(5,7)_ = 7.4 Hz, H-6), 7.35–7.33 (d + m, 1H, *J*_5′′,6′′_ = 7.3 Hz, H-5′, H-5′′), 7.20 (d, 1H, *J*_6′,5′_ = 6.0 Hz, H-6′), 7.05 (t, 1H, *J*_4′,(3′,5′)_ = 6.0 Hz, H-4′), 6.80 (dd, 1H, *J*_6′′,5′′_ = 6.0 Hz, *J*_6′′,2′′_ = 1.5 Hz, H-6′′), 5.04 (s, 2H, C*H*_2_-9); ^13^C-NMR (125 MHz, DMSO-*d*_6_): *δ*_C_ 159.5 (C-2), 155.8 (C-1′), 152.9 (C-8a), 142.5 (C-1′′), 140.5 (C-4), 137.3 (CH-5′′), 132.7 (C-3′′), 131.9 (C-7), 130.8 (C-7′), 130.3 (C-5′), 128.6 (C-5), 125.3 (C-3′), 124.7 (C-6), 123.9 (C-2′), 123.6 (C-3), 121.4 (C-4′), 118.8 (C-4a), 116.2 (C-8), 114.6 (C-4′′), 113.1 (C-6′), 113.0 (C-2′′, C-6′′), 65.3 (C-9); EI-MS *m/z* (%): 442 (M + 4, 7), 440 (M + 2, 37), 438 (M^+^, 52), 281 (60), 279 (92), 263 (36), 244 (38), 209 (8), 159 (100), 115 (45), 77 (18), 51 (6); HREI-MS: calcd. for C_23_H_16_O_3_N_2_Cl_2_ (M^+^): *m/z* 438.0538, found: 438.0541.

#### 3-((2′-((2-(2′′,4′′-Dinitrophenyl)hydrazineylidene)methyl)phenoxy)methyl)-2*H*-chromen-2-one (**22**)

Yellow solid; Yield: 62% (Method-A)/ 71% (Method-B); m.p.: 254–256 °C; IR (*ν*_max_, cm^-1^): (KBr disc): 3428 (N‒H), 3044 (sp^2^ C‒H), 2875 (sp^3^ C‒H), 1703 (C=O), 1613, 1508 (C=C), 1255 (C‒O), 1187, 1133, 1083, 1018; UV (MeOH) nm: 354, 258, 216; ^1^H-NMR (400 MHz, DMSO-*d*_6_): *δ*_H_ 11.78 (s, 1H, *N*H-8′), 9.08 (s, 1H, H-7′), 8.86 (d, 1H, *J*_3′′,5′′_ = 2.4 Hz, H-3′′), 8.34 (dd, 1H, *J*_5′′,6′′_ = 9.6 Hz, *J*_5′′,3′′_ = 2.4 Hz, H-5′′), 8.29 (s, 1H, H-4), 8.12 (d, 1H, *J*_6′′,5′′_ = 9.6 Hz, H-6′′), 8.03 (d, 1H, *J*_3′,4′_ = 7.6 Hz, H-3′), 7.79 (d, 1H, *J*_5,6_ = 7.6 Hz, H-5), 7.65 (t, 1H, *J*_7,(6,8)_ = 7.2 Hz, H-7), 7.47 (d + t, 2H, *J*_6,(5,7)/8,7_ = 7.2 Hz, H-6, H-8), 7.41 (t, 1H, *J*_5′,(4′,6′)_ = 7.6 Hz, H-5′), 7.28 (d, 1H, *J*_6′,5′_ = 8.4 Hz, H-6′), 7.11 (t, 1H, *J*_4′,(3′,5′)_ = 7.6 Hz, H-4′), 5.09 (s, 2H, C*H*_2_-9); EI-MS *m/z* (%): 460 (M^+^, 7), 301 (4), 278 (3), 263 (14), 159 (100), 115 (38), 77 (11); HREI-MS: calcd. for C_23_H_16_O_7_N_4_ (M^+^): *m/z* 460.1019, found: 460.1024.

### Procedure for catalyst recycling experiments

In order to find out the recycling potential of the synthesized ionic liquid catalyst, three experiments of Schiff base formation were performed in three cycles. Each cycle of experiment utilized the recovered catalyst from previously performed experiment; keeping the same experimental conditions each time *i.e*., heating 2 mol% DABCO-*C*_*7*_-F catalyst, coumarin aldehyde (1 equiv), substituted phenylhydrazine hydrochlorides (1.1 equiv) in EtOH at 70–80 °C. After completion of reaction, addition of crushed ice facilitates in precipitation of product. Filtration of product as a residue leaves ionic liquid in the filtrate as aqueous solution. In order to recover ionic liquid from the aqueous solution, lyophilization (*aka* freeze drying) method is used. In this procedure, low temperature dehydration of the solution is done, facilitating in easy removal of water with no damage and full preservation of the material. After freeze drying, the recovered ionic liquid was subjected to ^1^H-NMR spectrum for ensuring the stability of recovered catalyst. Schiff base product obtained after first reaction was 88%. The recovered catalyst was then re-used for the second cycle of experiment in a similar way as mentioned before; which in-turn will recover ionic liquid for the third cycle of experiment. After each cycle, the catalyst was freeze dried* via* lyophilization and the recovered amount of catalyst was again utilized to perform the reactions. The recorded isolated yields of the product for the second and third cycles were 75% and 70%, respectively; which showed a moderate loss in the percentage yields of the products (Schiff base) highlighting the sustainable behavior of ionic liquid.

### Enzyme Inhibition assay for ALR2 (aldose reductase) and ALR1 (aldehyde reductase)

The ALR2 activity was estimated through measuring decrease in absorbance of NADPH in the assay at 340 nm on a spectrophotometer (FLUOstar Omega BMG LABTECH, Germany). The enzyme inhibition assay was carried out *via* following methods of ALR2 inhibition of our recent study^32^. During the assay, total assay volume was kept 100 consisting of 20 *µ*L of 100 mM sodium phosphate buffer at pH 6.2, 30 *µ*L of the enzyme (expressed in the bacterial system; protein concentration 12 *µ*g mL^−1^), 10 *µ*L of test compound, and 20 *µ*L of 1 mM substrate DL-glyceraldehyde. First, the reaction mixture was incubated without co-factor (NADPH) then reaction was initiated by addition of 20 *µ*L of NADPH solution of 0.1 mM and monitored for five minutes. The change of absorbance was noted as pre-read and after-read, the percent inhibition was calculated for the tested compounds and standard inhibitor. For ALR1 (aldehyde reductase), sodium-D-glucuronate was employed as a substrate and valproic acid as a standard inhibitor. While for ALR2, DL-glyceraldehyde was used as the substrate, and sorbinil as the standard inhibitor. Otherwise, identical protocols were adopted for the ALR1 and ALR2 assays. First, tests compounds were solubilized in dimethyl sulfoxide (DMSO) (100%) and dilutions were prepared with deionized water to keep the concentration of DMSO at 0.1% during the assay. Initially, test compounds were tested at 100 *µ*M. The compounds exhibited greater than 50% inhibition, were further analyzed to estimate their IC_50_ values using different dilutions up to 10 nM. The logarithms of the inhibitor concentration were plotted *versus* the remaining activity of the enzyme, and IC_50_ values were calculated using non-linear regression analysis in GraphPad Prism Version 8.

## Results and discussion

### Chemistry

**Fig. 4:**
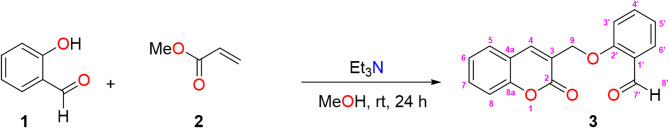
Synthesis of 2*′*-((2-oxo-2*H*-chromen-3-yl)methoxy)benzaldehyde (**3**).

Coumarin-linked Schiff base analogues were synthesized starting from 2′-((2-oxo-2*H*-chromen-3-yl)methoxy)benzaldehyde (**3**) as a precursor molecule. Aldehyde (**3**) was prepared *via* triethylamine-catalyzed condensation of methyl acrylate (**2**) with two equivalences of salicylaldehyde (**1**)^30^, as depicted in Fig. 4.

**Fig. 5:**
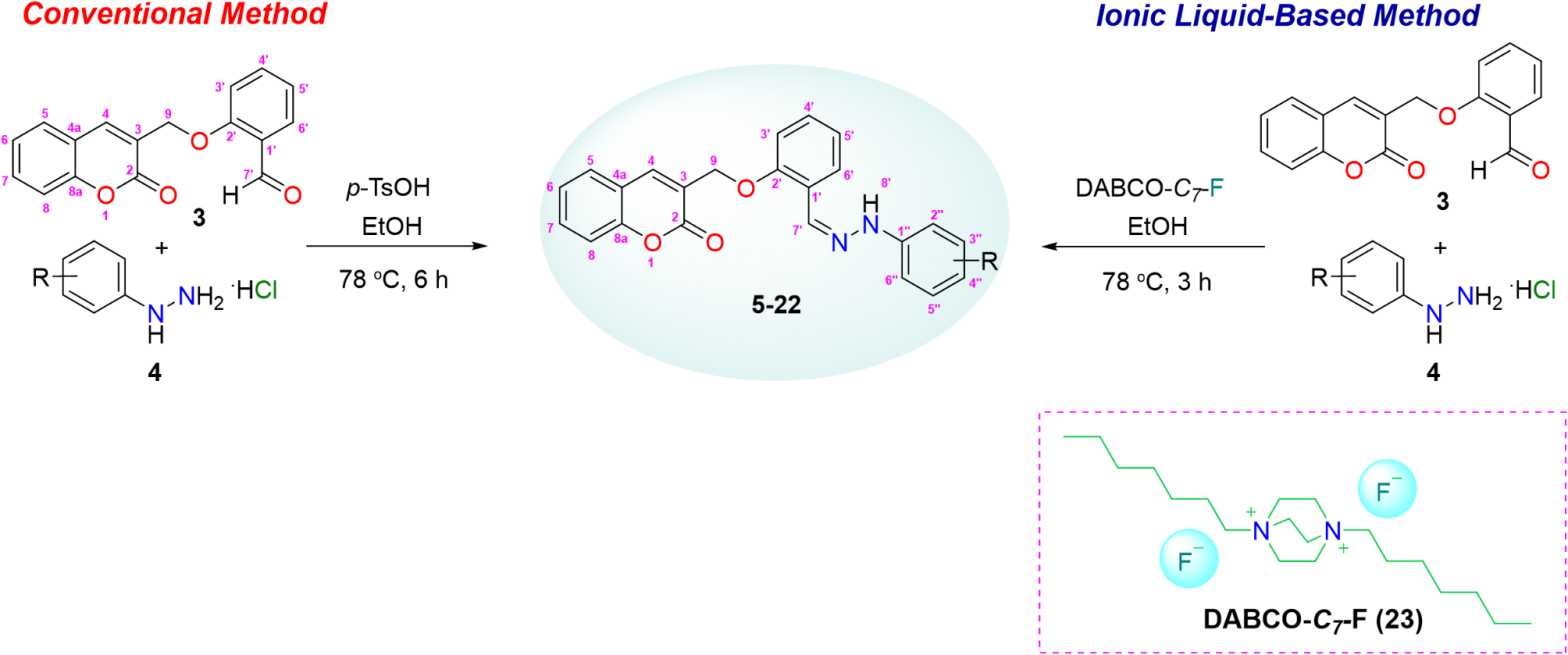
Synthetic scheme for coumarin-linked Schiff bases (**5–22**) using conventional and DABCO-F IL-based methods.

**Fig. 6:**
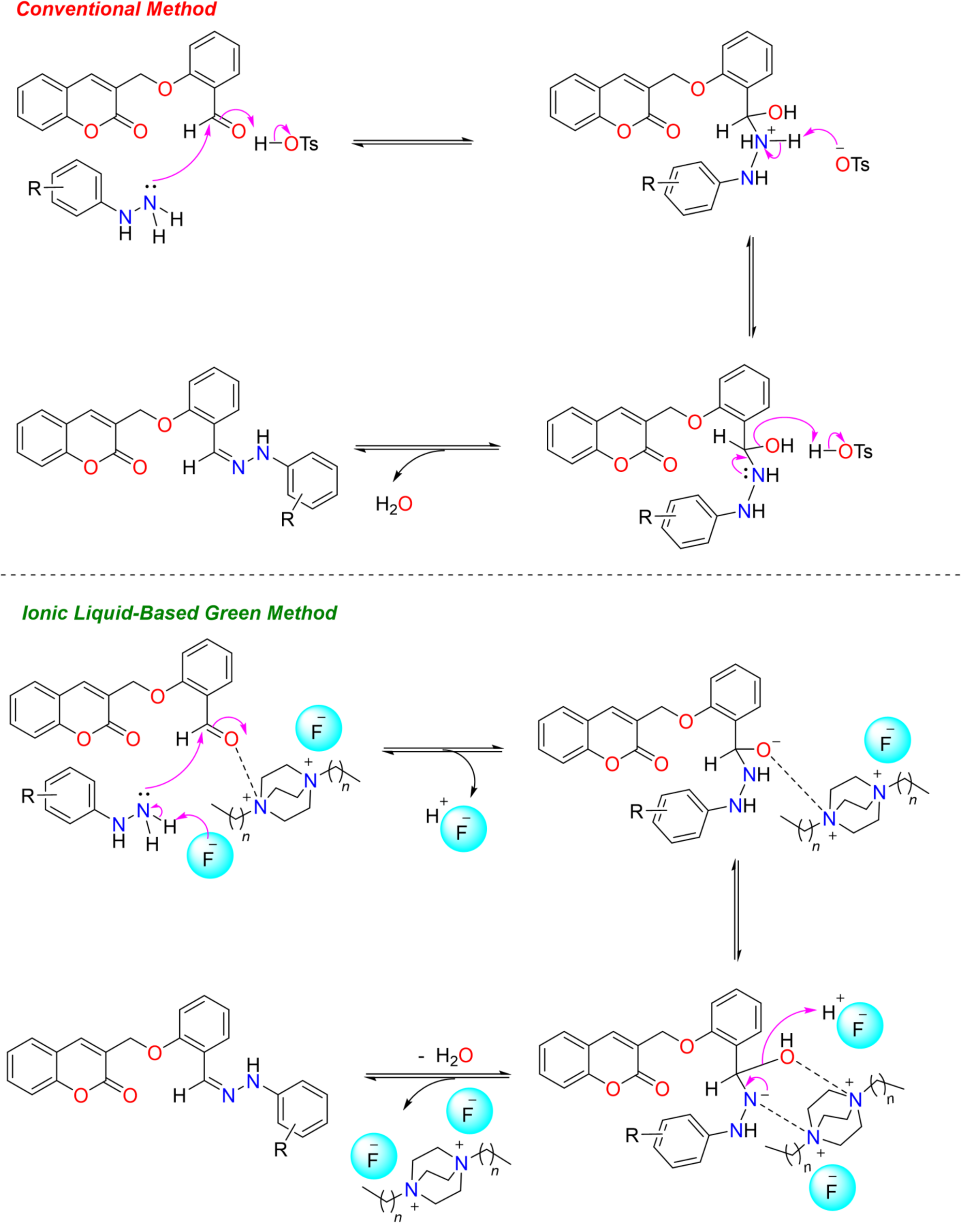
Mechanistic insights for the synthesis of coumarin-linked Schiff bases using DABCO-F IL.

In the next step, the aldehyde (**3**) was treated with different substituted phenyl hydrazine hydrochlorides (**4**) to obtain coumarin-linked Schiff base analogues (**5–22**) as a condensation product using different reaction conditions (Fig. 5). For optimization studies, synthesis of compound **5** was taken as a representative among the series. Initially, reaction was conducted under thermal-catalysis (at 78 °C) without use of any other catalyst. The reaction resulted in 43% of expected product (compound **5**) and was completed in 8 h. Then, reaction was conducted in the presence of 10 mol% *p*-toulenesulfonic acid (*p*-TsOH) as an acid catalyst and heated at same temperature. Reaction was completed in 6 h giving desired coumarin-linked Schiff base (**5**) in 79% yield. Use of *p*-TsOH as an acid source showed remarkable change in % yield, making it a better conventional method for the synthesis of compound **5** (Table 1). Thus, a library of coumarin-linked Schiff base analogues (**5–22**) with varying substituents was prepared in 62–89% yields using *p*-TsOH (10 mol%) as an acid catalyst and ethanol as a solvent at 78 °C. The general mechanism of the conventional reaction utilizing *p*-TsOH for the Schiff base formation, is illustrated in Fig. 6.

**Table 1:** Optimization of conventional and DABCO-F based IL for the synthesis of coumarin-linked schiff base (**5**).

Entry	Catalyst	Catalyst loading (mol%)	Time (h)	Temp (°C)	% Yield
1	None	–	8	78	43
2	*p*-TsOH	10	6	78	79
3	DABCO-*C*_*7*_-F	0.5	3	78	56
4	DABCO-*C*_*7*_-F	1	3	78	65
5	DABCO-C_7_-F	2	3	78	85
6	DABCO-*C*_*7*_-F	5	3	78	84

**Fig. 7:**
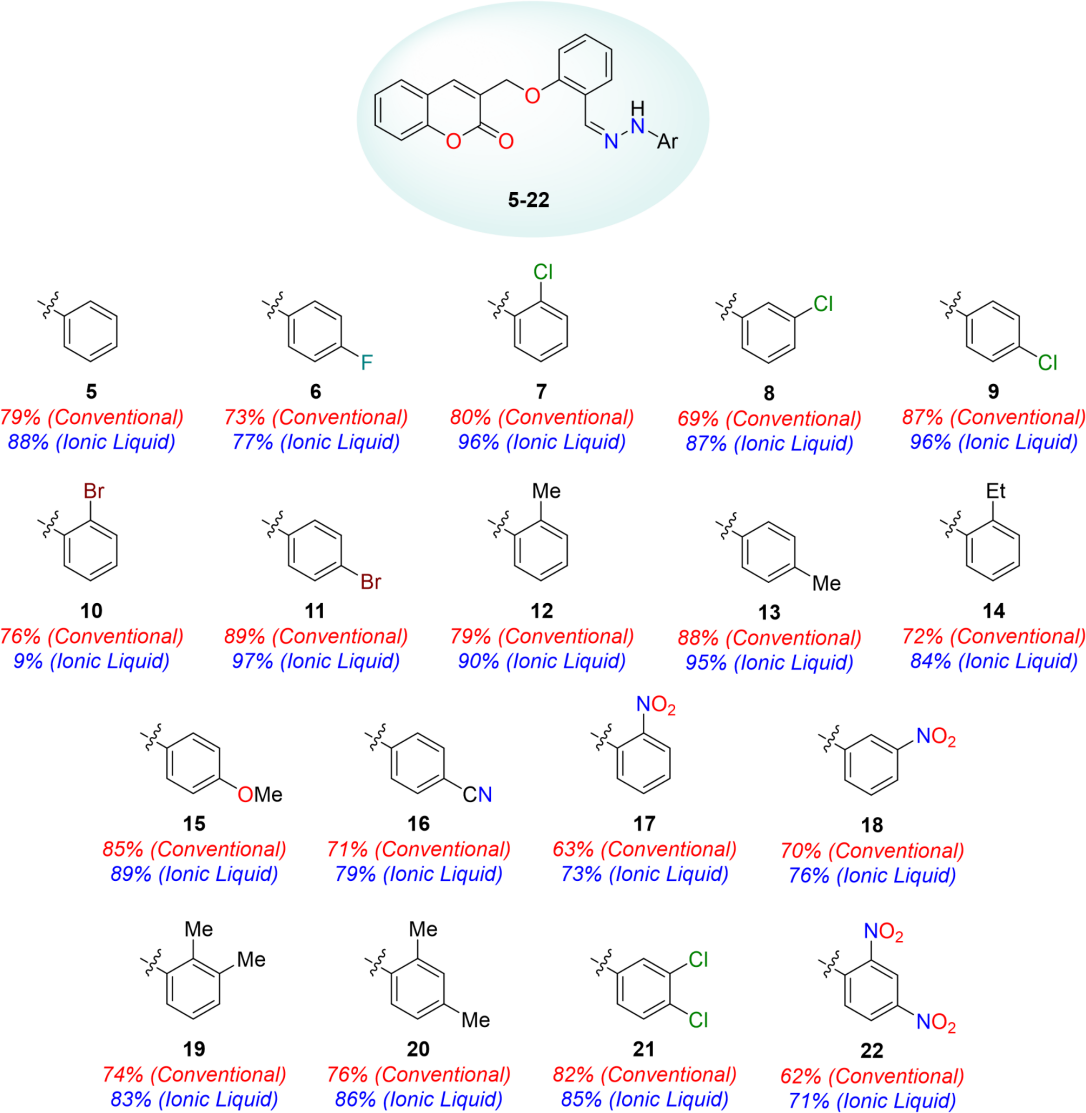
Library of coumarin-linked Schiff bases (**5–22**).

All the described yields for coumarin-linked Schiff base analogues (**5–22**) are summarized in the Fig. 7. Unsubstituted analogue (**5**) showed promising yield *i.e*., 79%. It was observed that excellent yields (74–88%) were obtained for compounds **12**, **13**, **15**, **19**, and **20** with electron-donating groups such as methyl, ethyl and methoxy. However, ethyl substituted analogue (**14**) was obtained in relatively lesser yield (72%) amongst them, owing to its steric crowding in the molecule. Compounds **16**–**18** and **22** with electron-withdrawing groups slightly lower the yields (62–71%) as compared to the compounds with electron-donating groups; highlighting that presence of electron-donating groups in precursor nucleophilic molecule (phenyl hydrazines) increases the productivity. In addition, 2′′- and 4′′-halogenated analogues (**6, 7** and **9–11**) offered better yields as compared to 3′′-halogenated analogue (**8**) (69% yield). Also, 4′′-chloro- (**9**) and 4′′-bromo-substituted analogues (**11**) were obtained in better yields as compared to 4′′-fluoro-substituted analogue (**6**), as fluorine atom decreases nucleophilicity of the corresponding phenyl hydrazine.

In our previous report^24^, new DABCO-*C*_*7*_-F ionic liquid (IL) (**23**) was reported as eco-friendly and efficient catalytic medium for the syntheses of Fischer-indoles, and multi-component products; and was prepared in a two-step reaction sequence, including quaternatization reaction of DABCO with *n*‒heptyl bromide followed by anionic exchange using silver fluoride (AgF). To explore new reaction application of IL (**23**), coumarin-linked Schiff base analogues (**5–22**) were prepared in the presence of DABCO-*C*_*7*_-F IL (**23**) utilizing a sustainable or green method. For this, initial optimization studies were conducted to search optimum catalyst loading for the aforementioned reaction (Table 1), while keeping all other reaction parameters fixed for rest of the studies. Coumarin-linked Schiff base **5** was taken as a representative compound. At 0.5 mol% of DABCO-*C*_*7*_-F IL catalyst, 56% of the desired product (compound **5**) was obtained, while 1 mol% of IL resulted in 65% yield of compound **5**. Further, comparative study showed 85% yield of the product (compound **5**) was obtained when 2 mol% of the IL was used. Comparison of reaction being catalyzed at 5 mol% of the IL showed 84% yield for the product. This sets the optimum catalyst loading of DABCO-*C*_*7*_-F IL (**23**) for Schiff base formation at 2 mol%. Thus, a library of coumarin-linked Schiff base analogues (**5–22**) was re-prepared using DABCO-*C*_*7*_-F IL in ethanol at 78 °C in 3 h (Fig. 7) and compared for its efficacy. Proposed mechanism of the Schiff base synthesis utilizing IL is described in Fig. 6. The structure of synthesized IL showed that it is comprised of DABCO-*C*_7_ di-cations and two flouride anions. The ionic catalyst has advantage over conventional ionic liquids as it has dual nature as catalyst and medium for the reaction and can be useful for strong H-bonding with the substrate. Proposed mechanism of the Schiff base synthesis utilizing IL is described in Fig. 6. In this mechanism, the electrophilicity of the carbonyl carbon of the aldehyde might be increased with the electrostatic interaction between the cation of the DABCO-*C*_7_ cation and oxygen atom of the carbonyl group of the aldehyde, while the nucleophilicity of the hydrazine might be increased by form H-bonding between the H-atom of the hydrazine and fluoride of the ionic liquid; thus enhancing the speed of the reaction that leads to the formation of intermediate. The intermediate is then followed by condensation reaction and give the corresponding Schiff base while the DABCO-*C*_7_-F catalyst is recovered.

Unsubstituted analogue (**5**) was prepared in 85% yield. Among 4′′-substituted halogenated analogues (**6, 9** and **11**), excellent yields were obtained with a decreasing trend moving from bromo- to fluoro-substituted analogue. 97% Yield was acquired for bromo-substituted analogue (**11**), 93% chloro-substituted (**9**) and 79% yield for *p*-flouro analogue (**6**). 2′′-Substituted halogenated compounds (**7** and **10**) showed relatively better results (> 90% yields) as compared to compound **8** with chloro-substitutent at 3′′-position, which gave 87% yield. Further comparison of 2′′-chloro-substituted compound (**7**) (90% yield) with 2,6-dichloro-substituted compound (**21**) (85% yield) revealed a decrease in the activity for disubstitution. Monosubstituted compounds with electron-donating groups such as methyl, ethyl and methoxy (compounds **12–15**) also showed comparatively better yields (89–95%) than compound **14** (2′′-ethyl group) (84% yield). Reason for lowering of yield might be steric hindrance offered by ethyl group. Disubstituted compounds **19** and **20** with two methyl groups at 2′′,3′′- and 2′′,4′′-positions, respectively, showed similar results *i.e*., *≥* 83% yield. On the contrary, compounds **16**, **17**, **18** and **22** with electron-withdrawing substituents such as nitro and cyano groups, showed lowering in yields ranging from 71 to 79%. Reason for lowering of yield might be as a result of decrease in nucleophilicity of precursor phenyl hydrazines due to presence of electron-withdrawing groups. Evidence for aforementioned reason is that replacing nitro with cyano group decreased the yield relatively and presence of two nitro groups further decreased the yield.

Results obtained* via* comparing two methods* i.e*., sustainable or green method using DABCO-*C*_*7*_-F IL and conventional method using *p*-TsOH, revealed better yields for DABCO-*C*_*7*_-F IL as a catalyst. Same trend observed for halogenated, electron-donating, and electron-withdrawing groups was on the yields for both methods. Further, reaction took 3 h for completion using DABCO-*C*_*7*_-F IL while conventional method requires 6 h. Both reactions were conducted at 78 °C.

**Fig. 8:**
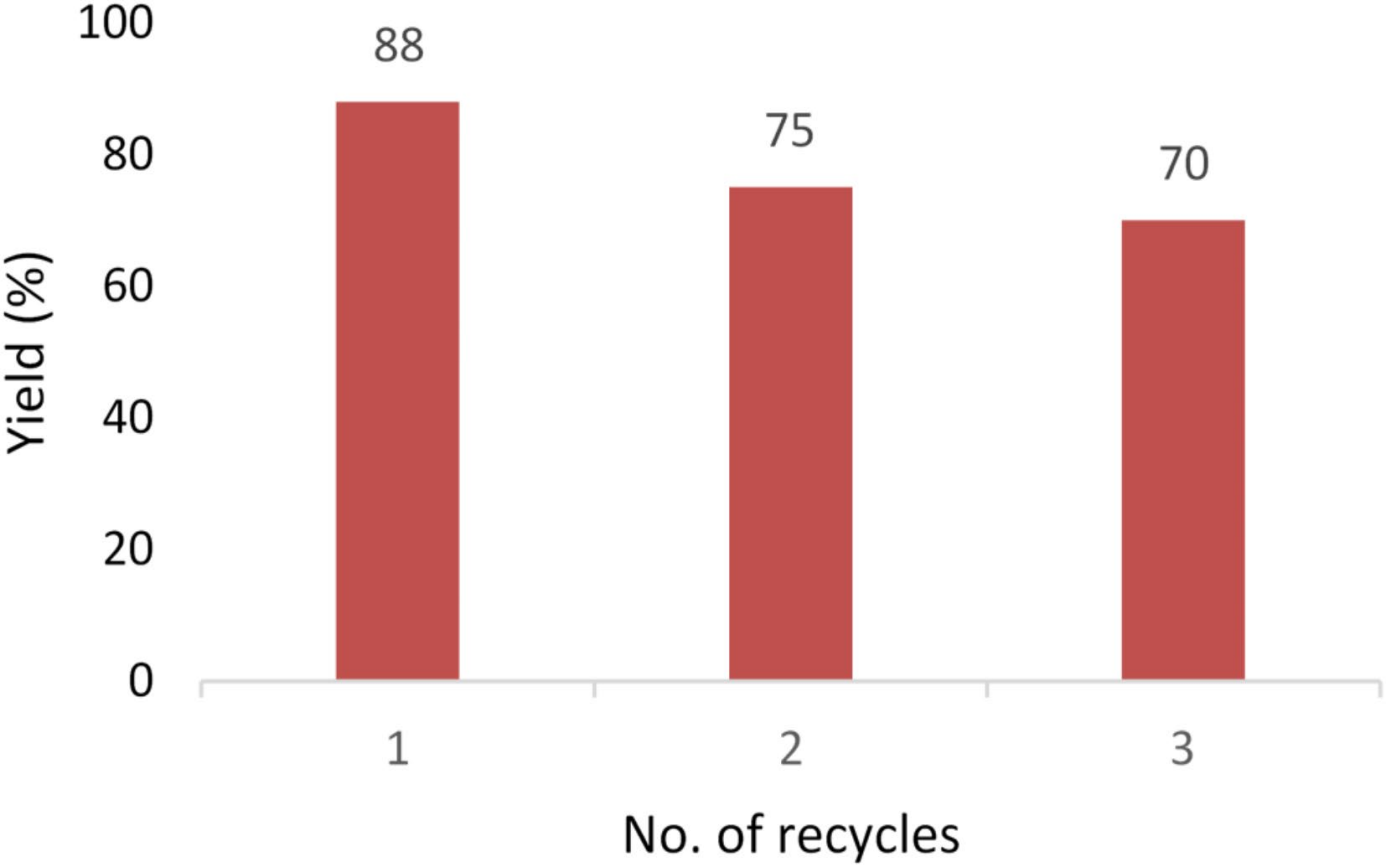
Recycling data of the catalyst.

The recycling experiments were also performed to explore the recyclability level of the synthesized DABCO-*C*_7_–F catalyst, as illustrated in Fig. 8. The recovered ionic liquid catalyzed the formation of Schiff base (**5**) successfully for three consecutive cycles. The model reaction was setup *via* heating all the starting materials together using 2 mol% catalyst loading of DABCO-*C*_7_–F IL, and was conducted at 78 °C for 3 h to afford precipitates of the product. Filtrate was freeze-dried, and then re-used for the next reaction providing good results. Yield obtained from the first reaction was 88%; second recycling reaction gave 75%, while third recovered catalyst gave 70% yield. As catalyst efficiency is still better even after recycling therefore, it was concluded that the catalyst is sustainable in nature.

**Fig. 9:**
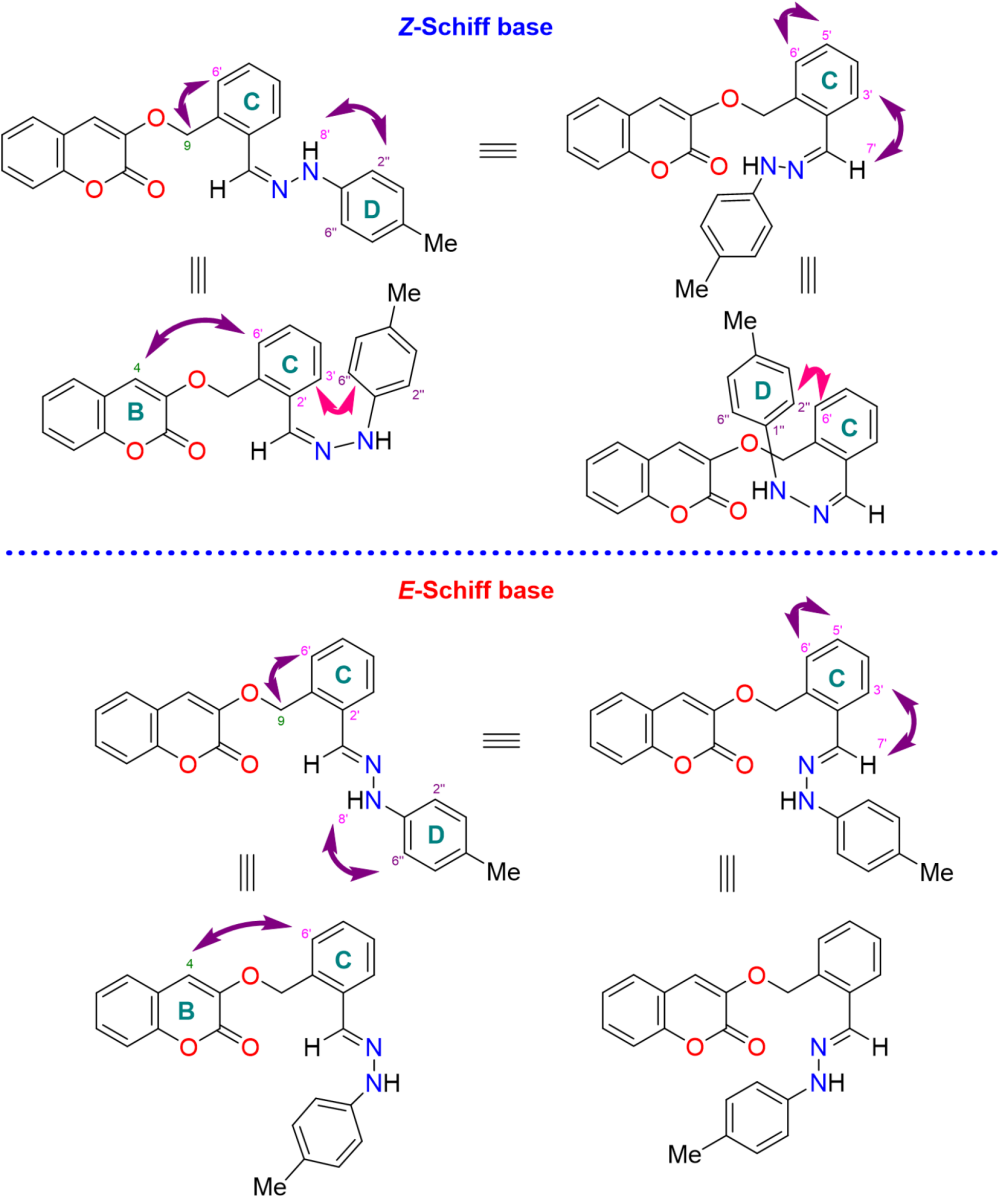
Possible NOE interactions among different conformations of *Z*- and *E*-configuration of compound **13**.

Synthesized compounds were characterized using spectroscopic studies. Coumarin-Schiff base (**13**) was taken as a representative for the explanation of spectral data. Compound **13** was obtained as an off-white solid with a melting point (m.p.) of 175–178 °C, which showed the molecular ion peak [M^+^] at *m/z* 384.1475 in the HR EI-MS spectrum for the molecular formula C_24_H_20_O_3_N_2_. IR showed peaks for the N‒H stretch at 3426, C=O stretch at 1724, C=C stretch at 1520, and C‒O bend at 1173 cm^−1^; while UV showed absorptions at 358, and 269 nm. The ^13^C-NMR exhibited one methyl (CH_3_), one methylene (CH_2_), twelve methine (CH), and eight quaternary carbon signals; which confirmed the skeleton of desired compounds (Table 2). Formation of Schiff base was confirmed by disappearance of aldehydic proton at *δ* 10.51; and appearance of characteristic imine proton (H-7′) at *δ* 8.21 as a singlet (along with amine proton at *δ* 10.21 as a broad singlet, aromatic protons between *δ* 6.93 as a doublet, and *δ* 7.02–6.99 as an overlapped multiplet; which were identified as a part of same spin system on the basis of TOCSY and COSY correlations). Imine proton corresponds to *δ* 131.2 in HSQC experiment, and was identified as C-7′. Amino proton (H-8′) showed NOE correlations with one of the aromatic protons of aniline part* i.e*., *δ* 6.93, which aids in its assignment as H-2′′/H-6′′; leaving multiplet at *δ* 7.02–6.99 to be identified as H-3′′/H-5′′. Assignment of H-3′′/H-5′′ is further supported by their NOESY correlation with methyl protons (CH_3_-3′′). H-2′′/H-6′′, H-3′′/H-5′′, and CH_3_-3′′ showed interactions in HSQC experiment with *δ* 111.9 (C-2′′/C-6′′), 129.5 (C-3′′/C-5′′), and 20.2 (C-3′′), respectively. The singlet for imine proton (H-7′) at *δ* 8.21 was segregated from singlet for aromatic proton (H-4) of coumarin skeleton (ring **B**) at *δ* 8.19 on the basis of their HMBC correlations. H-7′ correlates with aromatic proton of ring C (H-3′), while H-4 correlates with methylene protons (CH_2_-9) and other aromatic proton of ring **A** (H-5). H-4 showed interaction with *δ* 140.5 in HSQC experiment and was identified as C-4. Identification of H-5 facilitates in the identification of rest of the aromatic protons of ring **A**. H-5 showed interaction with a triplet of doublet at *δ* 7.39 in COSY experiment, and was found to be H-6; which in turn showed COSY correlation with a triplet of doublet at *δ* 7.64 and was identified as H-7. Aromatic proton (H-7) then showed interactions in COSY experiment with other aromatic proton resonating at *δ* 7.46, and permitting it to be assigned as H-8. NOESY and HMBC correlations further support the assignments of aromatic protons of ring **A**. H-5, H-6, H-7, and H-8 showed interactions with *δ* 128.6 (C-5), 124.6 (C-6), 132.0 (C-7), and 116.2 (C-8) in HSQC experiment, respectively. As mentioned above, imine proton (H-7′) correlates with aromatic proton of ring **C** (H-3′) in HMBC experiment; it will aid in the assignment of other aromatic protons of ring **C**. TOCSY experiment showed aromatic protons at *δ* 7.02–6.99 (overlap signal as a multiplet), 7.15 (doublet), 7.25 (triplet of doublet) and 7.87 (doublet of doublet) as a part of one spin system; amongst which *δ* 7.87 (doublet of doublet) was already identified as H-3′. This left the other doublet at *δ* 7.15 to be identified as H-6′. Aromatic proton (H-3′) showed COSY interaction with a multiplet at *δ* 7.02–6.99, permitting it to be assigned as H-4′; which further correlates in COSY experiment with a triplet of doublet at *δ* 7.25, and is found to be H-5′. Aromatic proton (H-5′) showed COSY interactions with a doublet at *δ* 7.15, and confirmed it as H-6′. Also, NOESY and HMBC correlations further support the assignments of aromatic protons of ring **B**. Assignment of quaternary carbons was done on the basis of correlations observed in the HMBC experiment. Characteristic carbonyl carbon (C-2) appeared at *δ* 159.6 and showed a correlation with methylene protons at CH_2_-9, and aromatic proton at ring **C** (H-4). While, quaternary carbon appearing at *δ* 124.1 was identified as C-3. Among other quaternary carbons of coumarin skeleton, C-8a (resonating at *δ* 152.9) was found lowfield due to resonance effect and correlated with aromatic protons of coumarin skeleton (H-4, H-5, H-6, H-7, and H-8). In contrast, quaternary carbons C-4a (resonating at *δ* 118.8) showed HMBC interactions with aromatic protons, H-4, H-6, and H-8. Quaternary carbon at *δ* 124.5 and 155.1 showed common HMBC interactions with aromatic proton of ring **C** (H-5′ and H-6′), and methylene (CH_2_-9), which identified them as C-1′ or C-2′. HMBC correlations of carbon at *δ* 155.1 with aromatic methines (H-3′ and H-7′) assigned it as C-1′. In comparison, quaternary carbon appearing at *δ* 124.5, showed correlations with aromatic protons (H-4′) and was identified as C-2′. Quaternary carbons at *δ* 127.1 and 143.1 were identified as C-1′′ or C-4′′ on the basis of their HMBC interactions with aromatic protons (H-2′′/H-6′′ and H-3′′/H-5′′) of ring **D**, and were further differentiated on the basis of coupling of carbon appearing at *δ* 143.1 with methyl protons (CH_3_-7′′). This lead to identification of carbon appearing at *δ* 143.1 as C-4′′; leaving carbons at *δ* 127.1 to be identified as C-1′′. In the NOESY experiment, several correlations are observed such as, between amine proton (*N*H-8) and aromatic protons of ring **D** (H-2′′/H-6′′); between imine proton (H-7′) with aromatic protons of ring **C** (H-3′); and interaction of aromatic proton (H-6′) of ring **C** with CH_2_-9, H-4 and H-5′. These interactions support the assignment done for protons of compound **13** as explained above. However, these interactions are common among various below-mentioned conformers of both *E*- and *Z*-isomers. However, the NOESY correlation between aromatic protons (H-2′′/H-6′′) of ring **D** and proton (H-3′) of ring **C**, indicated that these protons are in near-by vicinity; which is only possible in one of the conformation of *Z*-isomer. Cross peaks of aromatic protons (H-2′′/H-6′′) of ring **D** with aromatic proton of ring **C** connected to C-6′ in NOESY experiment, further confirmed its configuration as *Z* (Fig. 9).

**Table 2:** ^1^H-NMR, ^13^C-NMR and 2D-NMR data analyses of methyl-substituted schiff base (**13**).

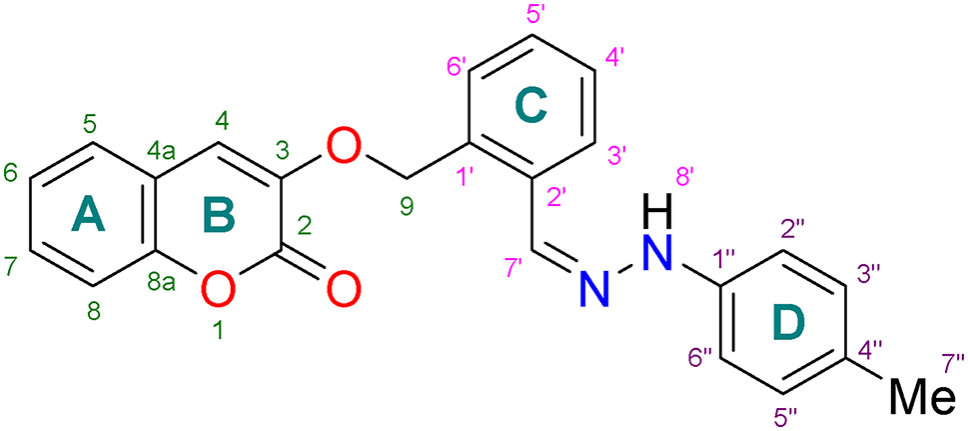							
C #	*δ*_C_	*δ*_H_	Mult.	HBMC	TOCSY	COSY	NOESY
**2**	159.6	-	C	H-4, CH_2_-9	–	–	–
**1′**	155.1	-	C	H-3′, H-5′, H-6′, H-7′, CH_2_-9	–	–	–
**8a**	152.9	–	C	H-4, H-5, H-6, H-7, H-8	–	–	–
**4′′**	143.1	-	C	H-3′′/H-5′′, H-2′′/H-6′′, CH_3_-7′′	–	–	–
**4**	140.5	8.19 (s)	CH	H-5, CH_2_-9	–	–	–
**7**	132.0	7.64 (td, 9.0, 4.0)	CH	H-5	H-5	–	–
**7′**	131.2	8.21 (s)	CH	H-3′	NH-8′	–	–
**3′′/5′′**	129.5	7.02–6.99 (m) (*overlap*)	CH	H-3′′/H-5′′	H-2′′/H-6′′	–	–
**5′**	128.9	7.25 (td, 12.0, 2.0)	CH	H-3′, H-6′	H-3′	–	H-6′
**5**	128.6	7.79 (dd, 8.0, 1.4)	CH	H-4, H-7	–	–	H-4
**1′′**	127.1	-	C	H-2′′/H-6′′	–	–	–
**3′**	124.8	7.87 (dd, 8.0, 1.7)	CH	–	–	–	H-7′
**6**	124.6	7.39 (td, 8.0, 1.3)	CH	H-8	H-5, H-7	H-5, H-7	H-5, H-7
**2′**	124.5	–	C	H-4′, H-5′, H-6′, CH_2_-9	–	–	–
**3**	124.1	–	C	–	–	-	–
**4′**	121.3	7.02–6.99 (m) (*overlap*)	CH	H-6′	H-3′, H-5′, H-6′	H-3′, H-5′	H-3′, H-5′, H-6′
**4a**	118.8	–	C	H-4, H-6, H-8	–	–	–
**8**	116.2	7.46 (d, 8.0)	CH	H-6	H-5, H-7	H-7	H-7
**6′**	112.9	7.15 (d, 8.0)	CH	H-4′	H-3′, H-5′	H-5′	H-4, H-5′
**2′′/6′′**	111.9	6.93 (d, 8.0)	CH	H-2′′/H-6′′, CH_3_-7′′, NH-8′	H-3′′/H-5′′	–	H-3′, H-6′, NH-8′
**9**	65.3	5.03 (s)	CH_2_	H-4	H-4, H-5, H-6, H-7, H-8, H-3′, H-4′, H-5′, H-6′, H-7′	–	H-6′
**7′′**	20.2	2.19 (s)	CH_3_	H-2′′/H-6′′, H-3′′/H-5′′	H-2′′/H-6′′, H-3′′/H-5′′,	–	H-3′′/H-5′′, NH-8′
**8′**	–	10.21 (s)	NH	–	–	–	–

### Aldose reductase inhibitory activity

A number of coumarin analogues have been reported as potent and selective inhibitors of ALR2 enzyme. Similarly, compounds bearing Schiff base moiety also presented significant ALR inhibitory activities. Based on these reports, a library of coumarin-linked Schiff base analogues were designed. Synthesized coumarin-based Schiff base analogues (**5–22**) were tested against aldose reductase (ALR2) enzyme to evaluate their inhibitory potential for identifying new anti-diabetic molecules. Compounds were also checked for selectivity *via* testing their inhibitory potency against ALR1 enzyme. Both aldose reductase enzymes (ALR2 and ALR1) were isolated from bovine; ALR2 was extracted from the eyes while ALR1 was obtained from the kidneys. Sorbinil was used as a standard for ALR2 with IC_50_ value of 3.14 ± 0.02 *µ*M, while valproic acid was taken as a reference inhibitor for ALR1 with IC_50_ value of 57.4 ± 0.89 *µ*M (Table 3).

Results revealed that coumarin-based Schiff bases (**5–9, 12–15**, and **19–21**) showed more than 50% inhibition against ALR2 enzyme, and demonstrated significant activity against the target enzyme ALR2 with their IC_50_ values ranging between 1.61 and 11.20 *µ*M. These activity results are comparable to that of the reported coumarin^18–20^, and Schiff base analogues in the literature, as mentioned before^22,23^. Among them, compounds **6**, **8**, **9**, and **19** showed strong inhibitory potential; compounds **7**, **12**, **13**, and **21** exhibited activities comparable to the reference; while compounds **5**, **14**, **15**, and **20** demonstrated activities weaker than that of the standard inhibitor, sorbinil. Comparison among inhibitory activity against both enzymes (ALR2 and ALR1) revealed that compounds **5**, **6**, **12**, **13**, and **19** exhibited selective inhibition against ALR2 enzyme; while compounds **7**–**9**, **14**, **15**, **20**, and **21** showed non-selective inhibitions. On the other hand, rest of compounds **10**, **11**, **16**–**18**, and **22** exhibited less than 50% inhibition for both ALR2 and ALR1 enzymes; hence were found to be inactive (Table 3).

Unsubstituted analogue (**5**) demonstrated selective inhibition against ALR2 with IC_50_ value of 11.20 ± 0.029 *µ*M, and is ~ 4-times weaker than the standard, sorbinil (IC_50_ = 3.14 ± 0.02 *µ*M). Structure-activity relationship (SAR) revealed that addition of mono- or di-substitutions on phenyl ring not only increases the activity with IC_50_ values ranging between 1.61 and 10.19 *µ*M, but also changes their selectivity pattern towards ALR2 inhibition.

Amongst monosubstituted halogenated compounds, all bromo-substituted analogues (**10** and **11**) were found inactive; however, fluoro- (**6**) and chloro-substituted analogues (**9–9**) showed comparable or slightly better activity than the standard. Comparison between compounds with halogen-substitutions at 4′′-position revealed that fluoro-substituted analogue (**6**) (IC_50_ = 2.20 ± 0.024 *µ*M) is the most active compound and is better in activity than the standard. Compound **6** is also more active than chloro-substituted compound **9** (IC_50_ = 2.84 ± 0.032 *µ*M), which is in turn more active than bromo-substituted analogue (**11**) as no inhibition was observed for compound **11**. Among chloro-substituted congeners (compounds **7**–**9**), activity is slightly increased as substitution is moved from 2′′- to 4′′-position with IC_50_ values of 3.61 ± 0.016, 2.94 ± 0.083, and 2.84 ± 0.032 *µ*M, respectively. It was observed for 3′′-halogenated analogues that chloro-substituted congener (compound **8**) (IC_50_ = 2.94 ± 0.083 *µ*M) showed slightly better inhibition than the standard, while activity is completely diminished in case of bromo-substituted analogue (**10**). Comparison of mono-chloro-substituted analogue (**7**) (having a chlorine substituent at 2′′-position or at one *ortho*-position) (IC_50_ = 3.61 ± 0.016 *µ*M) with di-chloro-substituted analogue (**21**) (having two chlorine substituents at 3′′,4′′-positions) (IC_50_ = 4.07 ± 0.083 *µ*M) showed a slight increase in the activity for mono-substituted compound with no change in the selectivity. In addition, among all halogenated coumarin-based Schiff base analogues only fluoro-substituted analogue (**6**) showed selective inhibition against ALR2 enzyme.

Presence of electron donating groups such as methyl and methoxy groups as mono- or di-substitutions, possess strong effects on the inhibitory potency with IC_50_ values ranging between 1.6 and 10.19 *µ*M. Amongst methyl-substituted analogues, 2′′-methyl substituted compound **12** (IC_50_ = 3.66 ± 0.068 *µ*M) is found more potent than 4′′-methyl substituted congener (compound **13**) (IC_50_ = 4.64 ± 0.045 *µ*M) with selectivity observed for both analogues. Replacing substituent from methyl to ethyl group (compound **14**) (IC_50_ = 6.52 ± 0.077 *µ*M) results in decrease in activity up to 2-times with the loss of selectivity. Further, changing methyl substituent with methoxy group at 4′′-position also decreases activity ~ 2-times for compound **15** (IC_50_ = 10.19 ± 0.034 *µ*M) with the loss of selectivity towards ALR2 enzyme. Di-substituted compound **19** with the two methyl substituents at 2′′,3′′-positions showed strong inhibition against ALR2 enzyme with IC_50_ value of 1.61 ± 0.049 µM, and is the most active analogue among the series with 2-times better activity than standard, sorbinil (IC_50_ = 3.14 ± 0.02 *µ*M). Comparison of compound **19** with the other dimethylated isomeric compound **20** showed that switching position of one methyl (compound **20**) from position 3′′- to 4′′- (while keeping other methyl fixed at position-2′′), showed strong impact on the activity. This switching showed reduction of the activity up to 4-times (IC_50_ = 7.63 ± 0.061 *µ*M) for compound **20** as well as loss of selectivity for ALR2, as it also exhibited inhibition against ALR1 enzyme with IC_50_ value of 3.29 ± 0.064 *µ*M. In contrast, mono-methylated compounds **12** and **13** with methyl groups at 2′′- and 4′′-positions, respectively showed selective inhibitory potencies, better than 2′′,4′′-dimethylated compound **20** and less than 2′′,3′′-dimethylated compound **19**. Furthermore, among all the compounds with electron donating groups, only mono-methylated analogues (compounds **12** and **13**), and di-methylated analogue (**19**) showed selective inhibition against ALR2 enzyme. Exchanging substituents with electron withdrawing groups, such as cyano or nitro groups resulted in complete loss of activity for compounds **16**–**18** and **22** as exhibited inhibition less than 50%. Also, synthesized compound when screened for their cytotoxicity using human normal BJ cell line, and were found non-cytotoxic.

**Table 3:**
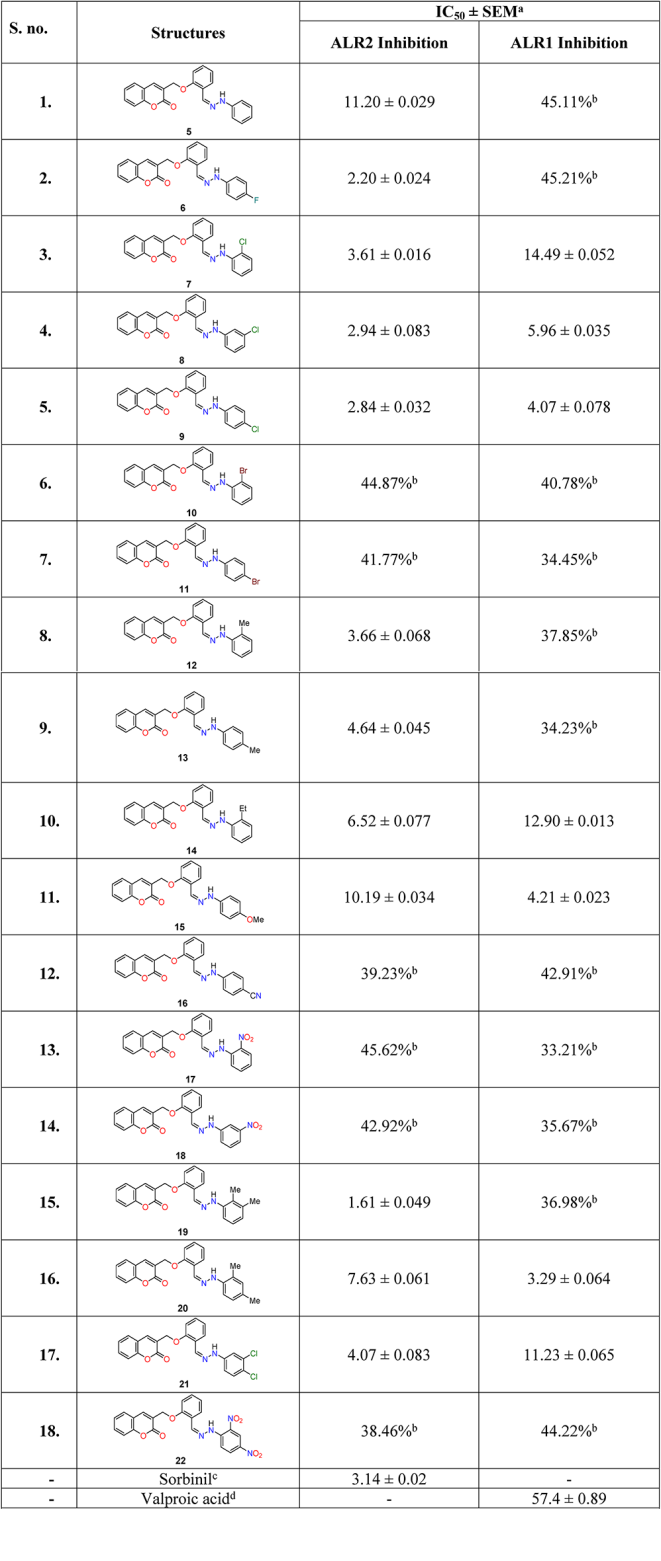
ALR2 and ALR1 Inhibition. of coumarin-linked Schiff bases (**5–22**).

^a^ standard mean error; ^b^ % Inhibition; ^c^ standard for ALR2 Inhibition; ^d^ standard for ALR1 Inhibition.

### Homology modeling and molecular Docking studies

The crystal structure of human aldose reductase (ALR2) was downloaded from the Protein Data Bank (PDB id: 1us0 at 0.66 Å), and used for the docking studies of ALR2 inhibitors. The only available crystal structure of a human aldehyde reductase (PDB id: 2alr) does not contain the important co-factor NADPH, hence a homology model was built. The amino acid sequence of hALR1 was fetched in Chimera^33^ *via* its Uniprot number (P14550). BLAST protein search was carried out to find suitable template for homology modeling. Crystal structure of porcine alcohol dehydrogenase (PDB id: 3fx4) having 94% sequence identity with the target protein was selected to be used as a template. The NADPH co-factor and the co-crystallized inhibitor from the template protein were retained in the homology building. Sequence alignment was using Needleman Wunch logarithm as embedded in Chimera. Modeller was accessed *via* Chimera for generation of the required homology model; a total of 5 models were generated, out of which the best scoring one was selected for further studies. Before docking analysis, the heteroatoms were removed, hydrogen atoms and charges were added and energy was minimized using Chimera. Structure validation was carried out using Molprobity^34^. The Ramachandran plot indicated that 98.5% residues were in favored region, and 100% residues were in allowed region confirming the reliability of the homology built model.

**Fig. 10:**
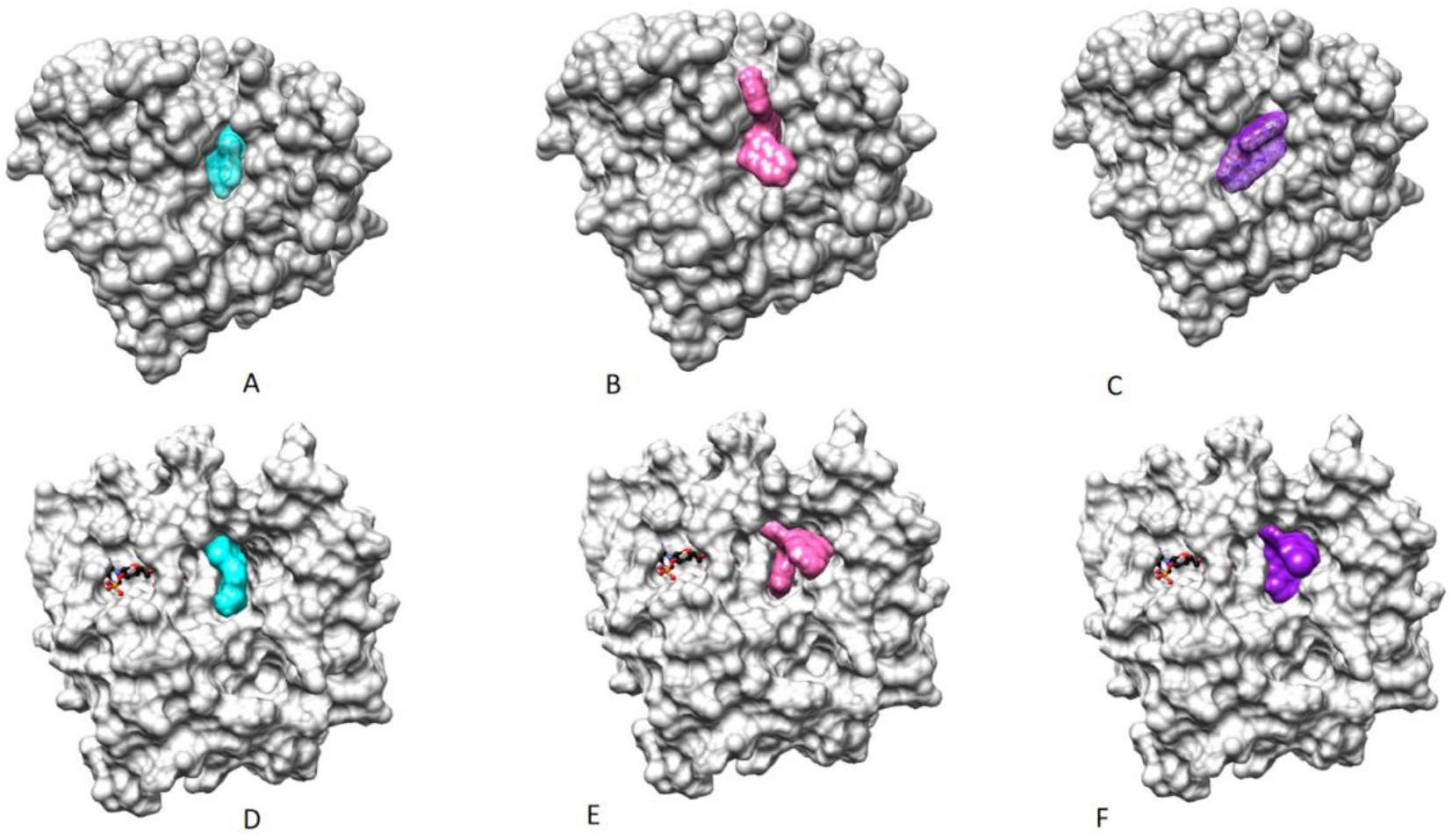
A) Co-crystallized ligand (cyan) bound in the active site of hALR2; docked conformations of ALR2 inhibitors compound **19** (B, pink) and **6** (C, purple) bound in the same area of the active site as the co-crystallized inhibitor. D) Co-crystallized ligand (cyan) bound in the active site of hALR1; docked conformations of ALR1 inhibitors compound **20** (E, pink) and **9** (F, purple) bound in the same area of the active site as the co-crystallized inhibitor, the cofactor NADPH can be seen in black color.

For molecular docking studies, BioSolveIT’sleadIT software^35^ was used. Two most active inhibitors of ALR2 (**19** and **6**) and ALR1 (**20** and **9**) were selected for the docking studies. For each compound, a total of ten docking conformations were generated; each conformation was analyzed using HYDE utility of LeadIT software. The HYDE score is a measure of stability of the ligand interactions inside the binding site. The docked conformation with most favorable HYDE score was selected for the detailed analysis. All compounds were found to bind in the same area of the active site as the co-crystallized inhibitor (Fig. 10).

### Molecular docking of hALR2 inhibitors

**Fig. 11:**
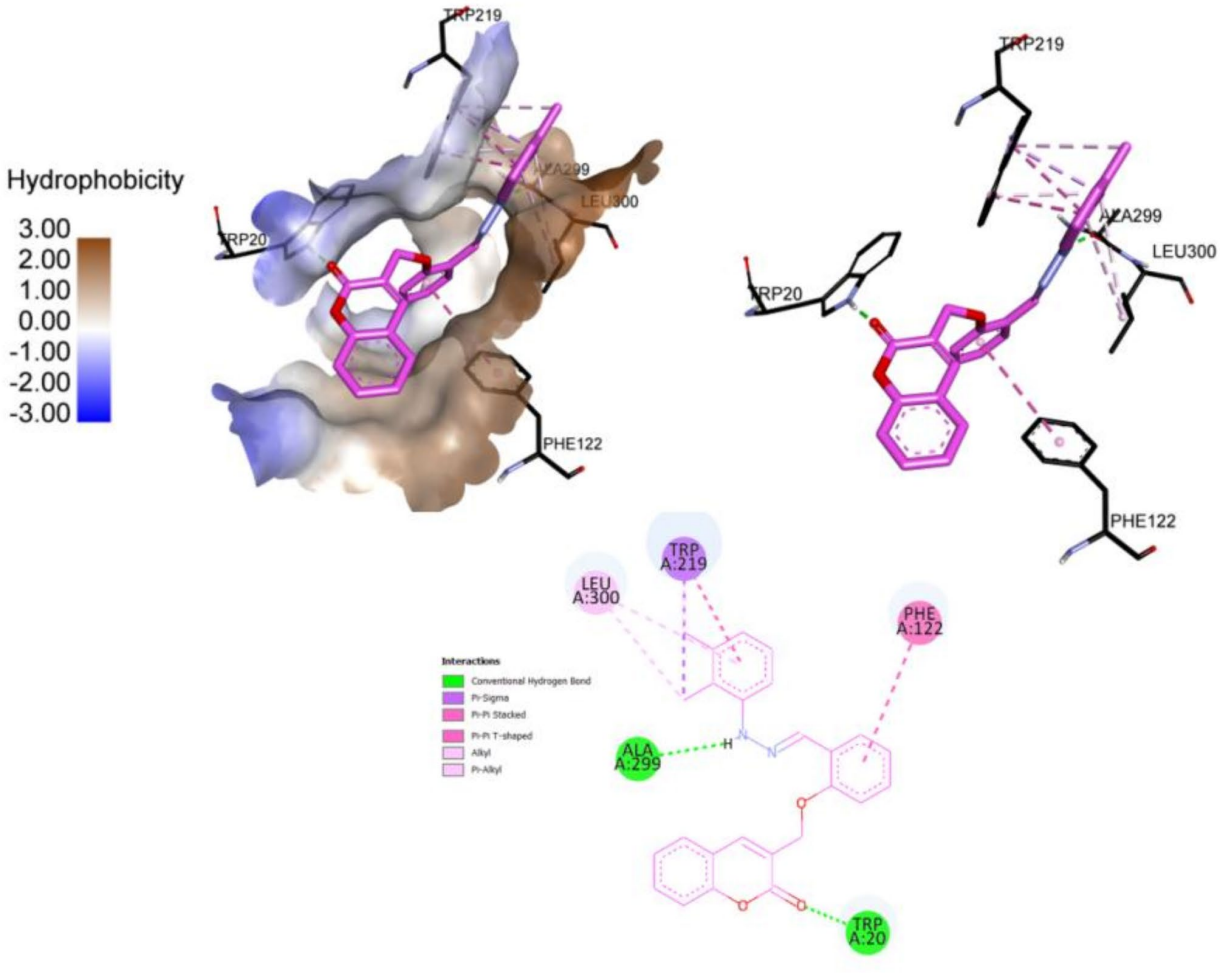
Binding site interactions of ALR2 inhibitor compound **19**.

Compound **19** was the most active ALR2 inhibitor. The docking analysis indicated hydrogen bond formation between the carbonyl oxygen atom and Trp20. Another hydrogen bond was observed between the *N*H group of compound **19** and Ala299. A number of hydrophobic interactions were also observed; one of the methyl groups substituted on the phenyl ring was making a pi-sigma and a pi-alkyl bond with Trp219 and an alkyl bond with Leu300. The other methyl group was also making a pi-alkyl interaction with Trp219, while the phenyl ring was making a pi-alkyl interaction with Leu300 and a pi-pi stacked interaction with Trp219. The other phenyl ring was making a pi-pi T-shaped interaction with Phe122. By visualizing the hydrophobic surface of the enzyme in the binding site, it can be seen that the hydrophobic contacts play a significant role in enzyme inhibition (Fig. 11).

**Fig. 12:**
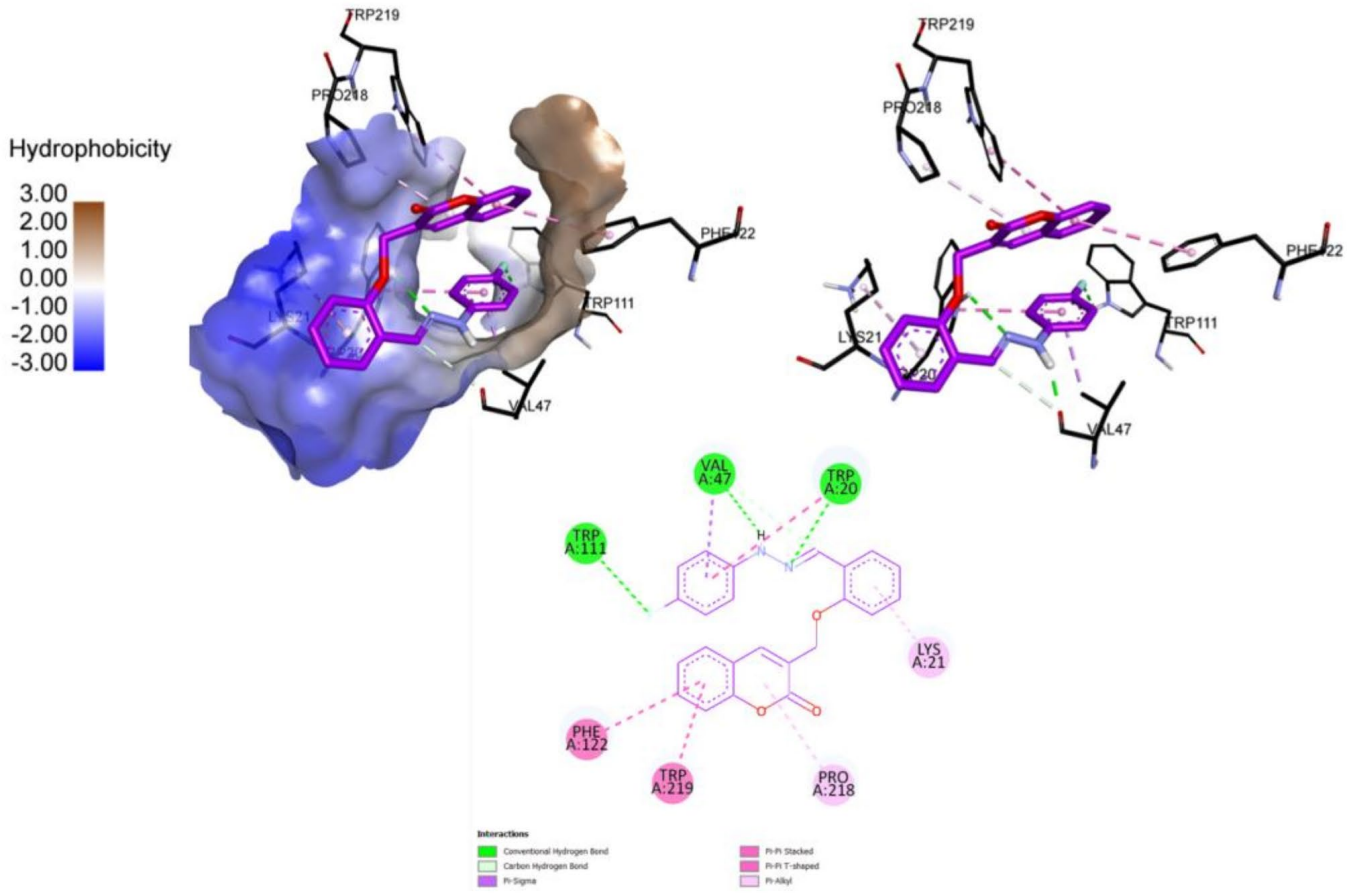
Binding site interactions of ALR2 inhibitor compound **6**.

Docking analysis of compound **6**, the second most active inhibitor revealed hydrogen bond formation between the *N*H group and Val47, while the azomethine nitrogen was making a hydrogen with Trp20. The fluoro group was making a halogen bond with Trp111. The coumarin ring was making a pi-alkyl interaction with Pro218, a pi-pi T-shaped interaction with Trp219 and a pi-pi stacked interaction with Phe122. Another pi-alkyl interaction was observed between the phenyl ring and Lys21. The fluoro phenyl ring was making a pi-sigma and a pi-pi stacked interaction with Val47 and Trp20, respectively (Fig. 12).

### Molecular docking of hALR1 inhibitors

**Fig. 13:**
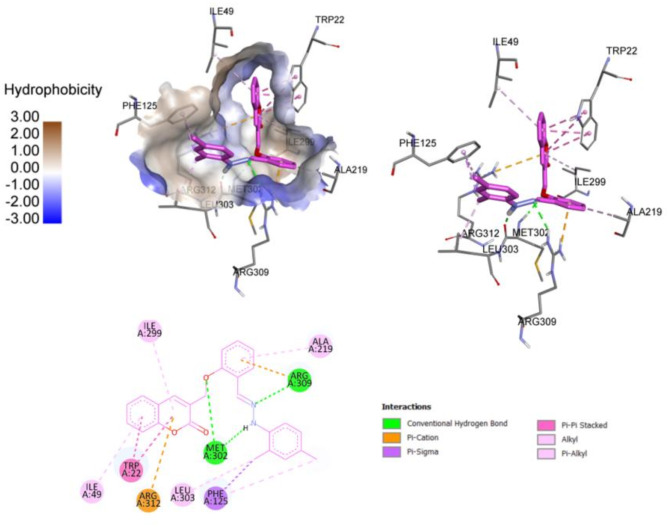
Binding site interactions of ALR1 inhibitor compound **20**.

Two most active ALR1 inhibitors compounds **9** and **20** were selected for the docking studies. Docking analysis of compound **20** showed hydrogen bonds between the *N*H group and Met302, the same amino acid Met302 was also making a hydrogen bond with oxygen atom of ether moiety of compound **20**. The azomethine nitrogen was making a hydrogen bond with Arg309. A number of hydrophobic interactions were also observed. Pi-alkyl interactions were observed between methyl groups substituted on the phenyl ring and Phe125 and Leu303, one of the methyl groups was also making a pi-sigma interaction with Phe125. The coumarin ring was making pi-alkyl interactions with Ile49 and Leu299. The phenyl ring in the middle part of the molecule was also making pi-alkyl interaction with Ala219. The phenyl ring was also making a pi-cation contact with Arg309, another pi-cation contact was observed between Arg312 and the coumarin ring. The coumarin ring was also making pi-pi stacked interactions with Trp22 (Fig. 13).

**Fig. 14:**
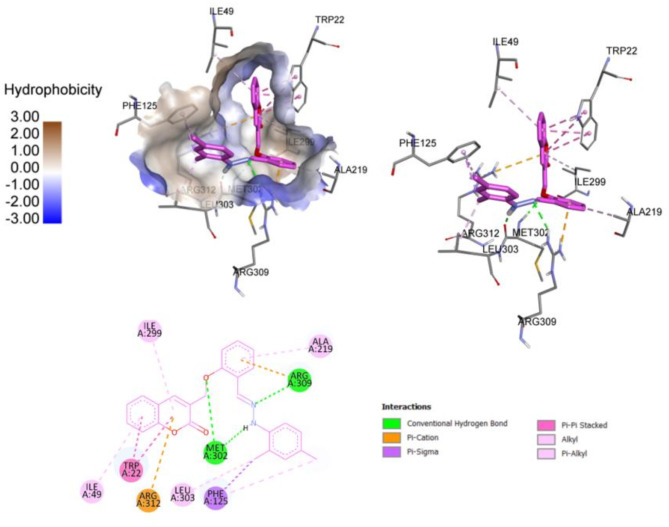
Binding site interactions of ALR1 inhibitor compound **9**.

Compound **9** was the second most active ALR1 inhibitor, its docking analysis showed hydrogen bond formation between *N*H group and Met302, the azomethine nitrogen was also making a hydrogen bond with Arg309. The coumarin ring was making a pi-alkyl interaction with Ile49 and pi-pi T-shaped interaction with Trp22, while the phenyl ring adjacent to it was making a pi-alkyl interaction with Ala219 (Fig. 14).

### *In silico* ADME evaluation

In order to predict the drug-likeness of the compounds,* in silico* ADME (Absorption, Distribution, Metabolism, and Excretion) properties of most active ALR2 (compounds **19**, and **6**) and ALDR1 (compounds **20** and **9**) inhibitors were determined using SwissADME^36^. No violations of the Lipinski’s rule were observed. The estimated parameters included calculation of molecular weights (MW), number of rotatable bond, hydrogen bond donors and acceptors. For ideal drug like molecules the molecular weight should not be greater than 500, all compounds had molecular weights < 500 in the range 388–404. The number of rotatable bonds in a molecule is a reflection of molecular flexibility; all compounds had six numbers of rotatable bonds indicating sufficient flexibility. Compounds **6** had five hydrogen bond acceptors and only one hydrogen bond donor, while the remaining compounds had four hydrogen bond acceptors and one hydrogen bond donor; these parameters are within the generally accepted values. The topological polar surface area (TPSA) is a measure of membrane permeability and its ideal value is less than 140 Å^2^. All compounds exhibited a highly favorable value of 63.8, hence all compounds are predicted to be able to cross the blood brain barrier (BBB). All compounds had high gastrointestinal (GI) absorption. The measure of lipophilicity is reflected by octanol/water partition coefficient (log P), and the ideal values should be in the range 1–5. All compounds indicated favorable values in the range 4.5–4.9. Water solubility is estimated by log S values, generally the lower values indicate sufficient water solubility. Compound **6** was the only compound that was predicted to have moderate water solubility, whereas the remaining three compounds indicated poor water solubility. P-Glycoprotein (Pgp) is a transport protein that forces foreign substances out of the cells. If a compound is a Pgp substrate, it can be expelled by Pgp. This may affect the drugs absorption and/or bioavailability resulting in reduced drug efficacy. Ideally a drug like molecule should not be a substrate of Pgp. All compounds fit this criterion. Cytochrome P450s are important enzymes that are responsible for metabolism of various types of drugs, to prevent inadvertant and adverse drug reactions; ideal drug like molecules should not inhibit cytochrome P450 and its sub-types (CYP1A2, CYP2C19, CYP2C9, CYP2D6, CYP3A4). None of the compounds was predicted to act as an inhibitor of CYP2D6. Compound **6** was also predicted not to inhibit CYP3A4. Compounds **9**, **19** and **20** may not inhibit CYP1A2 and CYP2D6. Overall the results are encouraging enough to continue exploring inhibitors from class of compounds as even more potent drug like compounds for possible treatment diabetic complications (Table 4).

**Table 4:** In silico ADME properties of ALR2 and ALR1 inhibitors.

Code	Compound 6	Compound 9	Compound 19	Compound 20
MW	388.39	404.85	398.45	398.45
#Rotatable bonds	6	6	6	6
#H-bond acceptors	5	4	4	4
#H-bond donors	1	1	1	1
TPSA	63.83	63.83	63.83	63.83
Log P	4.58	4.81	4.93	4.8
Log S	− 5.63	− 6.06	− 6.07	− 6.07
Solubility Class	Moderately soluble	Poorly soluble	Poorly soluble	Poorly soluble
GI absorption	High	High	High	High
BBB permeant	Yes	Yes	Yes	Yes
Pgp substrate	No	No	No	No
CYP1A2 inhibitor	Yes	No	No	No
CYP2C19 inhibitor	Yes	Yes	Yes	Yes
CYP2C9 inhibitor	Yes	Yes	Yes	Yes
CYP2D6 inhibitor	No	No	No	No
CYP3A4 inhibitor	No	Yes	Yes	Yes

## Conclusion

In general, a series of new coumarin-based Schiff base hybrids was prepared from newly synthesized DABCO-*C*_*7*_-F ionic liquid and compared with conventional method. Comparison of conventional method with DABCO-*C*_*7*_-F IL-based method revealed that relatively better yield for IL method. Also, reaction took 3 h for completion using IL while conventional method requires 6 h. The products were obtained in good to excellent yields as well as methodology was found to be efficient, environmentally benign, and widely applicable. Based on the reports on ALR inhibitory activity of coumarin and Schiff base analogues, synthesized hybrid compounds were further explored for their potential against ALR2 enzyme (IC_50_ = 1.61 to 11.20 *µ*M) and check for selectivity *via* screening against ALR1 enzyme. The structure–activity relationship studies described that compounds **6**, **8**, **9**, and **19** showed strong inhibitory potential for ALR2. Although, compounds **16**–**18**, and **22** showed less than 50% inhibition when substituents were changed to electron-withdrawing groups, such as nitro or cyano groups. This resulted in the full absence of activity for these compounds. Moreover, similar results for ALR2 inhibitory activity and selectivity was observed for the synthesized compounds as that of reported coumarin analogues while contrary to that of Schiff base analogues from the literature.

## Electronic supplementary material

Below is the link to the electronic supplementary material.

Supplementary Material 1

## Data Availability

The datasets used and/or analyzed during the current study available from the corresponding author on reasonable request.
