# Supplementary material for: Sustainable approach for synthesis of new coumarin-linked Schiff bases in DABCO-based ionic liquid and their identification as aldose reductase inhibitors
Source: Sci Rep. 2025 Apr 24;15:14400. doi: 10.1038/s41598-025-97949-6 (PMC12022153; doi:10.1038/s41598-025-97949-6)
Supplement: Supplementary file 1 — Supplementary Material 1 [file 41598_2025_97949_MOESM1_ESM.pdf]

Sustainable Approach for Synthesis of New Coumarin-Linked Schiff Bases in DABCO-Based Ionic Liquid and Their Identification as Aldose Reductase Inhibitors

Marium Ishtiaq[a,b], Naved Iqbal[a,b], Aqeel Imran[c,d], Mariya al-Rashida[f], Sobia Rana[g], Maria Aqeel Khan[a,b*], Jamshed Iqbal[c], and Abdul Hameed[b,e*]

[a]*Third World Center for Science and Technology, International Center for Chemical and Biological Sciences (ICCBS), University of Karachi, Karachi-75270, Pakistan*
[b]*H.E.J. Research Institute of Chemistry, International Center for Chemical and Biological Sciences (ICCBS), University of Karachi, Karachi-75270, Pakistan*
[c]*Center for Advanced Drug Research, COMSATS University Islamabad, Abbottabad Campus, Abbottabad-22060, Pakistan*
[d]*Department of Chemistry, COMSATS University Islamabad, Abbottabad Campus, Abbottabad-22060, Pakistan*
[e]*Department of Chemistry, University of Sahiwal, Sahiwal-57000, Pakistan*
[f]*Department of Chemistry, Forman Christian College (A Chartered University), Lahore, Pakistan*
[g]*Molecular Biology and Human Genetics Laboratory, Dr. Panjwani Center for Molecular Medicine and Drug Research (PCMD), International Center for Chemical and Biological Sciences (ICCBS), University of Karachi, Karachi-75270, Pakistan*

Email Addresses:

A. Hameed: abdul.hameed8@hotmail.com; drabdulhameed@uosahiwal.edu.pk
Maria A. Khan: markhan883@gmail.com; drmaria.aqeel@iccs.edu

Contents

Spectra of Coumarin-Linked Schiff bases ……………………………………………………….S4
^{1}H-NMR *of 3-((2-((2-Phenylhydrazineylidene)methyl)phenoxy)methyl)-2H-chromen-2-one* ***(5)***………………………………………………………………………………..……..S4
EIMS of *3-((2-((2-Phenylhydrazineylidene)methyl)phenoxy)methyl)-2H-chromen-2-one* (***5***)…………………………………………………………………………………………S5
^{1}H-NMR of *3-((2-((2-(4-Fluorophenyl)hydrazineylidene)methyl)phenoxy)methyl)-2H-chromen-2-one* ***(6)***…………………………………………………………………………………………S6
EIMS of *3-((2-((2-(4-Fluorophenyl)hydrazineylidene)methyl)phenoxy)methyl)-2H-chromen-2-one* ***(6)***…………………………………………………………………………………………S7
^{1}H-NMR of *3-((2-((2-(2-Chlorophenyl)hydrazineylidene)methyl)phenoxy)methyl)-2H-chromen-2-one* ***(7)***…………………………………………………………………………………………S8
EIMS of *3-((2-((2-(2-Chlorophenyl)hydrazineylidene)methyl)phenoxy)methyl)-2H-chromen-2-one* ***(7)***…………………………………………………………………………………………S9
^{1}H-NMR of *3-((2-((2-(3-Chlorophenyl)hydrazineylidene)methyl)phenoxy)methyl)-2H-chromen-2-one* *(8)*……………………………………………………………………………………...S10
EIMS of *3-((2-((2-(3-Chlorophenyl)hydrazineylidene)methyl)phenoxy)methyl)-2H-chromen-2-one* ***(8)***…………………………………………………………………………………………S11
^{1}H-NMR of *3-((2-((2-(4-Chlorophenyl)hydrazineylidene)methyl)phenoxy)methyl)-2H-chromen-2-one* ***(9)***…………………………………………………………………………………………S12
EIMS of *3-((2-((2-(2-Bromophenyl)hydrazineylidene)methyl)phenoxy)methyl)-2H-chromen-2-one* ***(10)*** ………………………………………………………………………………………S13
^{1}H-NMR of *3-((2-((2-(2-Bromophenyl)hydrazineylidene)methyl)phenoxy)methyl)-2H-chromen-2-one* ***(10)***……………………………………………………………………………………..S14
EIMS of *3-((2-((2-(4-Chlorophenyl)hydrazineylidene)methyl)phenoxy)methyl)-2H-chromen-2-one* ***(9)***…………………………………………………………………………………………S15
^{1}H-NMR of *3-((2-((2-(4-Bromophenyl)hydrazineylidene)methyl)phenoxy)methyl)-2H-chromen-2-one* ***(11)***……………………………………………………………………………………..S16
EIMS of *3-((2-((2-(4-Bromophenyl)hydrazineylidene)methyl)phenoxy)methyl)-2H-chromen-2-one* ***(11)***……………………………………………………………………………………..S17
^{1}H-NMR of *3-((2-((2-(o-Tolyl)hydrazineylidene)methyl)phenoxy)methyl)-2H-chromen-2-one* (***12***)……………………………………………………………………………………..S18
EIMS of *3-((2-((2-(o-Tolyl)hydrazineylidene)methyl)phenoxy)methyl)-2H-chromen-2-one* ***(12)***……………………………………………………………………………………..S19
^{1}H-NMR of *3-((2-((2-(p-Tolyl)hydrazineylidene)methyl)phenoxy)methyl)-2H-chromen-2-one* ***(13)***…………………………………………………………………………………….S20
EIMS of *3-((2-((2-(p-Tolyl)hydrazineylidene)methyl)phenoxy)methyl)-2H-chromen-2-one* ***(13)***…………………………………………………………………………………….S21
^{1}H-NMR of *3-((2-((2-(2-Ethylphenyl)hydrazineylidene)methyl)phenoxy)methyl)-2H-chromen-2-one* ***(14)***…………………………………………………………………………………….S22
EIMS of *3-((2-((2-(2-Ethylphenyl)hydrazineylidene)methyl)phenoxy)methyl)-2H-chromen-2-one* ***(14)***…………………………………………………………………………………….S23
^{1}H-NMR of *3-((2-((2-(4-Methoxyphenyl)hydrazineylidene)methyl)phenoxy)methyl)-2H-chromen-2-one* (***15***)…………………………………………………………………………...S24
EIMS of *3-((2-((2-(4-Methoxyphenyl)hydrazineylidene)methyl)phenoxy)methyl)-2H-chromen-2-one* ***(15)***…………………………………………………………………………………….S25
^{1}H-NMR of *4-(2-(2-((2-Oxo-2H-chromen-3-yl)methoxy)benzylidene)hydrazineyl)benzonitrile* ***(16)***…………………………………………………………………………………….S26
EIMS of *4-(2-(2-((2-Oxo-2H-chromen-3-yl)methoxy)benzylidene)hydrazineyl)benzonitrile* ***(16)***…………………………………………………………………………………….S27
^{1}H-NMR of *3-((2-((2-(2-Nitrophenyl)hydrazineylidene)methyl)phenoxy)methyl)-2H-chromen-2-one* ***(17)***…………………………………………………………………………………….S28

EIMS of *3-((2-((2-(2-Nitrophenyl)hydrazineylidene)methyl)phenoxy)methyl)-2H-chromen-2-one* ***(17)***...S29
^{1}H-NMR of *3-((2-((2-(2-Nitrophenyl)hydrazineylidene)methyl)phenoxy)methyl)-2H-chromen-2-one* ***(18)***...S30
EIMS of *3-((2-((2-(2-Nitrophenyl)hydrazineylidene)methyl)phenoxy)methyl)-2H-chromen-2-one* ***(18)***...S31
^{1}H-NMR of *3-((2-((2-(2,3-Dimethylphenyl)hydrazineylidene)methyl)phenoxy)methyl)-2H-chromen-2-one* ***(19)***...S32
EIMS *3-((2-((2-(2,3-Dimethylphenyl)hydrazineylidene)methyl)phenoxy)methyl)-2H-chromen-2-one* ***(19)***...S33
^{1}H-NMR of *3-((2-((2-(2,4-Dimethylphenyl)hydrazineylidene)methyl)phenoxy)methyl)-2H-chromen-2-one* ***(19)***...S34
EIMS *3-((2-((2-(2,4-Dimethylphenyl)hydrazineylidene)methyl)phenoxy)methyl)-2H-chromen-2-one* ***(19)***...S35
^{1}H-NMR of *3-((2-((2-(2,6-Dichlorophenyl)hydrazineylidene)methyl)phenoxy)methyl)-2H-chromen-2-one* ***(21)***...S36
EIMS *((2-((2-(2,6-Dichlorophenyl)hydrazineylidene)methyl)phenoxy)methyl)-2H-chromen-2-one* ***(21)***...S37
^{1}H-NMR of *3-((2-((2-(2,4-Dinitrophenyl)hydrazineylidene)methyl)phenoxy)methyl)-2H-chromen-2-one* ***(22)***...S38
EIMS of *3-((2-((2-(2,4-Dinitrophenyl)hydrazineylidene)methyl)phenoxy)methyl)-2H-chromen-2-one* ***(22)***...S39
Table-S1: *IC_{50} graphs of active compounds against ALR2 enzyme*.......................................S40
Table-S2: *IC_{50} graphs of active compounds against ALR1 enzyme*.......................................S43
Fig. S1. *Sequence alignment of target protein (hALR1) and template protein (porcine alcohol dehydrogenase, PDB id:3fx4a)*...S45
Fig. S2. *Ramachadran plot of homology model of hALR1*..S46
Fig. S3. *Overlap of target protein (hALR1, purple) with template protein (porcine, alcohol dehydrogenase, cyan). NADPH co-factor is shown in balls and sticks model*...........................S47
Table-S3: *Docking scores of active compounds against ALR2 enzyme.* S48
Fig. S4. *Overlap of ALR2 inhibitors* ***5-9****,* ***12-15****, and* ***19-21*** *(grey), all compounds bind with similar conformations in the same area of the binding site as the co-crystallized inhibitor (cyan)*...S49
Table-S4: *Docking scores of active compounds against ALR2 enzyme.* S50
Fig. S5. *Overlap of ALR1 inhibitors 7-****10, 14-15, 20*** *(grey), all compounds bind with similar conformations in the same area of the binding site as the co-crystallized inhibitor (cyan).* S51

Spectra of Coumarin-Linked Schiff bases

3-((2-((2-Phenylhydrazineylidene)methyl)phenoxy)methyl)-2H-chromen-2-one (5)

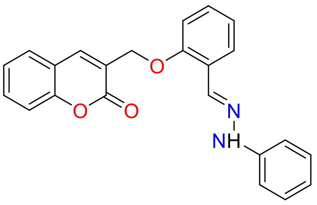

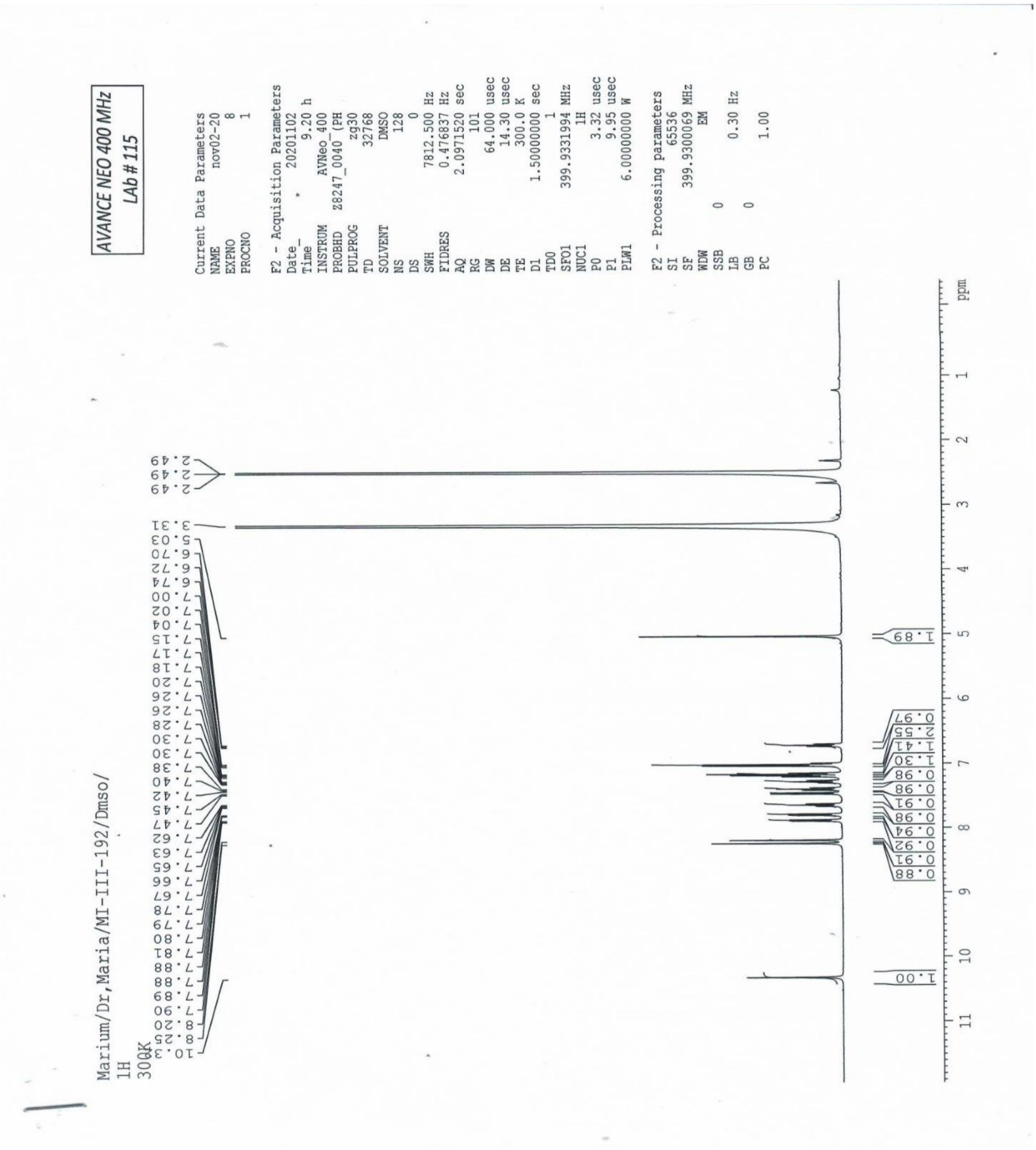

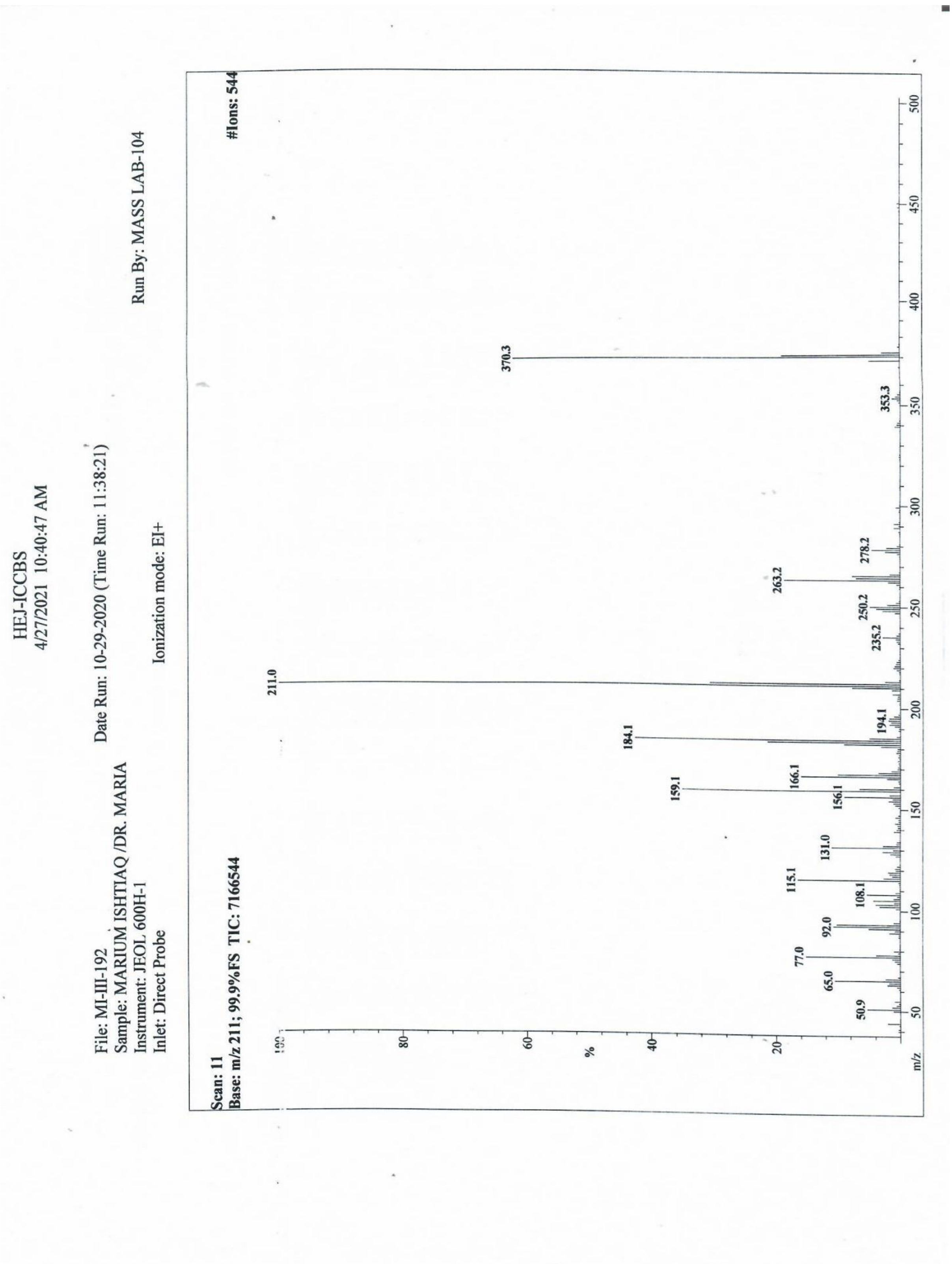
HEJ-ICCBS
4/27/2021 10:40:47 AM
File: MI-III-192
Sample: MARIUM ISHTIAQ /DR. MARIA
Instrument: JEOL 600H-1
Inlet: Direct Probe
Date Run: 10-29-2020 (Time Run: 11:38:21)
Ionization mode: EI+
Run By: MASS LAB-104
Scan: 11
Base: m/z 211; 99.9%FS TIC: 7166544
#Ions: 544
%
m/z
50.9
65.0
77.0
92.0
108.1
115.1
131.0
156.1
159.1
166.1
184.1
194.1
211.0
235.2
250.2
263.2
278.2
353.3
370.3

3-((2-((2-(4-Fluorophenyl)hydrazineylidene)methyl)phenoxy)methyl)-2H-chromen-2-one (6)

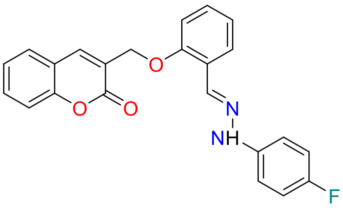

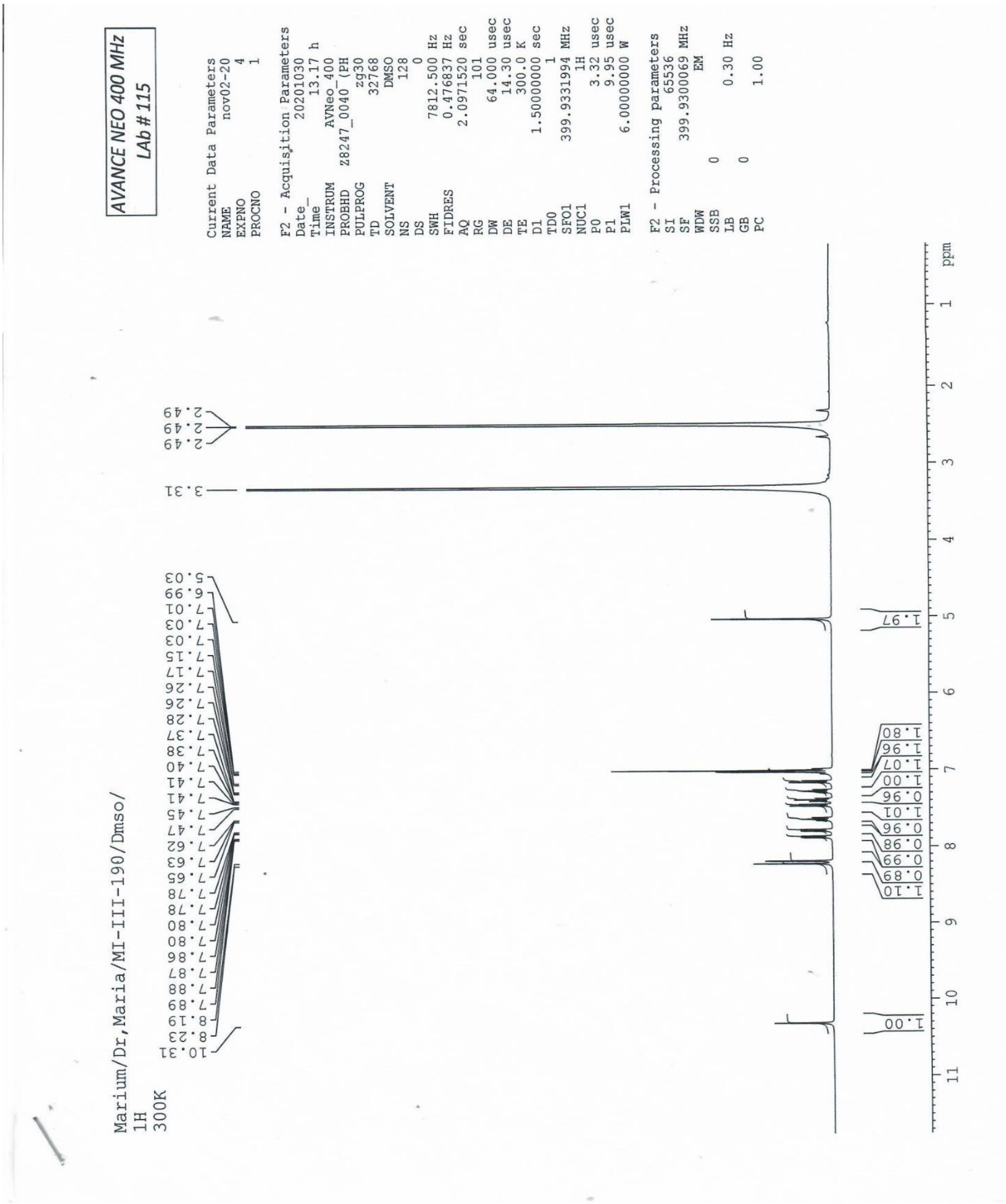

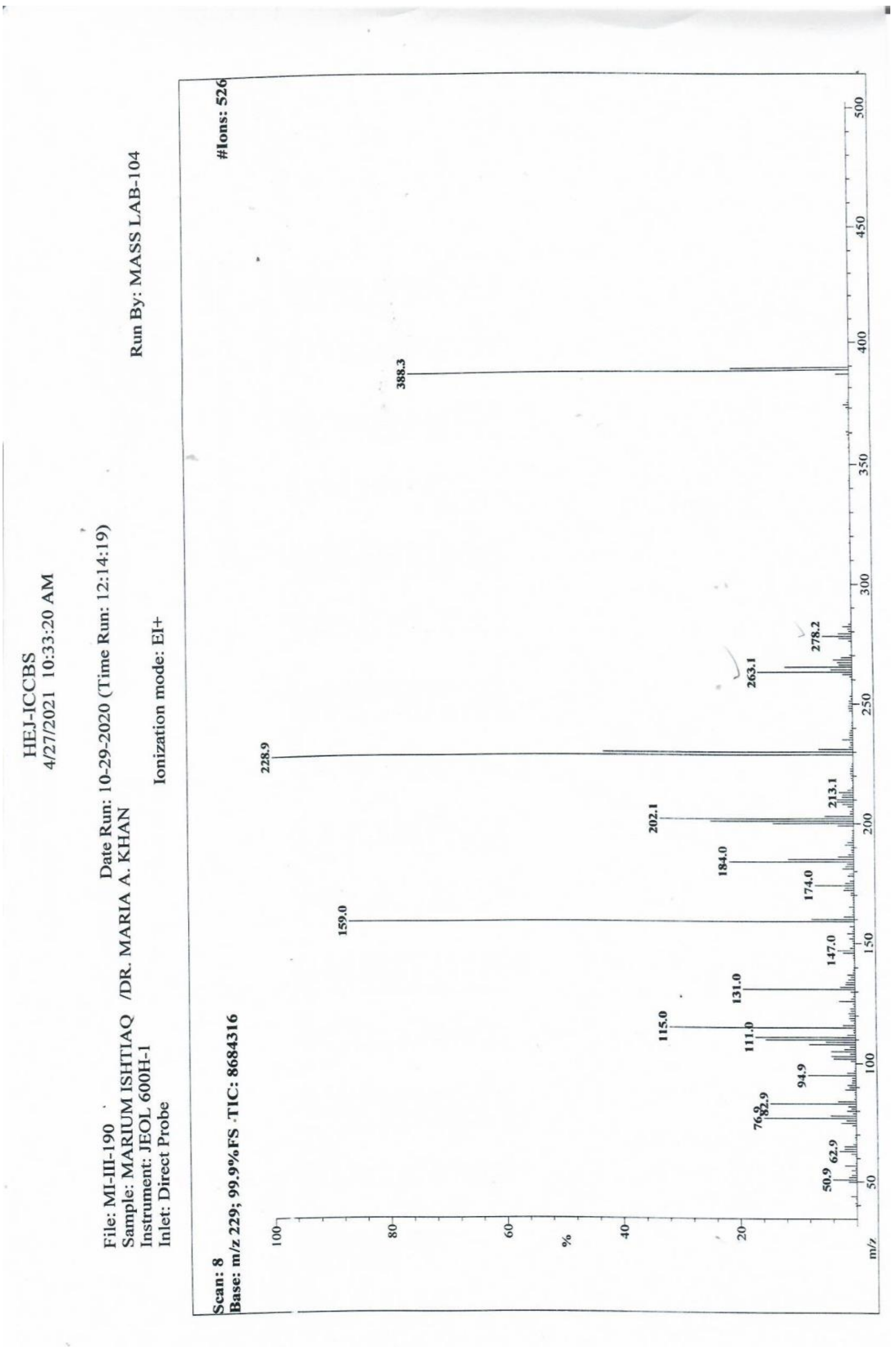
HEJ-ICCBS
4/27/2021 10:33:20 AM
File: MI-III-190
Date Run: 10-29-2020 (Time Run: 12:14:19)
Sample: MARIUM ISHTIAQ /DR. MARIA A. KHAN
Instrument: JEOL 600H-1
Run By: MASS LAB-104
Inlet: Direct Probe
Ionization mode: EI+
Scan: 8
Base: m/z 229; 99.9%FS TIC: 8684316
#Ions: 526
388.3
228.9
159.0
202.1
263.1
278.2
184.0
213.1
174.0
147.0
131.0
115.0
111.0
94.9
76.9
82.9
62.9
50.9
%
m/z

3-((2-((2-(2-Chlorophenyl)hydrazineylidene)methyl)phenoxy)methyl)-2H-chromen-2-one (7)

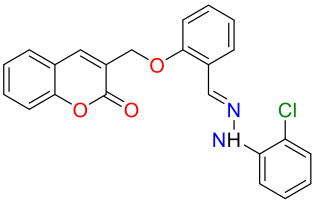

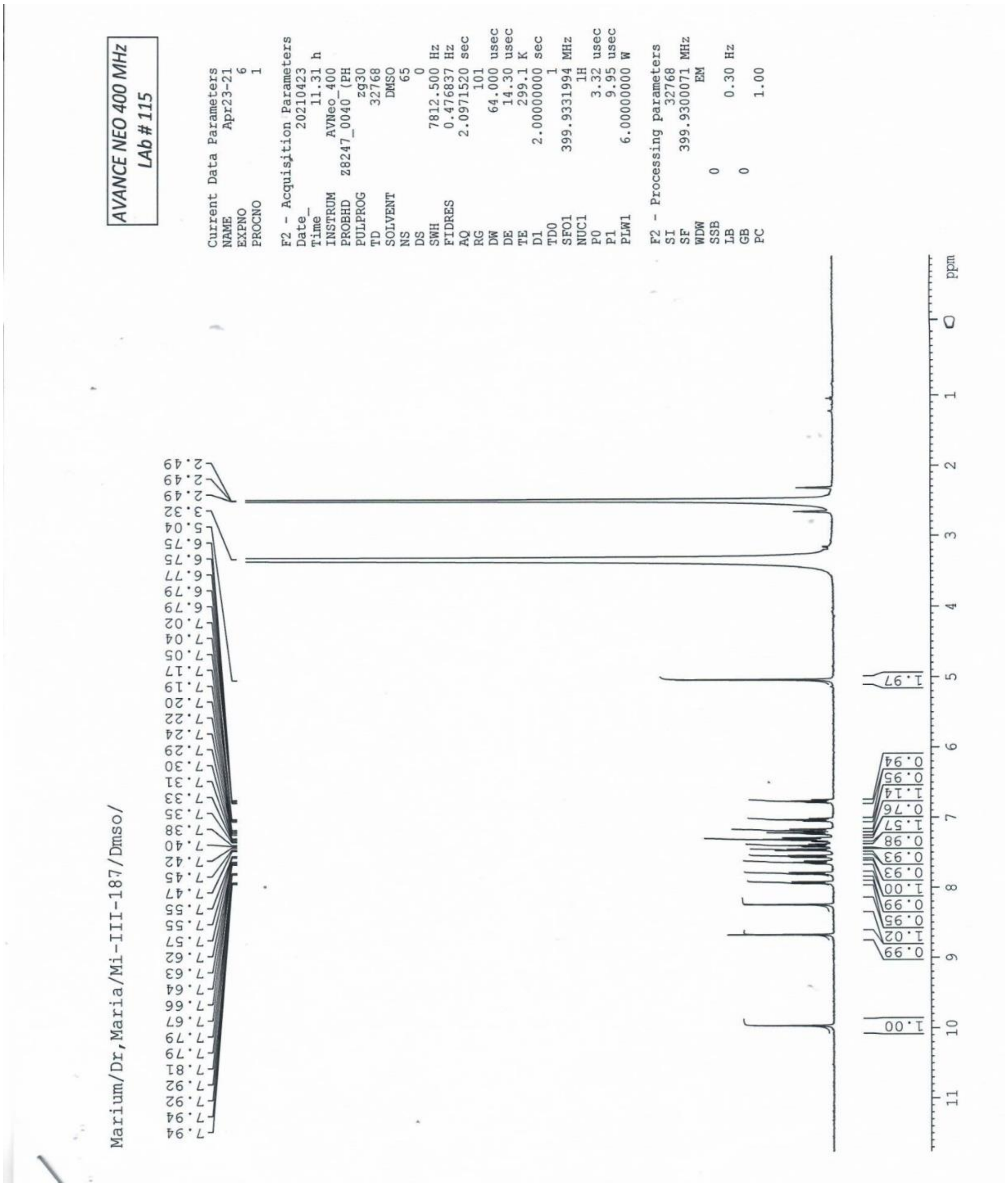

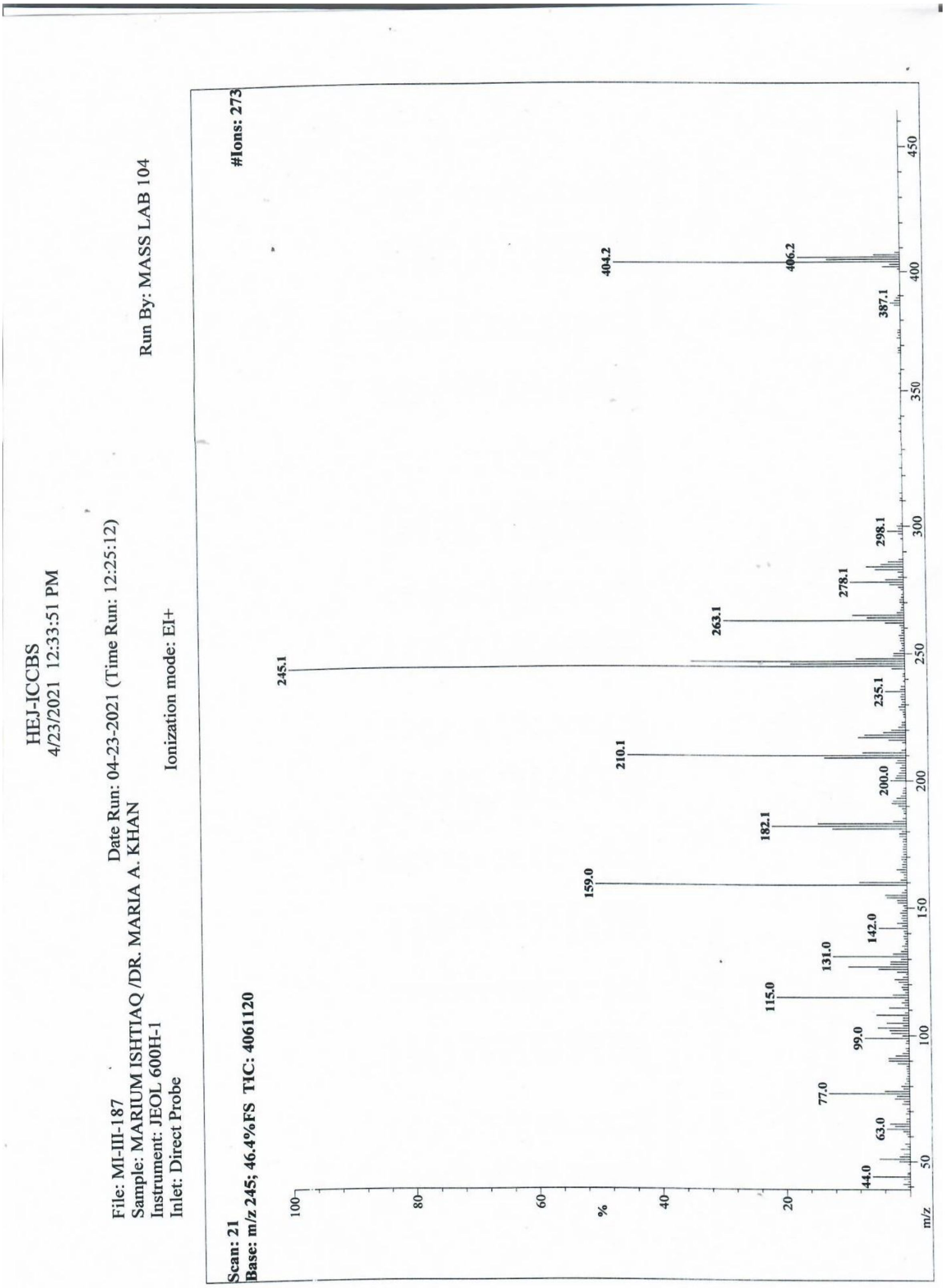
HEJ-ICCBS
4/23/2021 12:33:51 PM
File: MI-III-187
Date Run: 04-23-2021 (Time Run: 12:25:12)
Sample: MARIUM ISHTIAQ /DR. MARIA A. KHAN
Instrument: JEOL 600H-1
Run By: MASS LAB 104
Inlet: Direct Probe
Ionization mode: EI+
Scan: 21
#Ions: 273
Base: m/z 245; 46.4%FS TIC: 4061120
%
m/z
44.0
63.0
77.0
99.0
115.0
131.0
142.0
159.0
182.1
200.0
210.1
235.1
245.1
263.1
278.1
298.1
387.1
404.2
406.2

3-((2-((2-(3-Chlorophenyl)hydrazineylidene)methyl)phenoxy)methyl)-2H-chromen-2-one (8)

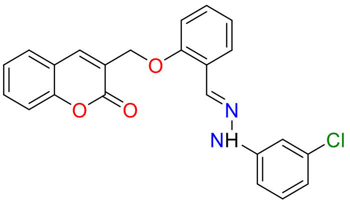

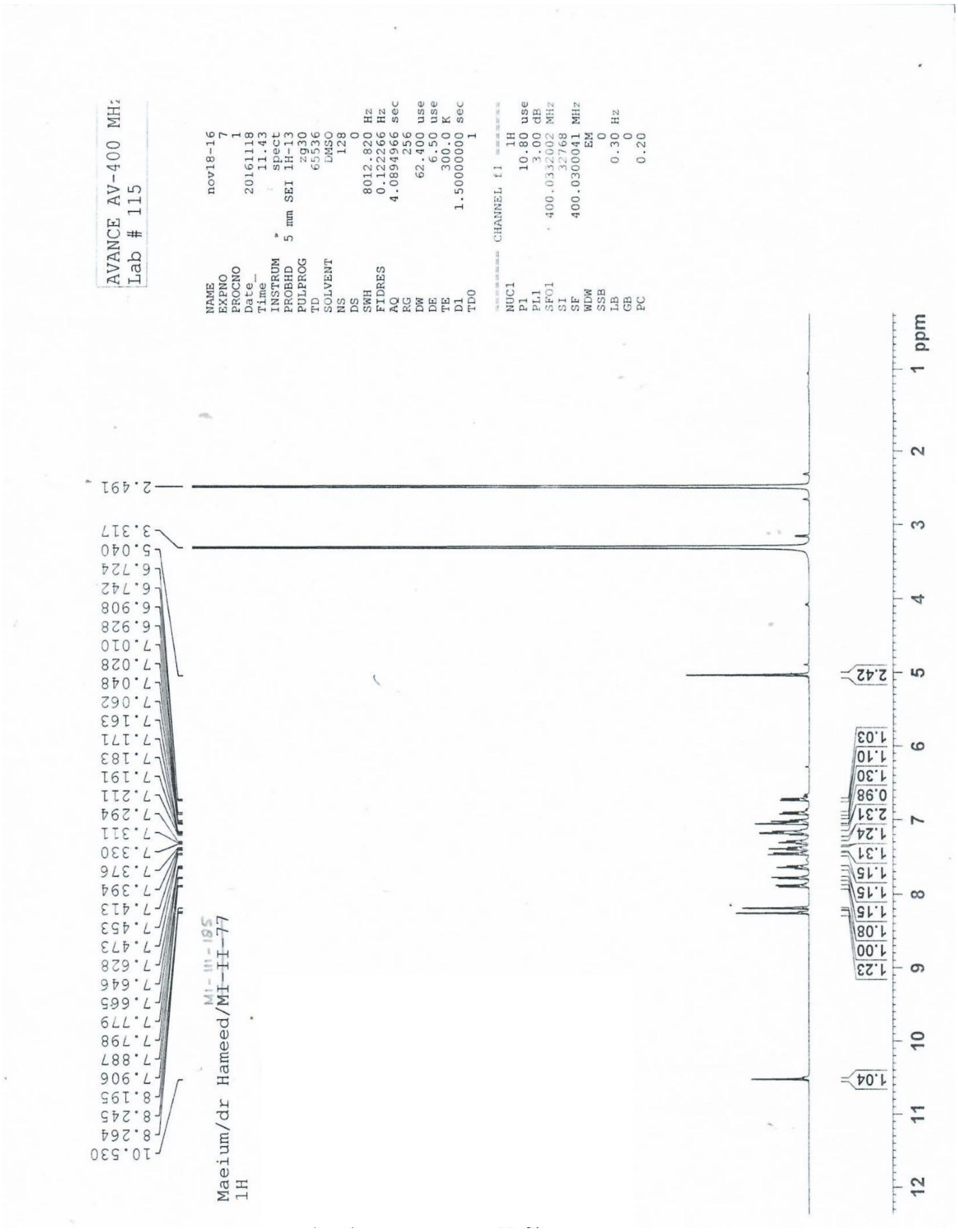

HEJ-ICCBS
4/23/2021 1:34:21 PM

File: MI-III-185 Date Run: 04-23-2021 (Time Run: 13:27:43)
Sample: MARIUM ISHTIAQ /DR. MARIA A. KHAN
Instrument: JEOL 600H-1 Run By: MASS LAB 104
Inlet: Direct Probe Ionization mode: EI+

Scan: 15
Base: m/z 245; 59.9%FS TIC: 4823888 #Ions: 299

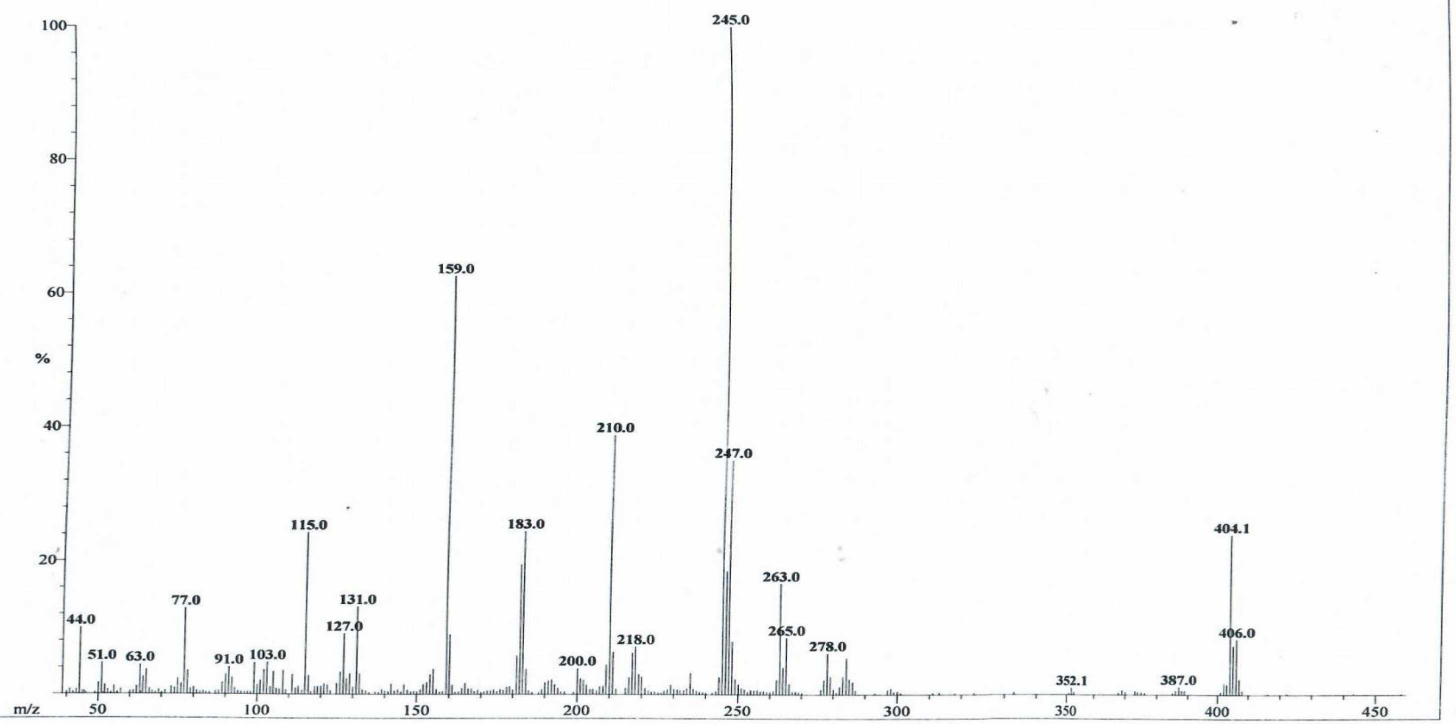

3-((2-((2-(4-Chlorophenyl)hydrazineylidene)methyl)phenoxy)methyl)-2H-chromen-2-one (9)

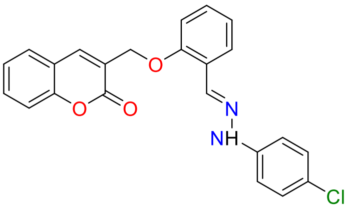

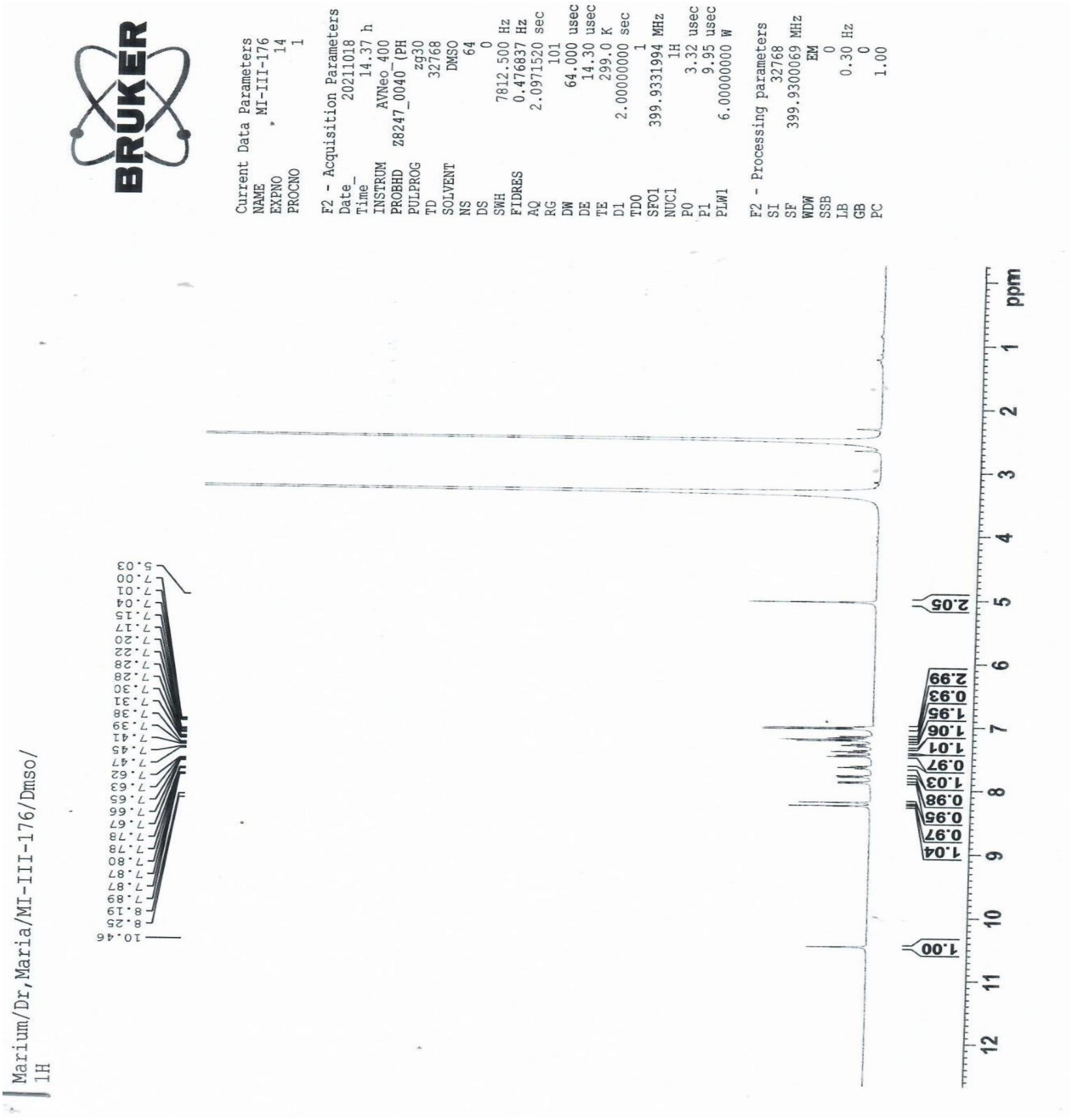

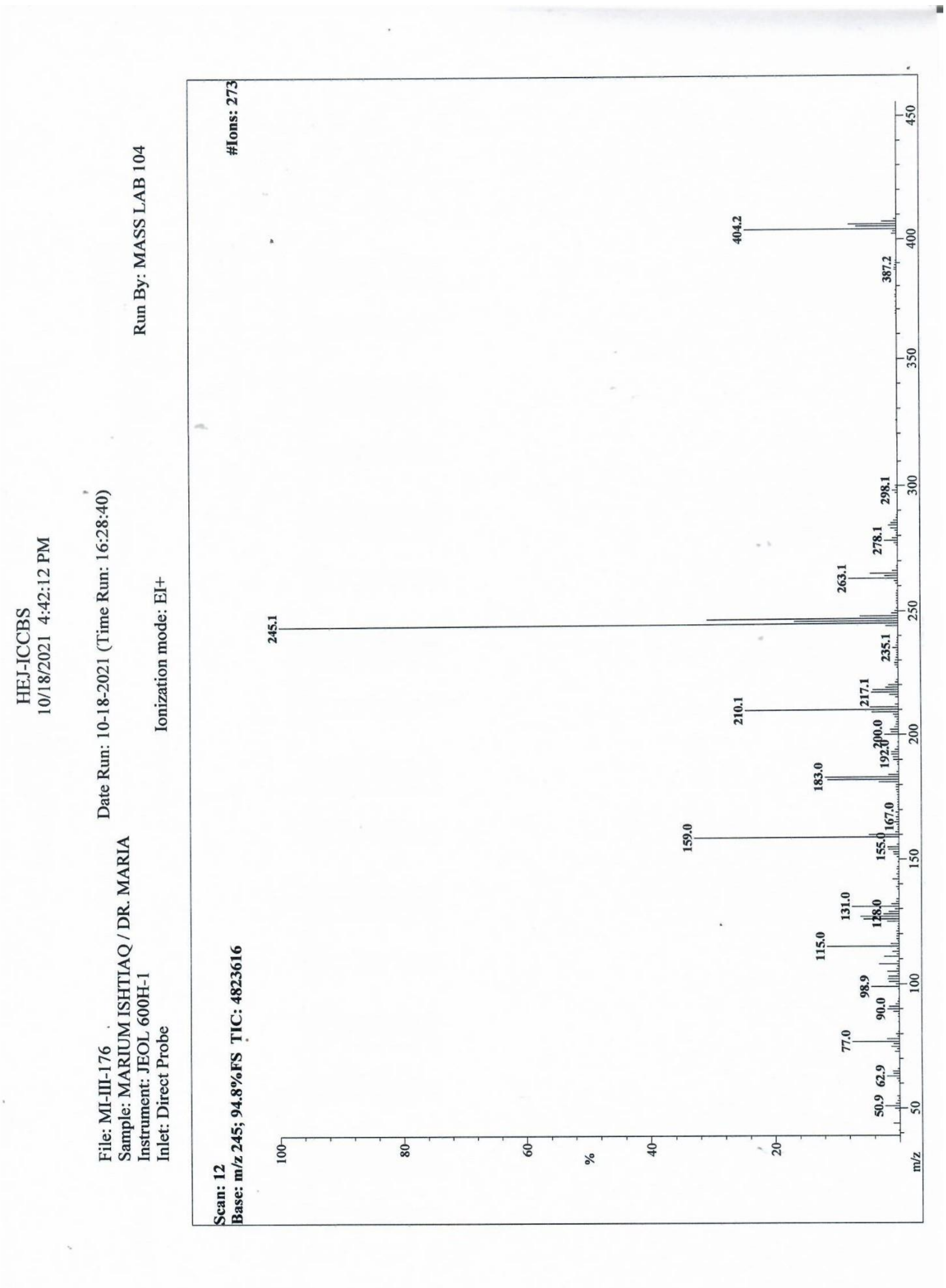
HEJ-ICCBS
10/18/2021 4:42:12 PM
File: MI-III-176
Sample: MARIUM ISHTIAQ / DR. MARIA
Instrument: JEOL 600H-1
Inlet: Direct Probe
Date Run: 10-18-2021 (Time Run: 16:28:40)
Ionization mode: EI+
Run By: MASS LAB 104
Scan: 12
Base: m/z 245; 94.8%FS TIC: 4823616
#Ions: 273
%
m/z
50.9
62.9
77.0
90.0
98.9
115.0
128.0
131.0
155.0
159.0
167.0
183.0
192.1
200.0
210.1
217.1
235.1
245.1
263.1
278.1
298.1
387.2
404.2

3-((2-((2-(2-Bromophenyl)hydrazineylidene)methyl)phenoxy)methyl)-2H-chromen-2-one (10)

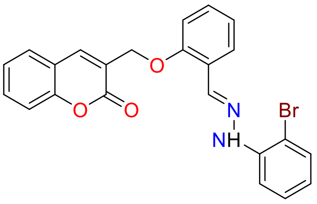

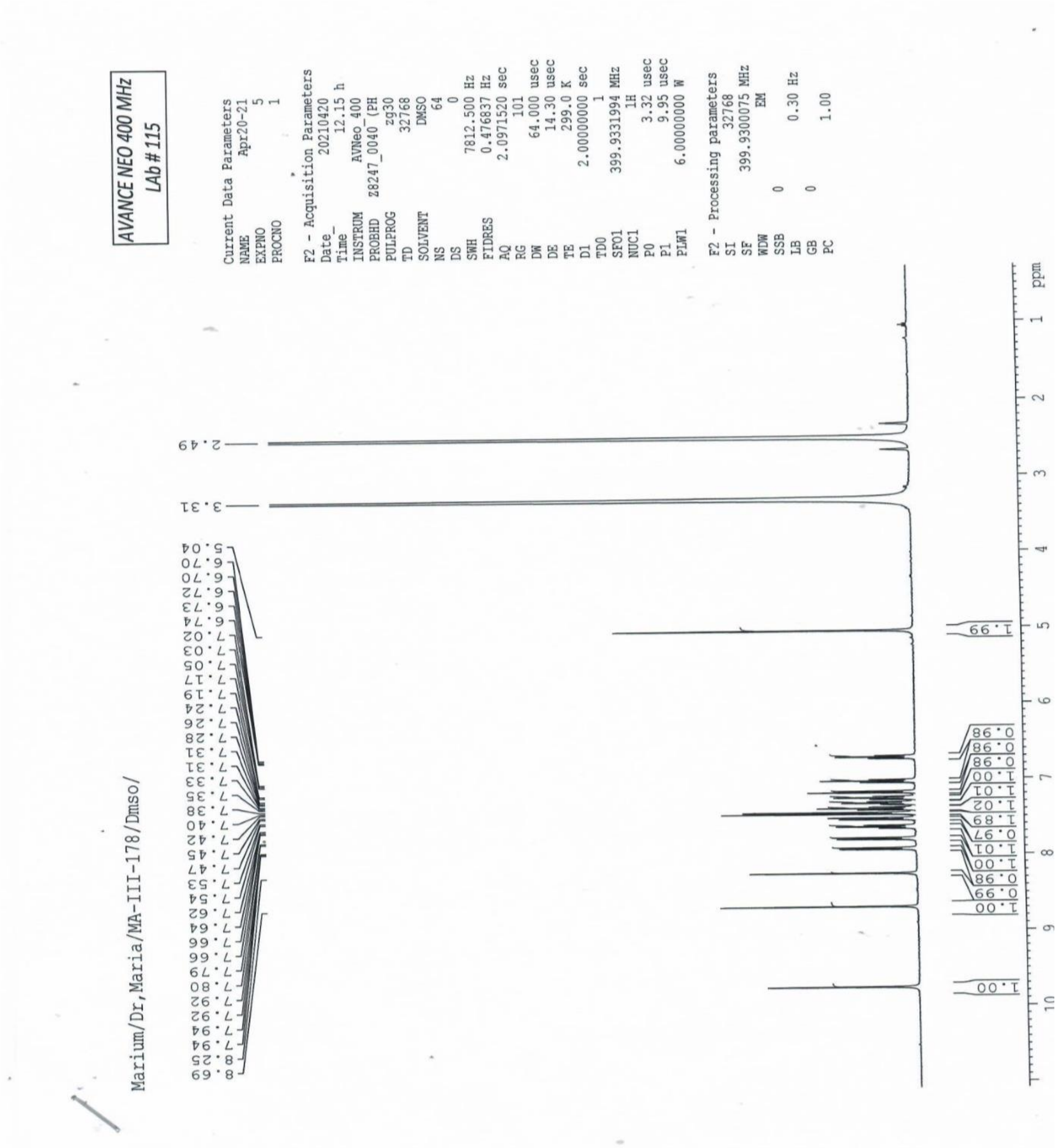

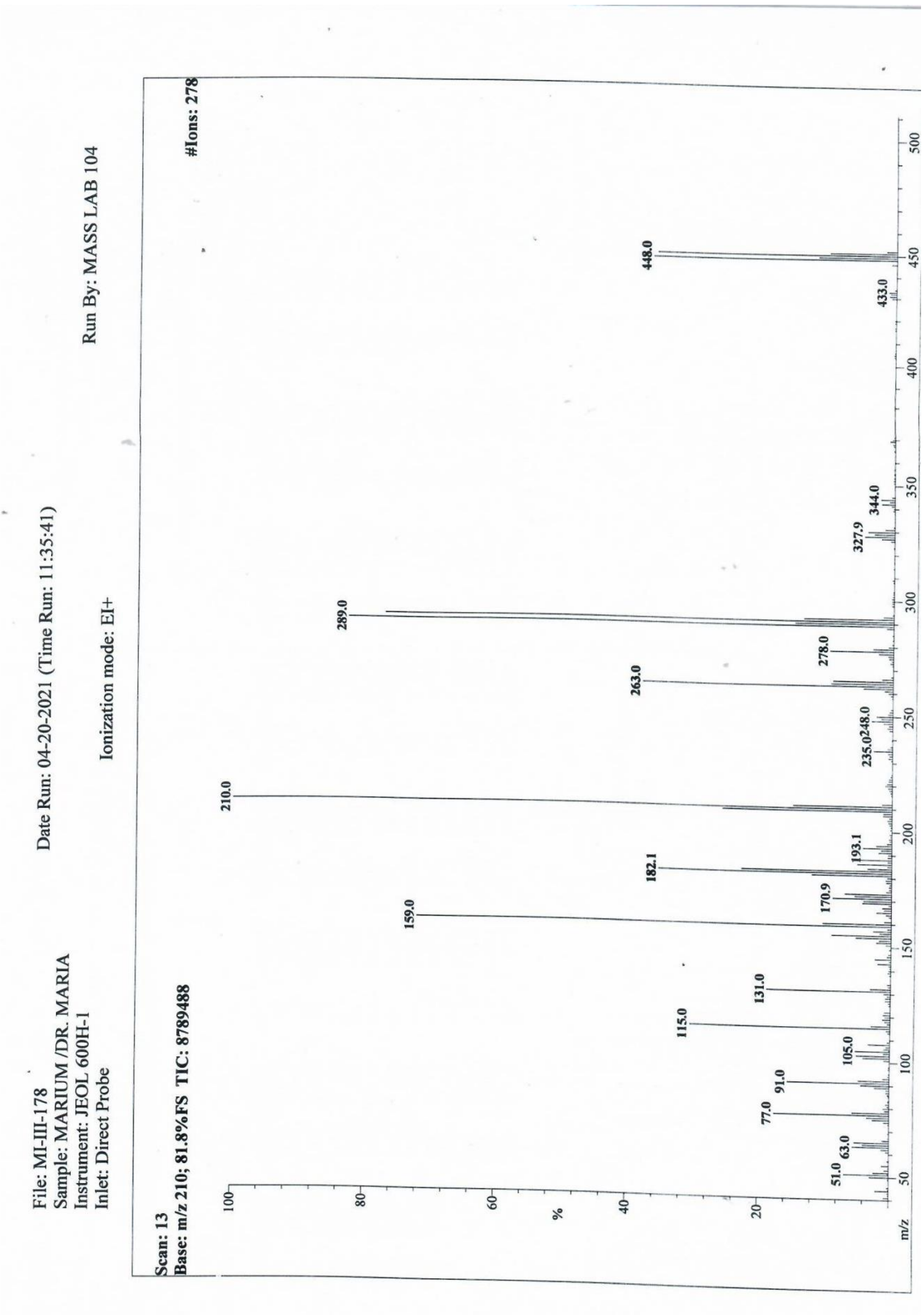
File: MI-III-178
Sample: MARIUM /DR. MARIA
Instrument: JEOL 600H-1
Inlet: Direct Probe
Date Run: 04-20-2021 (Time Run: 11:35:41)
Ionization mode: EI+
Run By: MASS LAB 104
Scan: 13
Base: m/z 210; 81.8%FS TIC: 8789488
#Ions: 278
%
m/z
51.0
63.0
77.0
91.0
105.0
115.0
131.0
159.0
170.9
182.1
193.1
210.0
235.0
248.0
263.0
278.0
289.0
327.9
344.0
433.0
448.0

3-((2-((2-(4-Bromophenyl)hydrazineylidene)methyl)phenoxy)methyl)-2H-chromen-2-one (11)

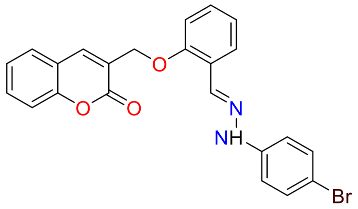

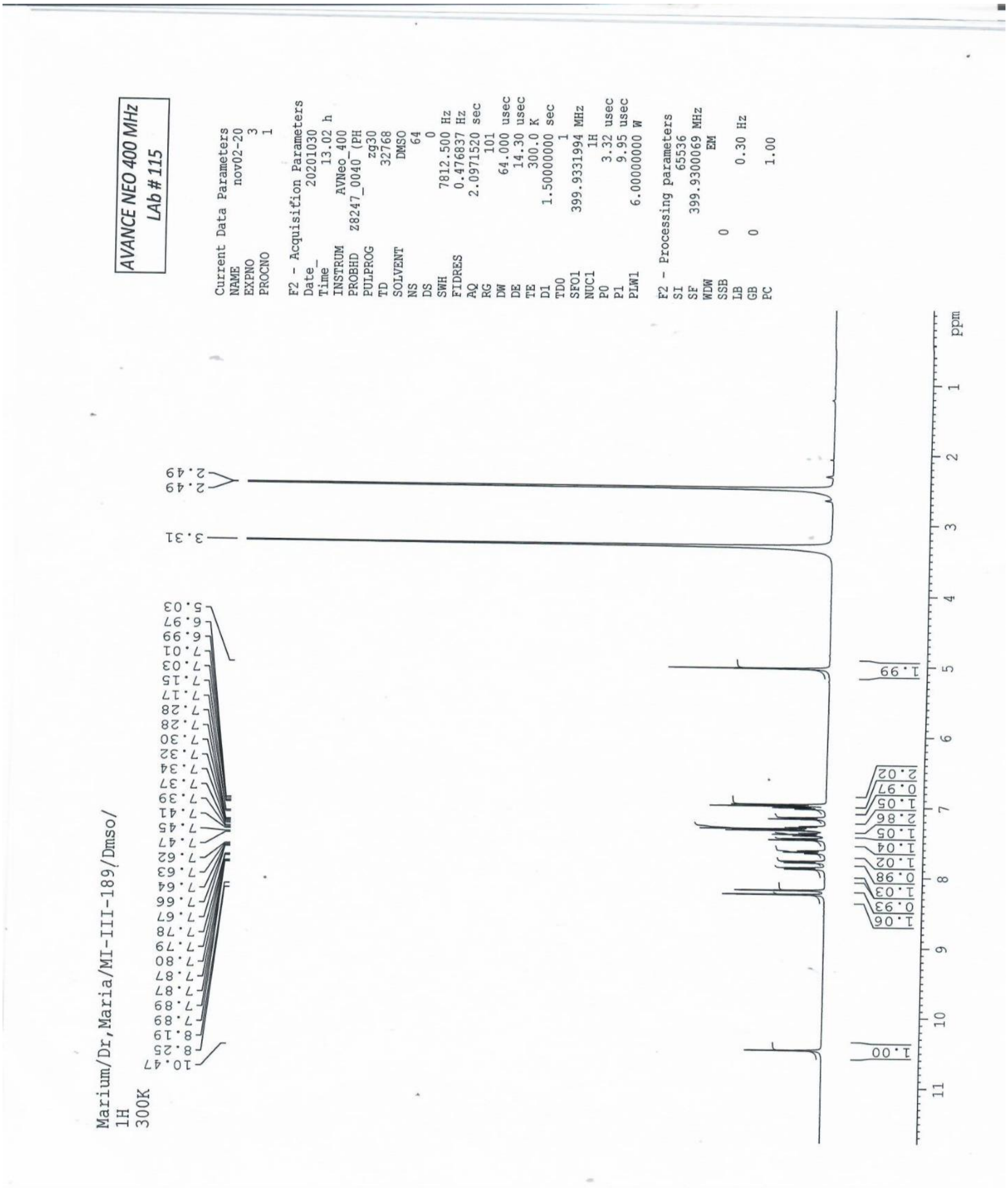

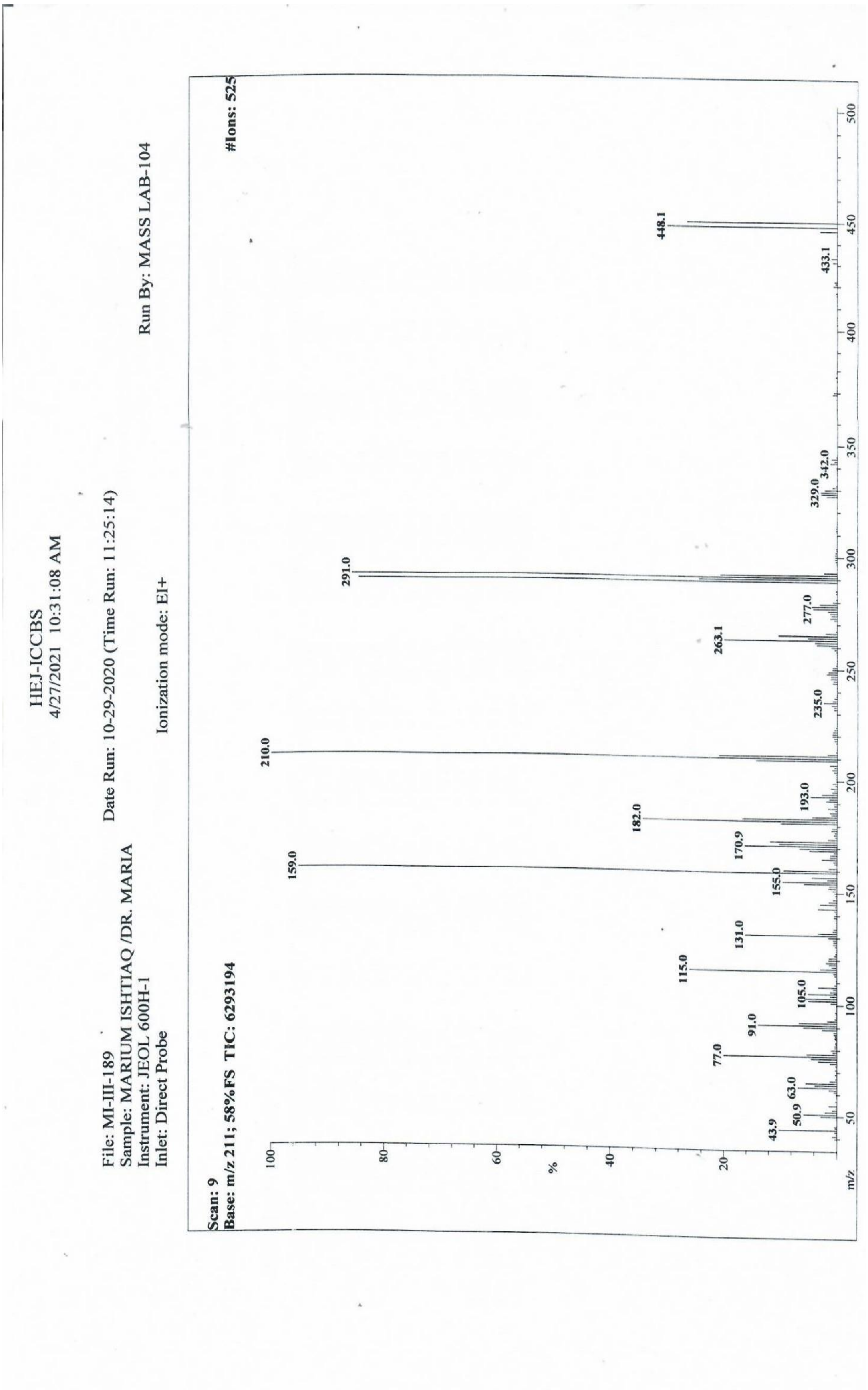
HEJ-ICCBS
4/27/2021 10:31:08 AM
File: MI-III-189
Sample: MARIUM ISHTIAQ /DR. MARIA
Instrument: JEOL 600H-1
Inlet: Direct Probe
Date Run: 10-29-2020 (Time Run: 11:25:14)
Ionization mode: EI+
Run By: MASS LAB-104
Scan: 9
Base: m/z 211; 58%FS TIC: 6293194
#Ions: 525
43.9
50.9
63.0
77.0
91.0
105.0
115.0
131.0
155.0
159.0
170.9
182.0
193.0
210.0
235.0
263.1
277.0
291.0
329.0
342.0
433.1
448.1
100
80
60
%
40
20
m/z
50
100
150
200
250
300
350
400
450
500

3-((2-((2-(o-Tolyl)hydrazineylidene)methyl)phenoxy)methyl)-2H-chromen-2-one (12)

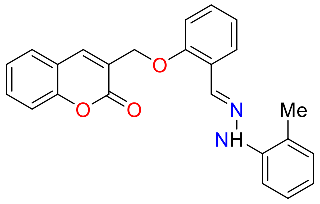

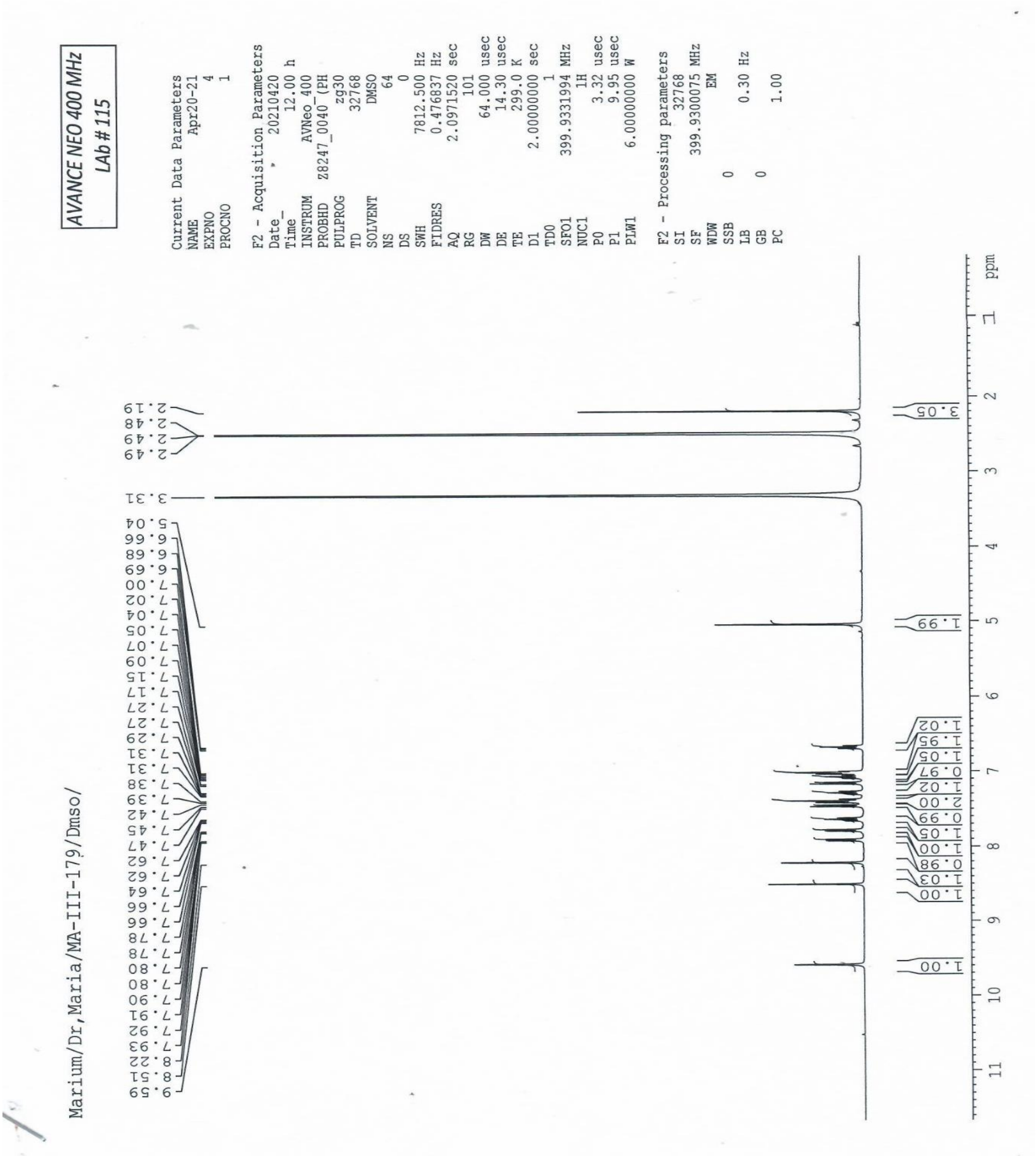

HEJ-ICCBS
4/20/2021 12:20:40 PM

File: MI-III-179
Sample: MARIUM /DR. MARIA
Instrument: JEOL 600H-1
Inlet: Direct Probe

Date Run: 04-20-2021 (Time Run: 12:14:03)

Run By: MASS LAB 104

Ionization mode: EI+

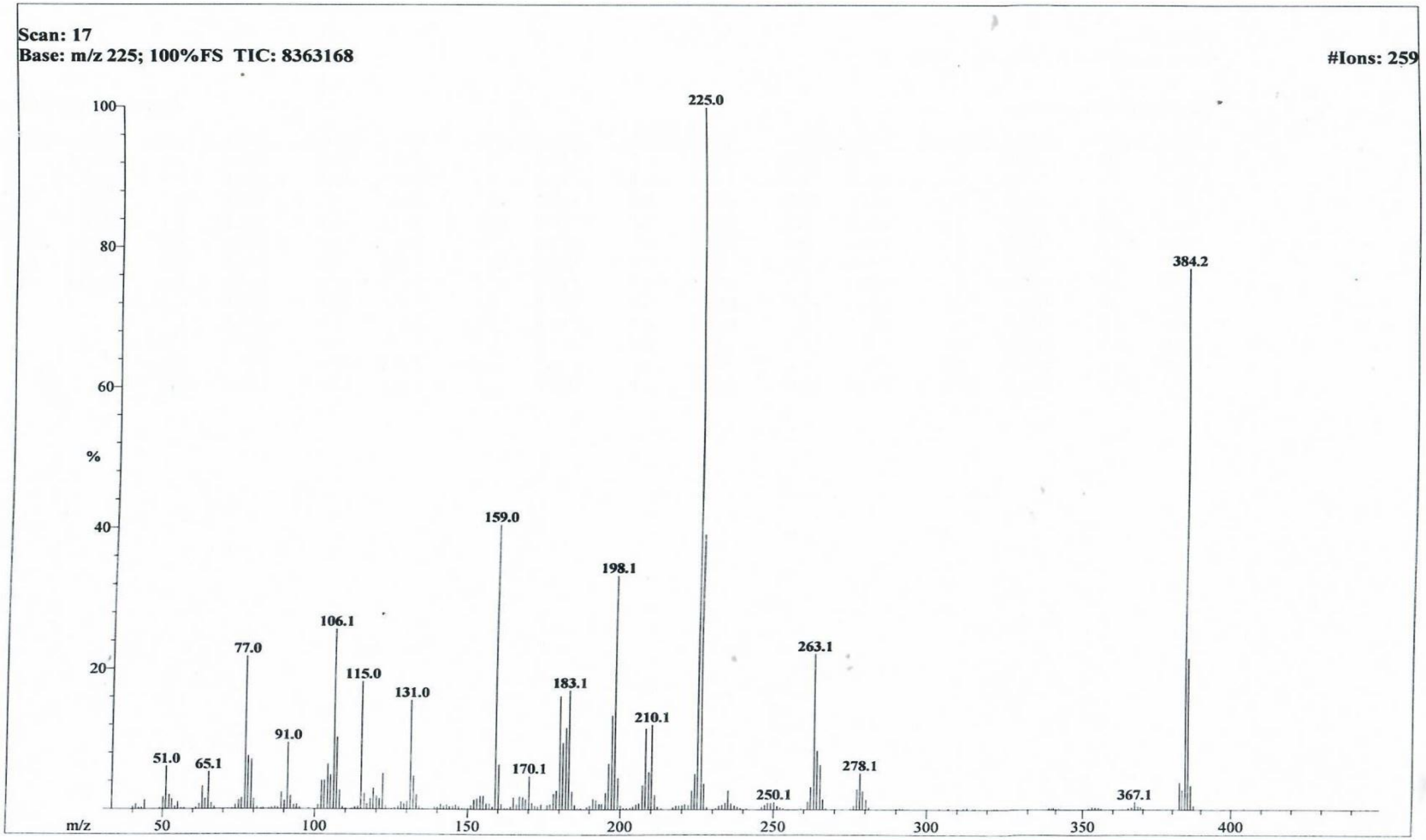

3-((2-((2-(p-Tolyl)hydrazineylidene)methyl)phenoxy)methyl)-2H-chromen-2-one (13)

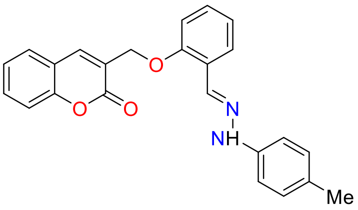

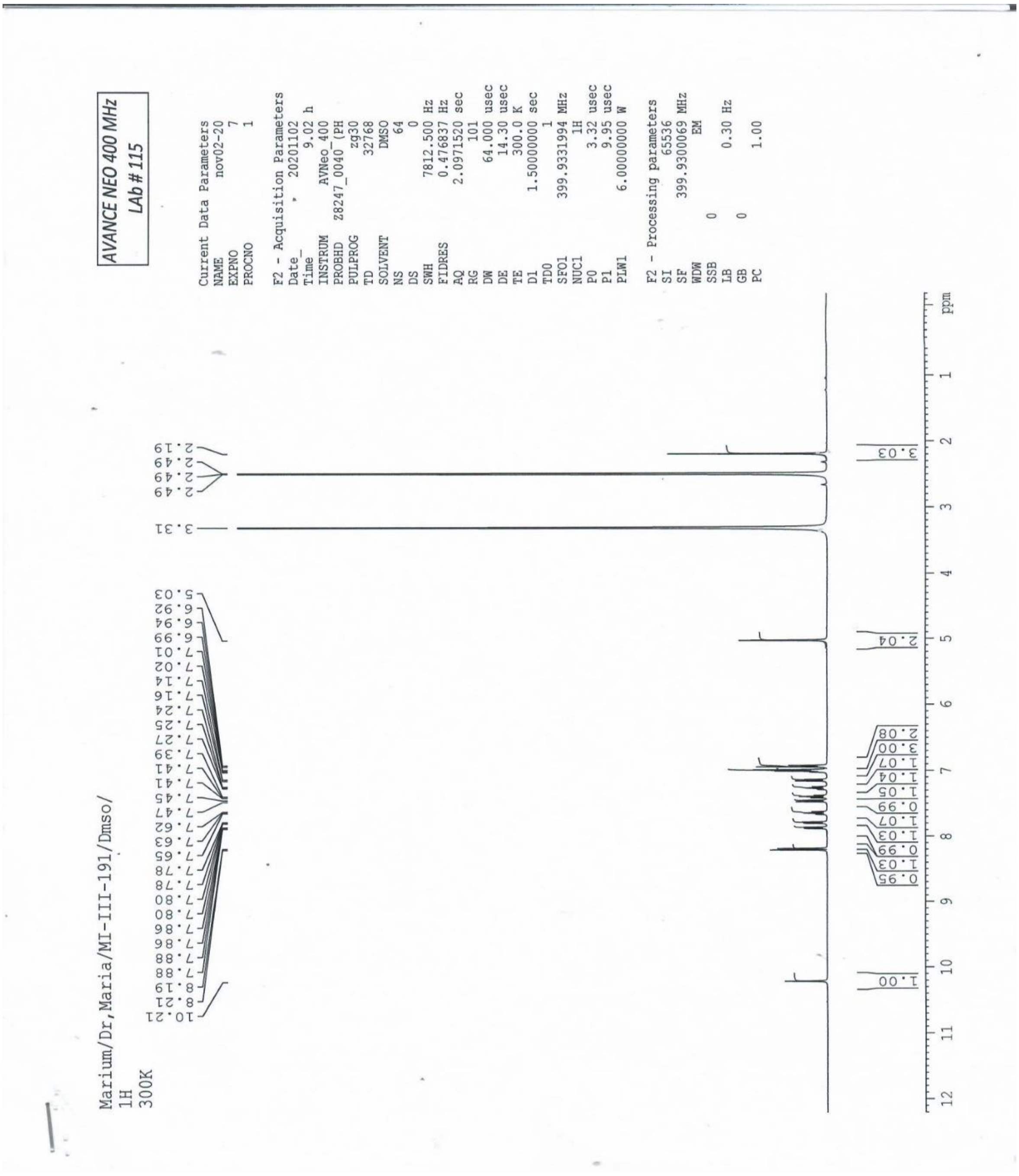

HEJ-ICCBS
5/19/2021 2:23:31 PM

File: MI-III-191
Sample: MARIUM ISHTIAQ /DR. MARIA
Instrument: JEOL 600H-1
Inlet: Direct Probe

Date Run: 10-29-2020 (Time Run: 11:48:24)

Ionization mode: EI+

Run By: MASS LAB-104

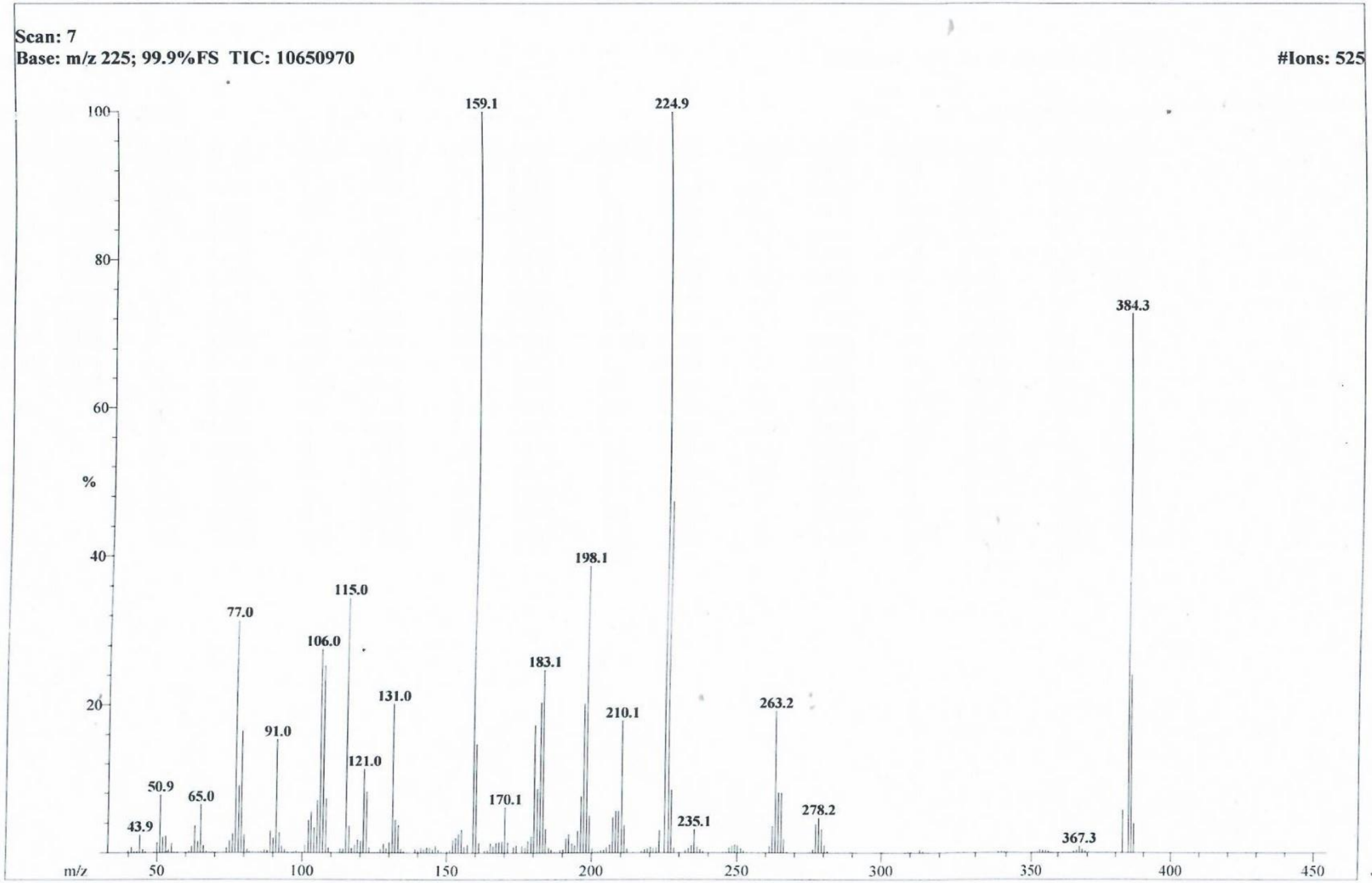

3-((2-((2-(2-Ethylphenyl)hydrazineylidene)methyl)phenoxy)methyl)-2H-chromen-2-one (14)

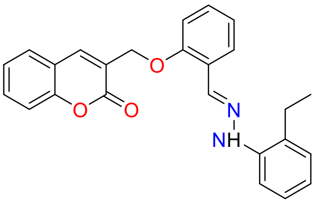

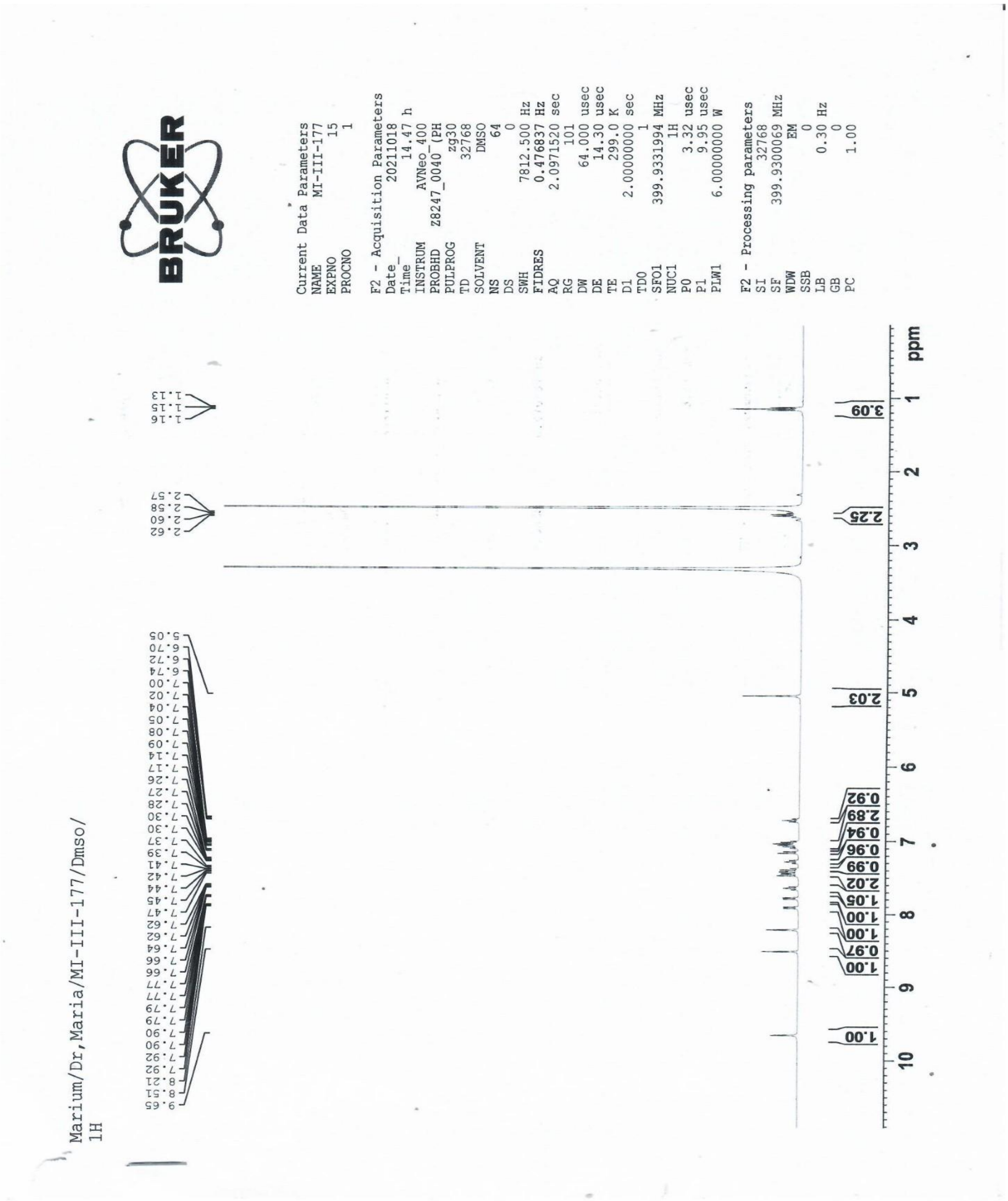

HEJ-ICCBS
10/18/2021 4:29:17 PM

File: MI-III-177'
Sample: MARIUM ISHTIAQ / DR. MARIA
Instrument: JEOL 600H-1
Inlet: Direct Probe

Date Run: 10-18-2021 (Time Run: 16:19:44)

Ionization mode: EI+

Run By: MASS LAB 104

Scan: 12
Base: m/z 239; 75.8%FS TIC: 4047392 **#Ions: 241**

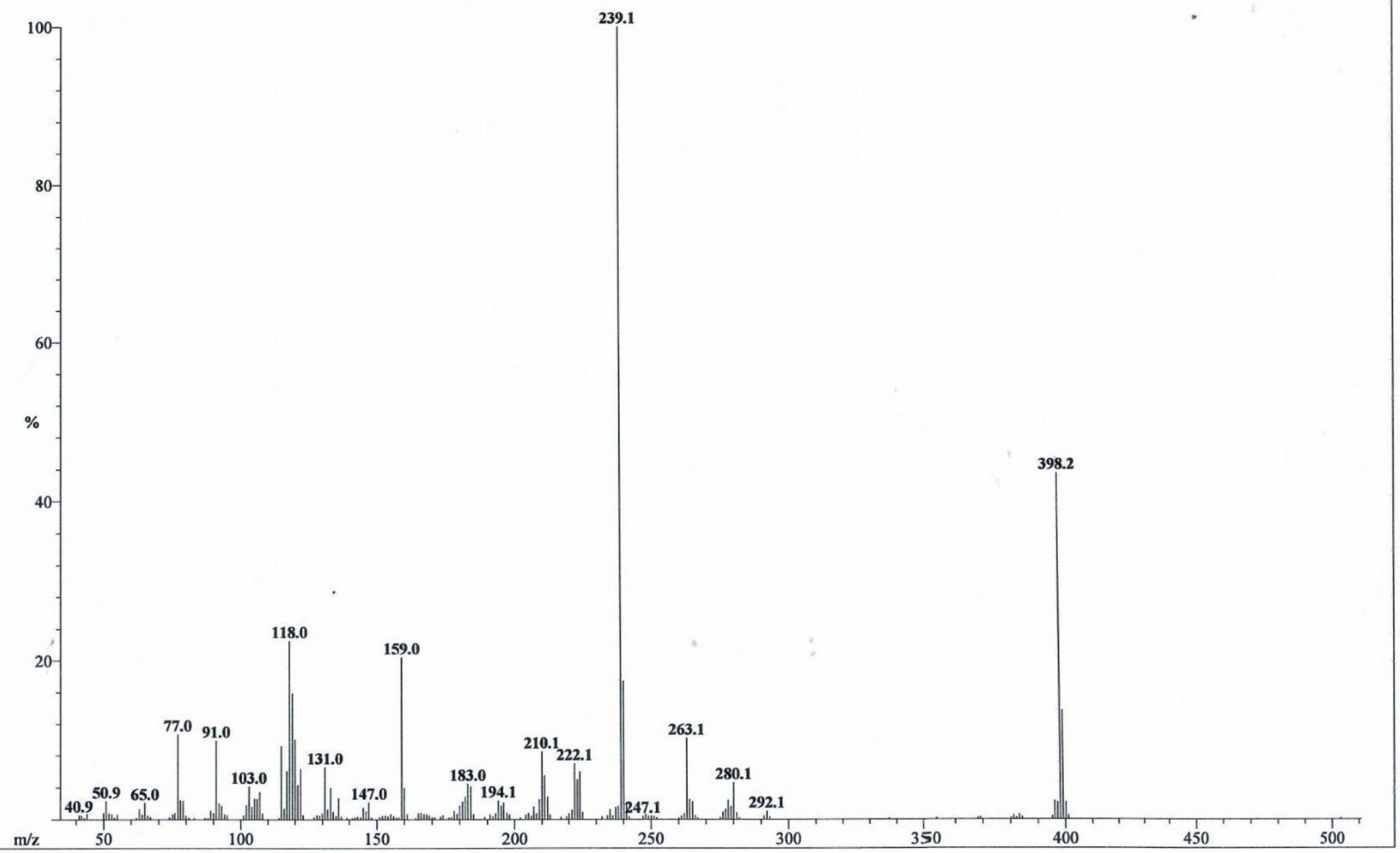

3-((2-((2-(4-Methoxyphenyl)hydrazineylidene)methyl)phenoxy)methyl)-2H-chromen-2-one (15)

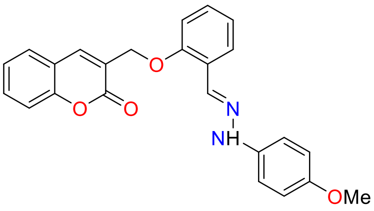

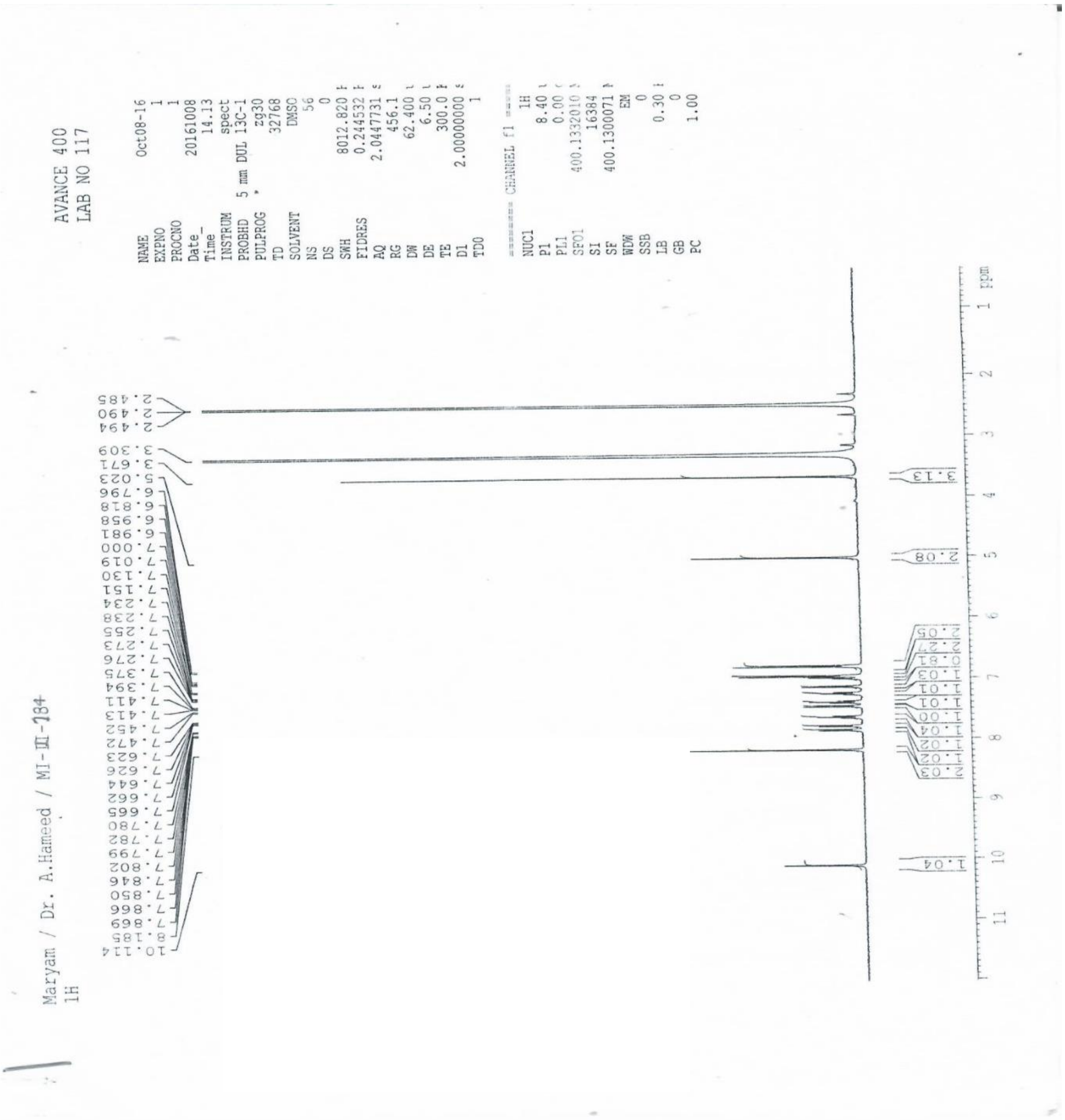

HEJ-ICCBS
4/23/2021 12:59:08 PM

File: MI-III-184 Date Run: 04-23-2021 (Time Run: 12:48:35)
Sample: MARIUM ISHTIAQ /DR. MARIA A. KHAN
Instrument: JEOL 600H-1 Run By: MASS LAB 104
Inlet: Direct Probe Ionization mode: EI+

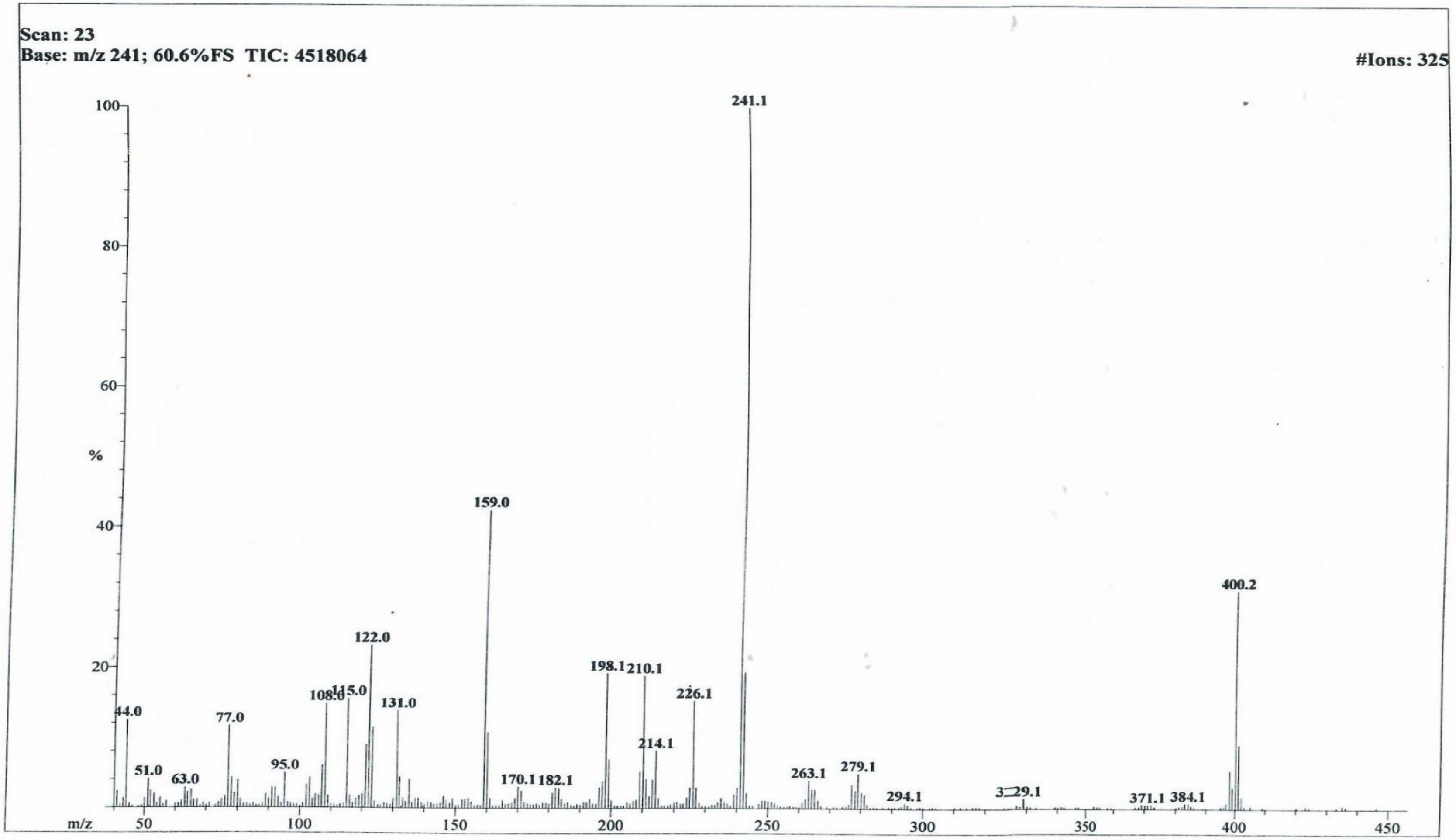

4-(2-(2-((2-Oxo-2H-chromen-3-yl)methoxy)benzylidene)hydrazineyl)benzonitrile (16)

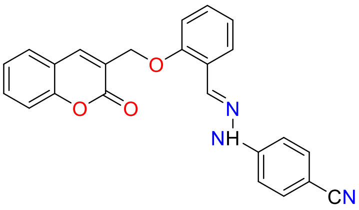

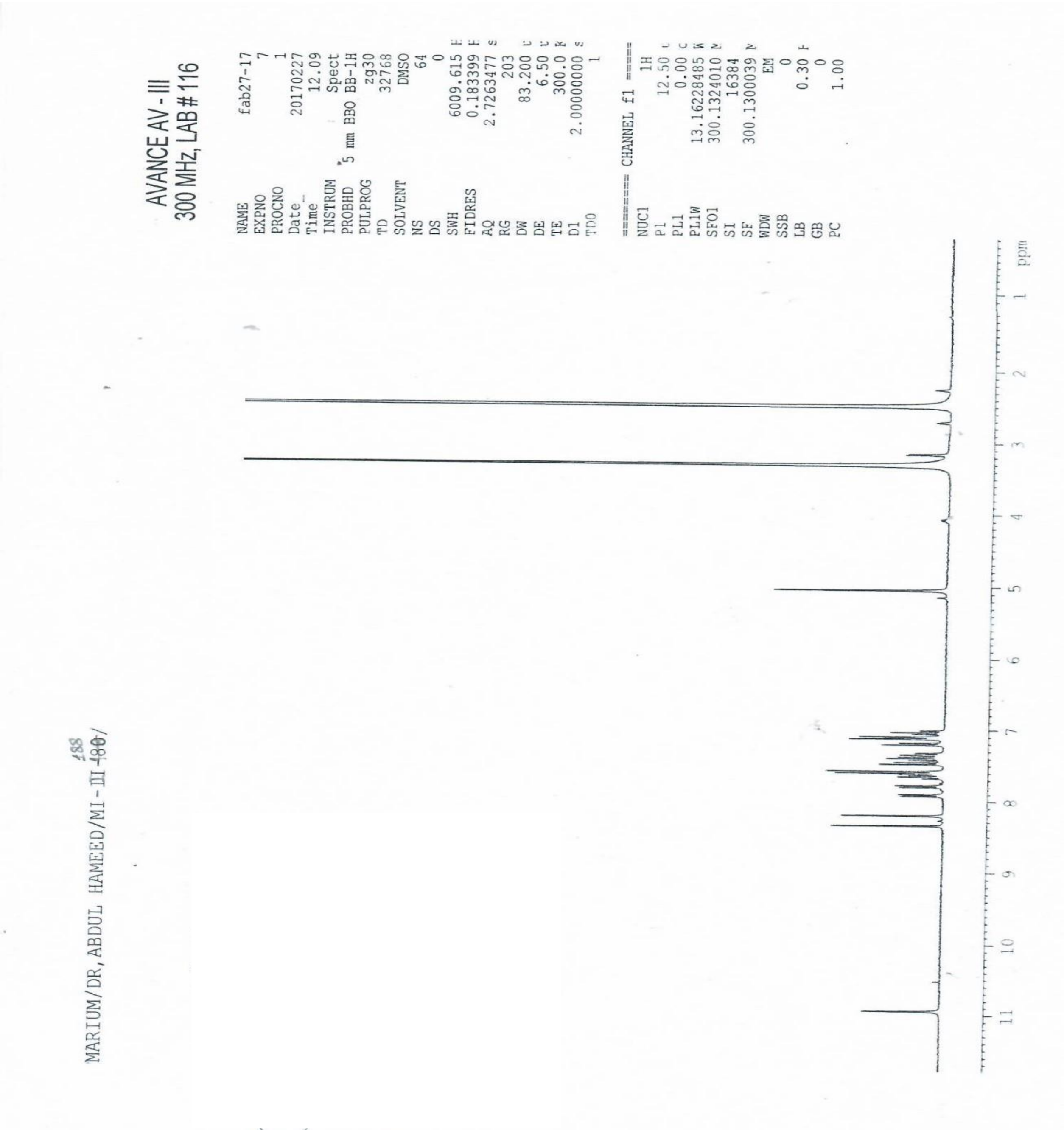

HEJ-ICCBS
12/2/2016 11:58:25 AM

MI-III-188
File: ~~MI-II-80~~
Sample: MARIUM /DR. HAMEED
Instrument: JEOL MS 600H-1

Date Run: 12-02-2016 (Time Run: 11:49:00)

Ionization mode: EI+

Scan: 52
Base: m/z 159; 28.4%FS TIC: 1679658

R.T.: 4.5

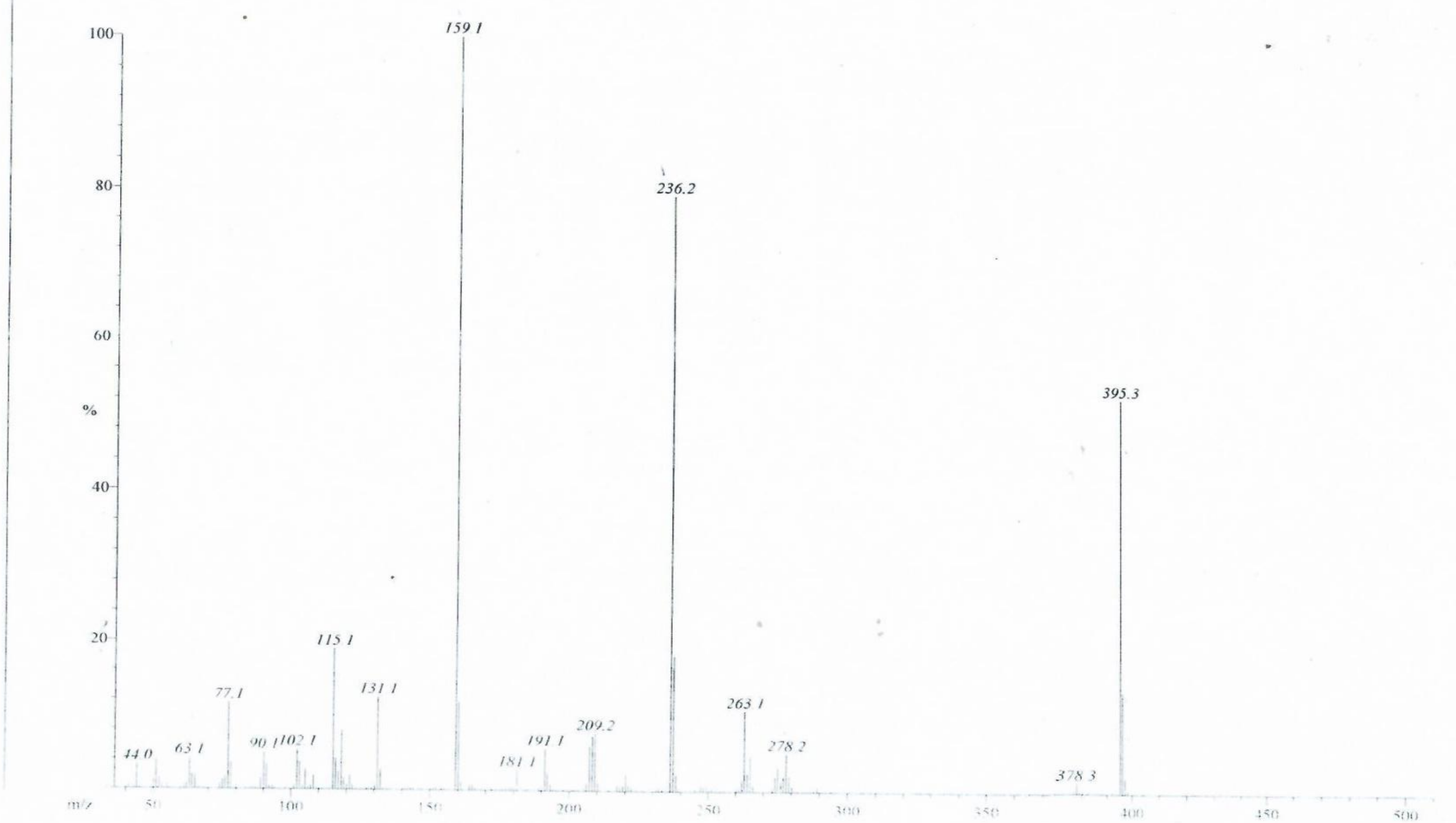

3-((2-((2-(2-Nitrophenyl)hydrazineylidene)methyl)phenoxy)methyl)-2H-chromen-2-one (17)

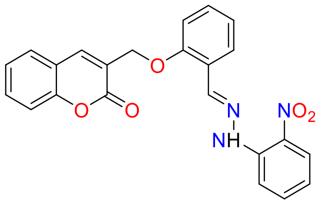

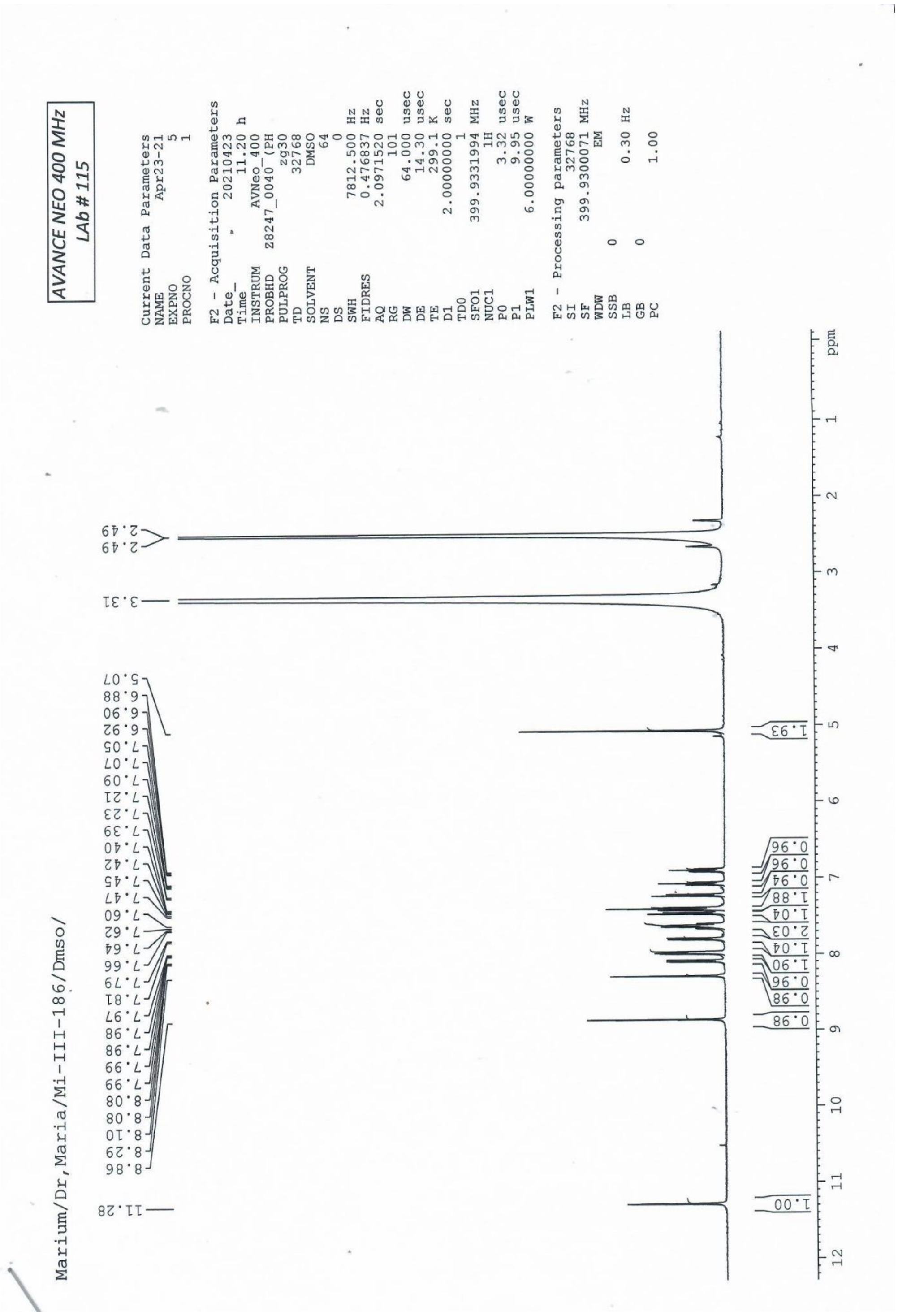

HEJ-ICCBS
4/24/2021 10:43:55 AM

File: MI-III-186 Date Run: 04-24-2021 (Time Run: 10:33:58)
Sample: MARIUM ISHTIAQ /DR. MARIA A. KHAN
Instrument: JEOL 600H-1 Run By: MASS LAB 104
Inlet: Direct Probe Ionization mode: EI+

Scan: 29
Base: m/z 159; 58.5%FS TIC: 4473856 **#Ions: 377**

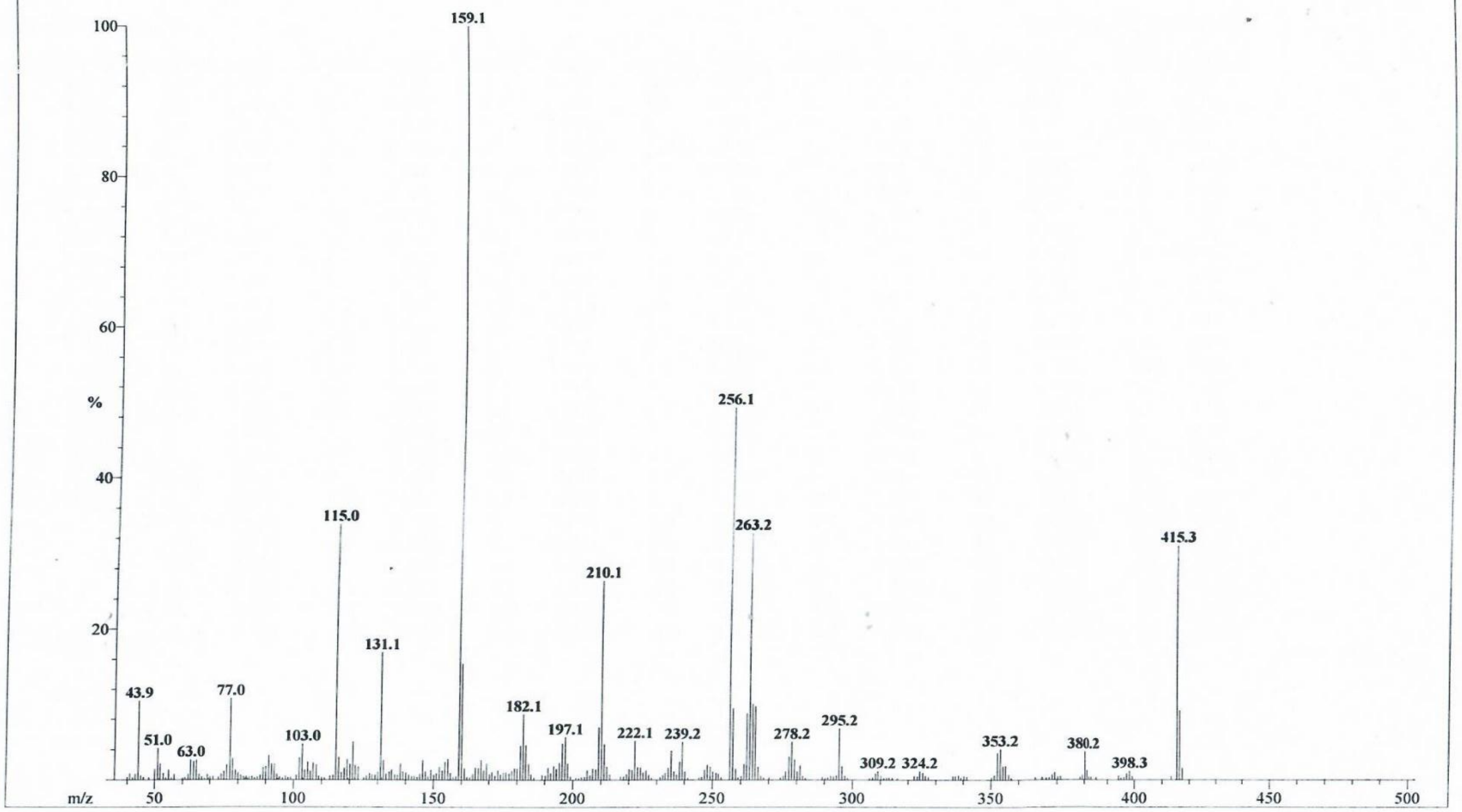

3-((2-((2-(3-Nitrophenyl)hydrazineylidene)methyl)phenoxy)methyl)-2H-chromen-2-one (18)

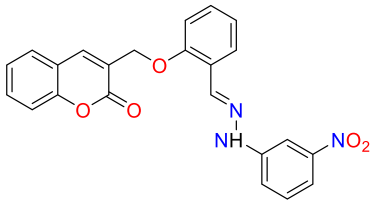

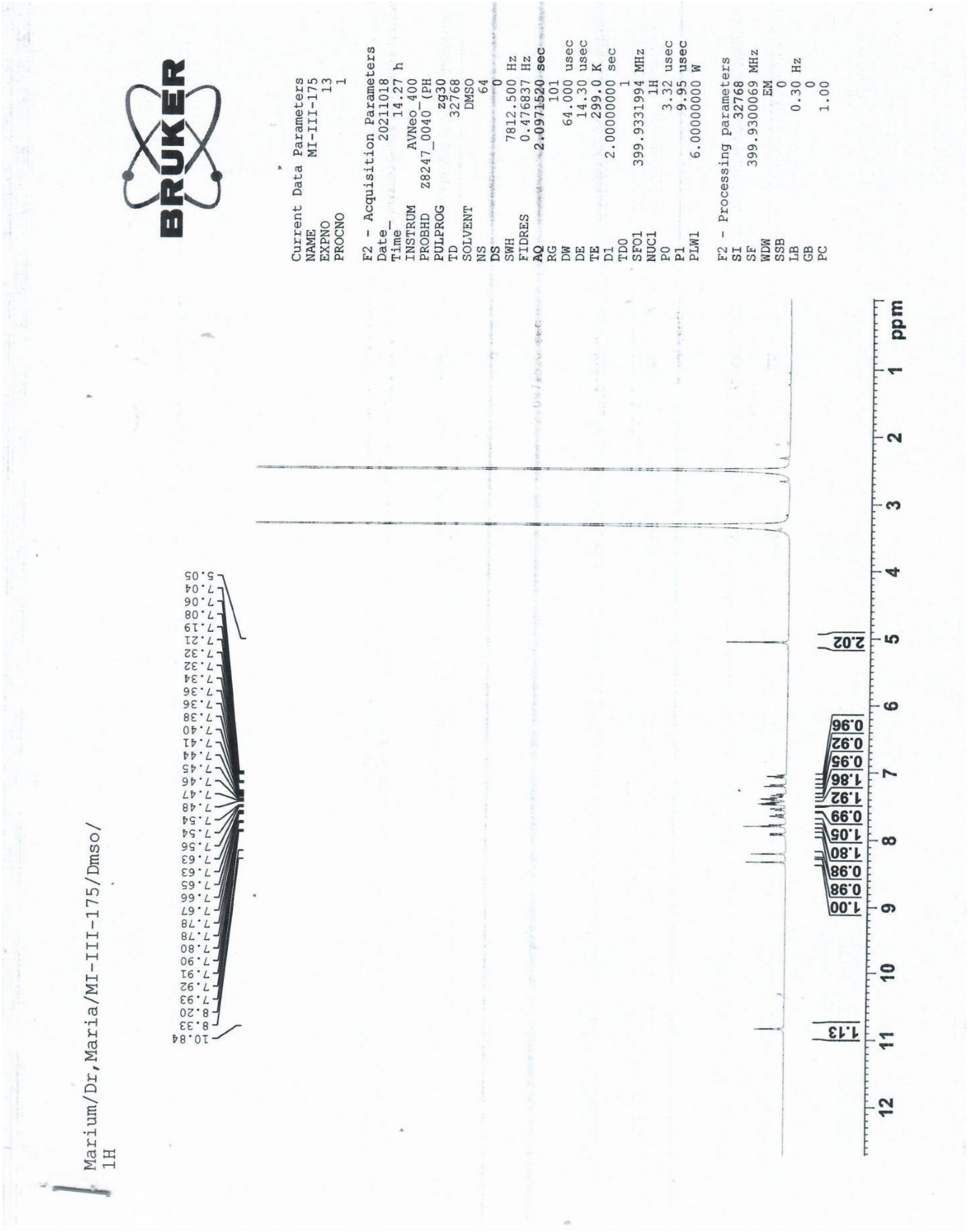

HEJ-ICCBS
10/18/2021 4:20:48 PM

File: MI-III-175
Sample: MARIUM ISHTIAQ / DR. MARIA
Instrument: JEOL 600H-1
Inlet: Direct Probe

Date Run: 10-18-2021 (Time Run: 16:11:52)

Ionization mode: EI+

Run By: MASS LAB 104

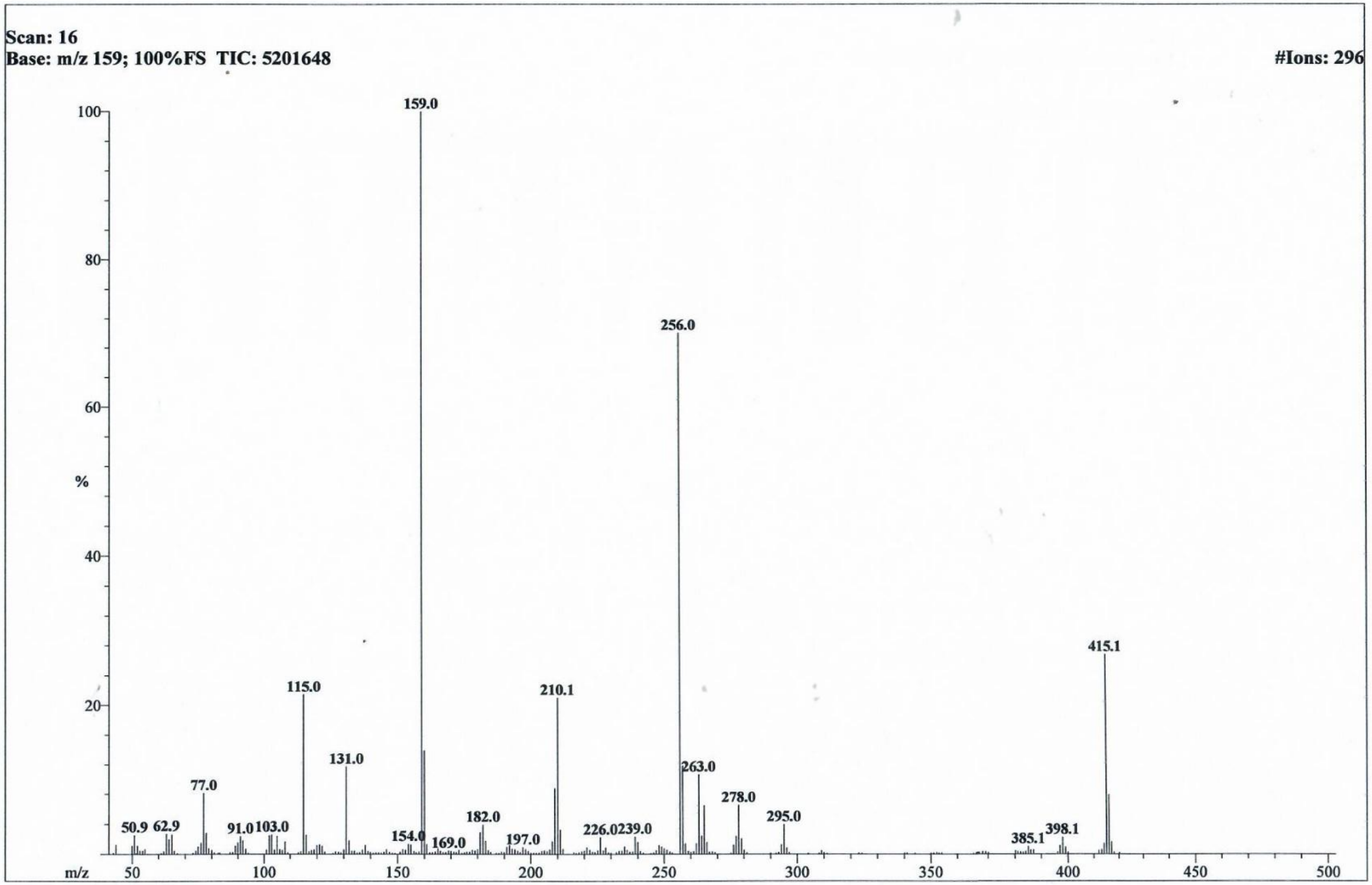

3-((2-((2-(2,3-Dimethylphenyl)hydrazineylidene)methyl)phenoxy)methyl)-2H-chromen-2-one (19)

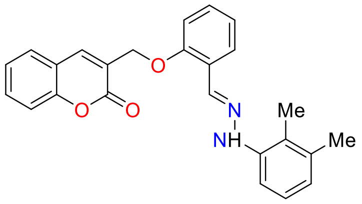

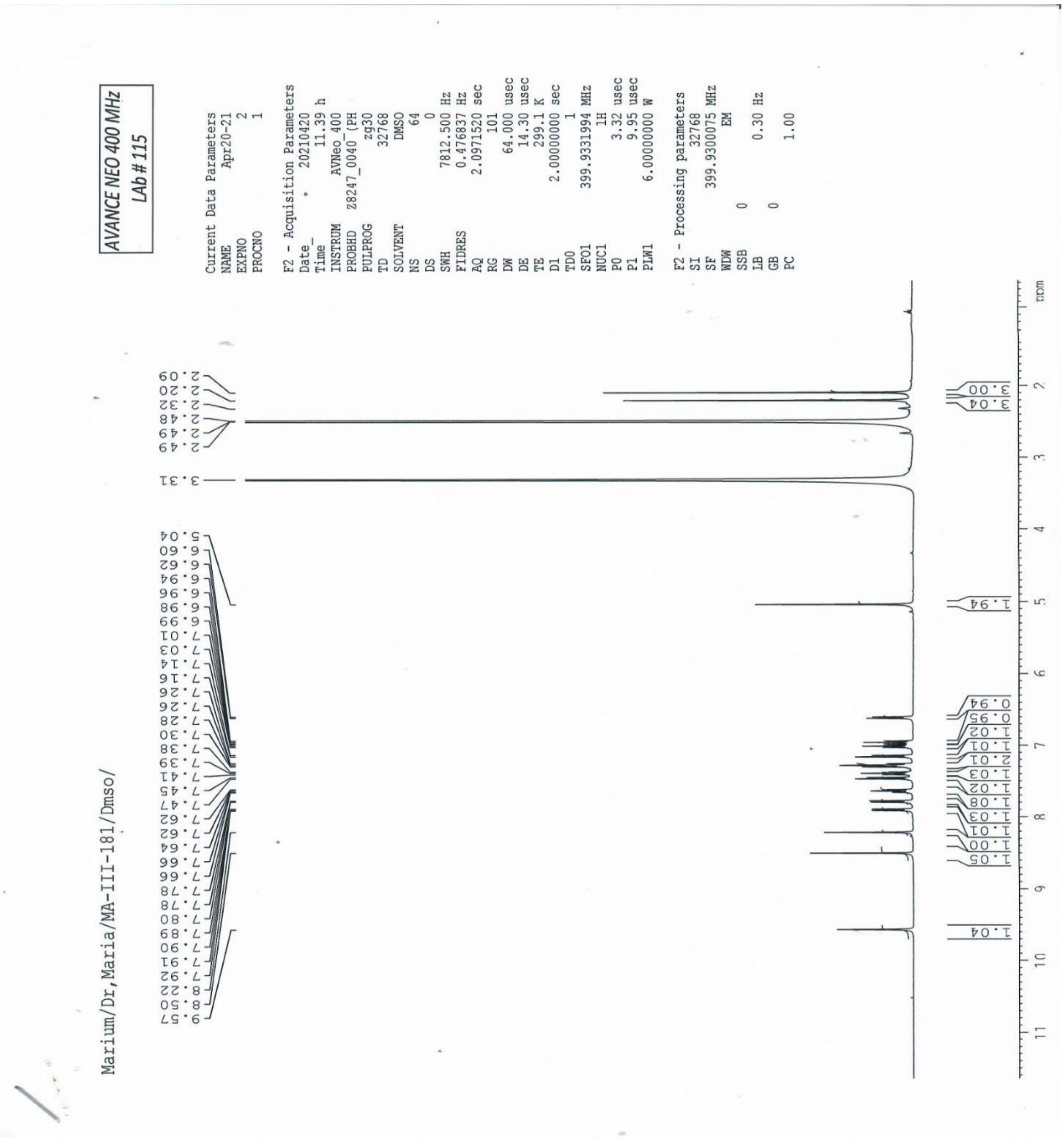

HEJ-ICCBS
4/20/2021 11:50:07 AM

File: MI-III-181
Sample: MARIUM /DR. MARIA
Instrument: JEOL 600H-1
Inlet: Direct Probe

Date Run: 04-20-2021 (Time Run: 11:43:50)

Ionization mode: EI+

Run By: MASS LAB 104

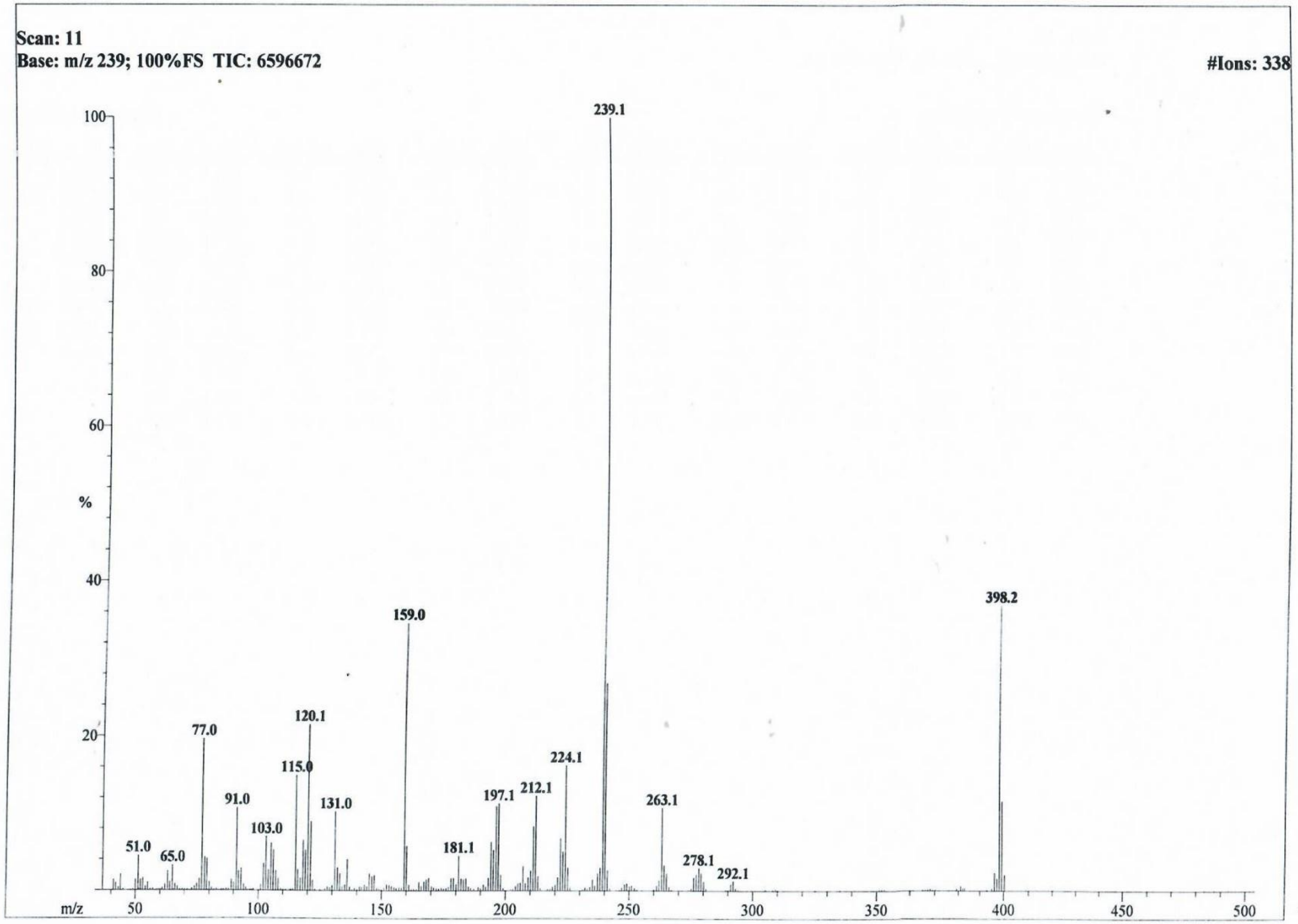

3-((2-((2-(2,4-Dimethylphenyl)hydrazineylidene)methyl)phenoxy)methyl)-2H-chromen-2-one (20)

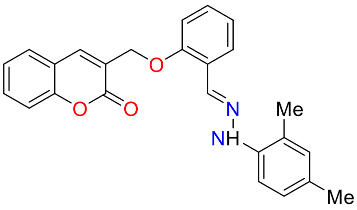

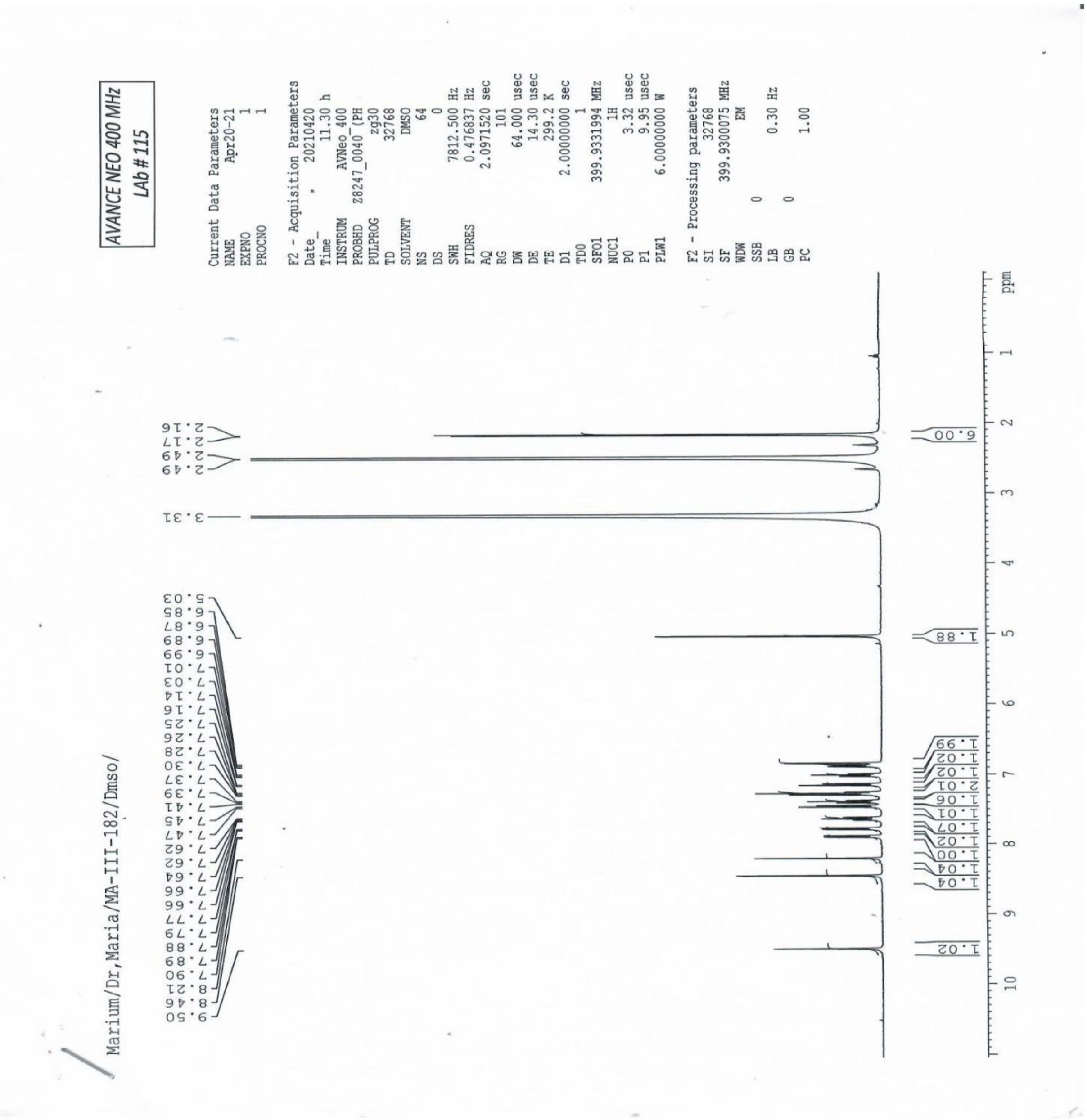

HEJ-ICCBS
4/20/2021 12:31:23 PM

File: MI-III-182
Sample: MARIUM /DR. MARIA
Instrument: JEOL 600H-1
Inlet: Direct Probe

Date Run: 04-20-2021 (Time Run: 12:24:15)

Ionization mode: EI+

Run By: MASS LAB 104

Scan: 19
Base: m/z 239; 100%FS TIC: 9328352

#Ions: 290

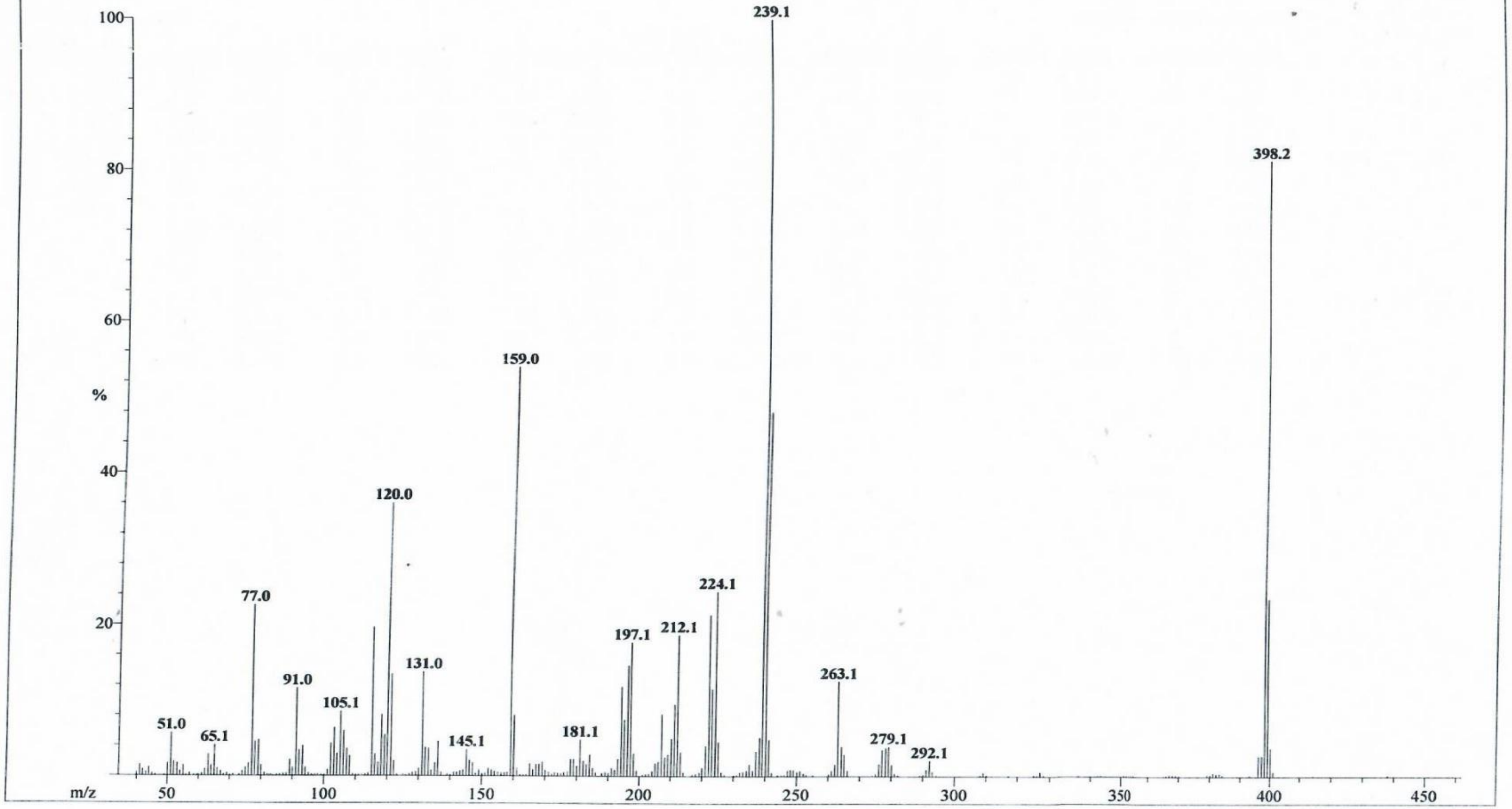

3-((2-((2-(2,6-Dichlorophenyl)hydrazineylidene)methyl)phenoxy)methyl)-2H-chromen-2-one (21)

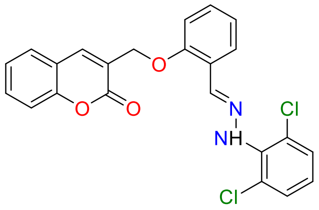

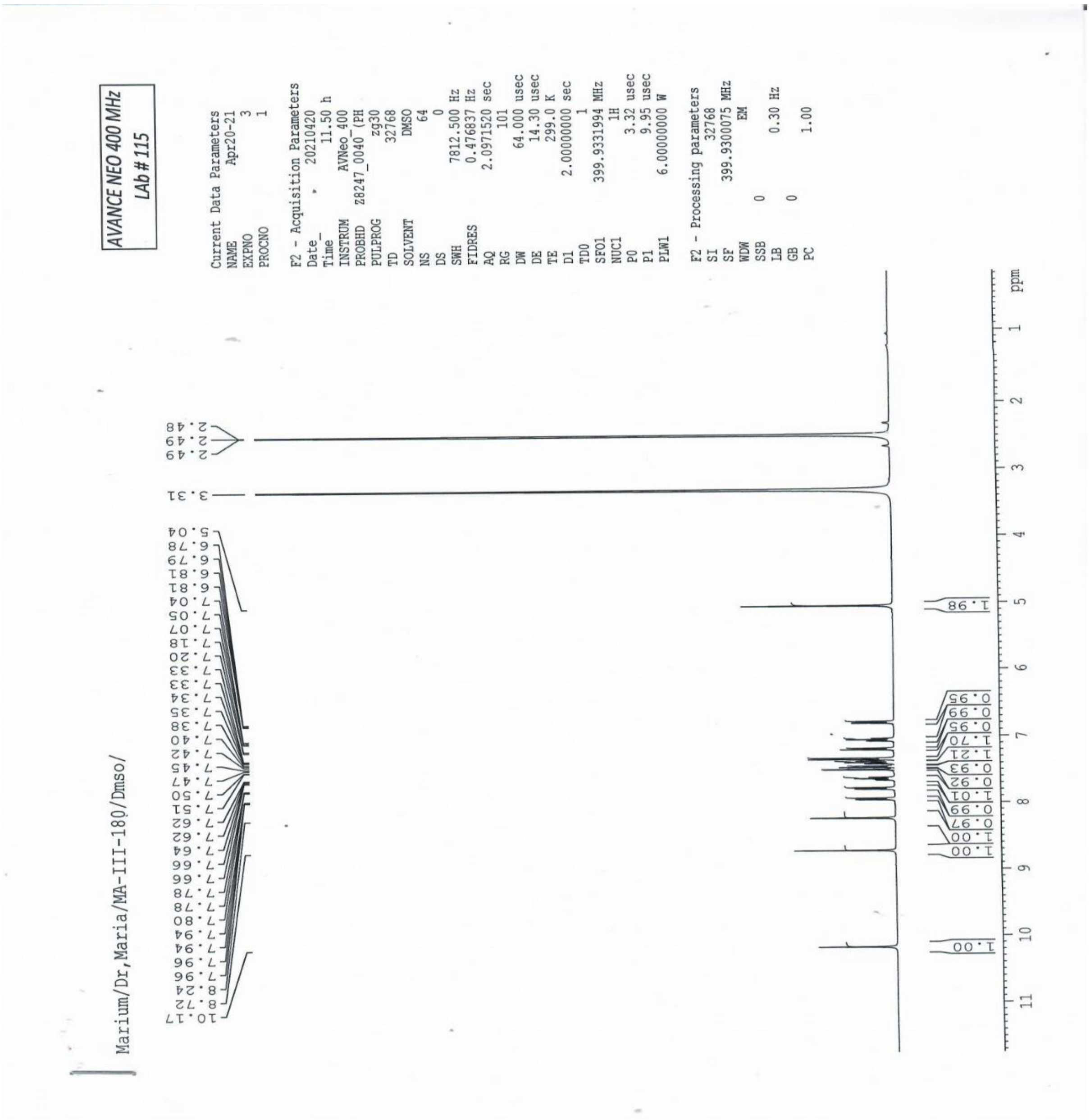

HEJ-ICCBS
4/20/2021 11:59:01 AM

File: MI-III-180
Sample: MARIUM /DR. MARIA
Instrument: JEOL 600H-1
Inlet: Direct Probe

Date Run: 04-20-2021 (Time Run: 11:52:42)

Ionization mode: EI+

Run By: MASS LAB 104

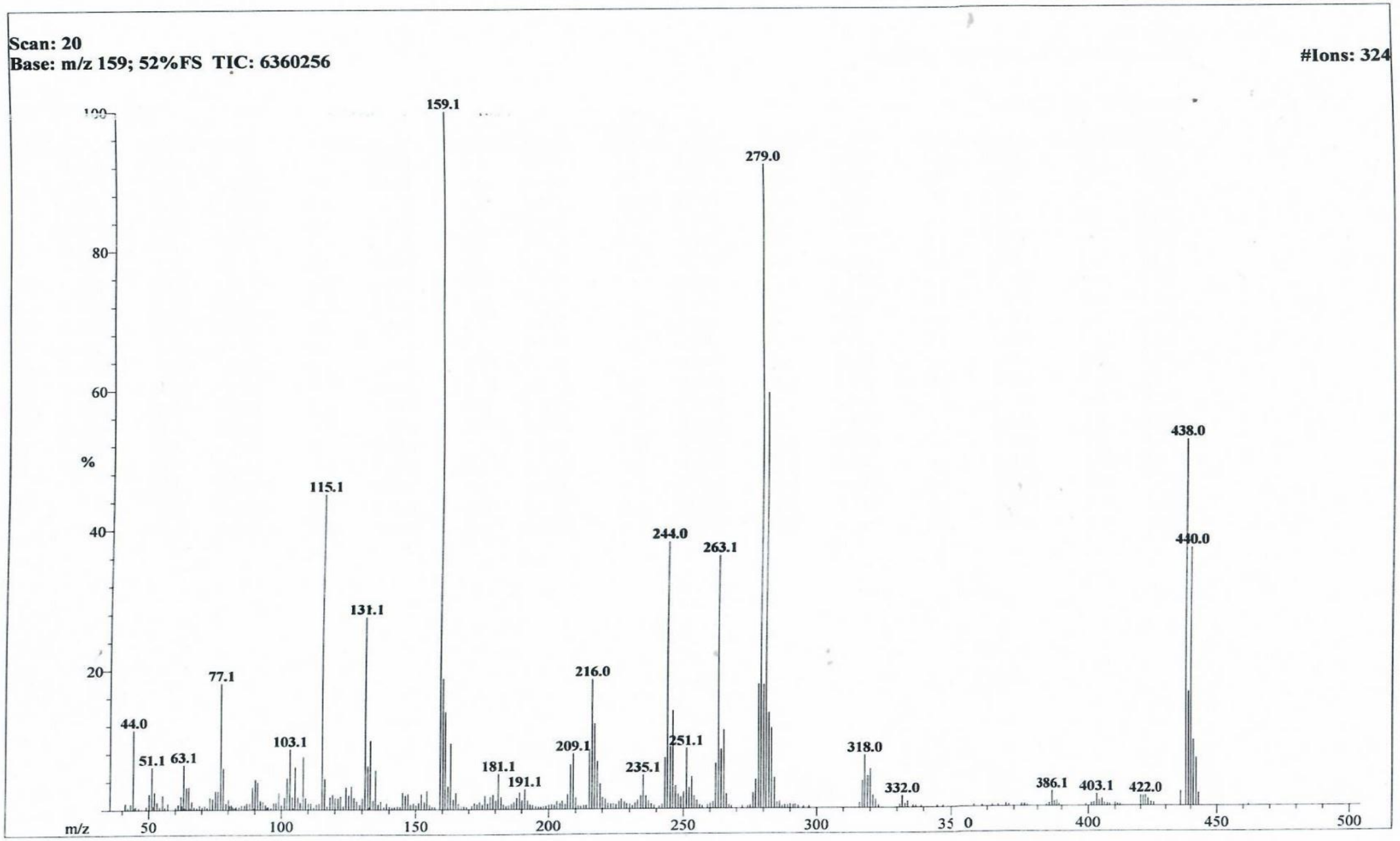

3-((2-((2-(2,4-Dinitrophenyl)hydrazineylidene)methyl)phenoxy)methyl)-2H-chromen-2-one (22)

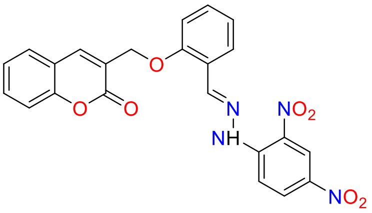

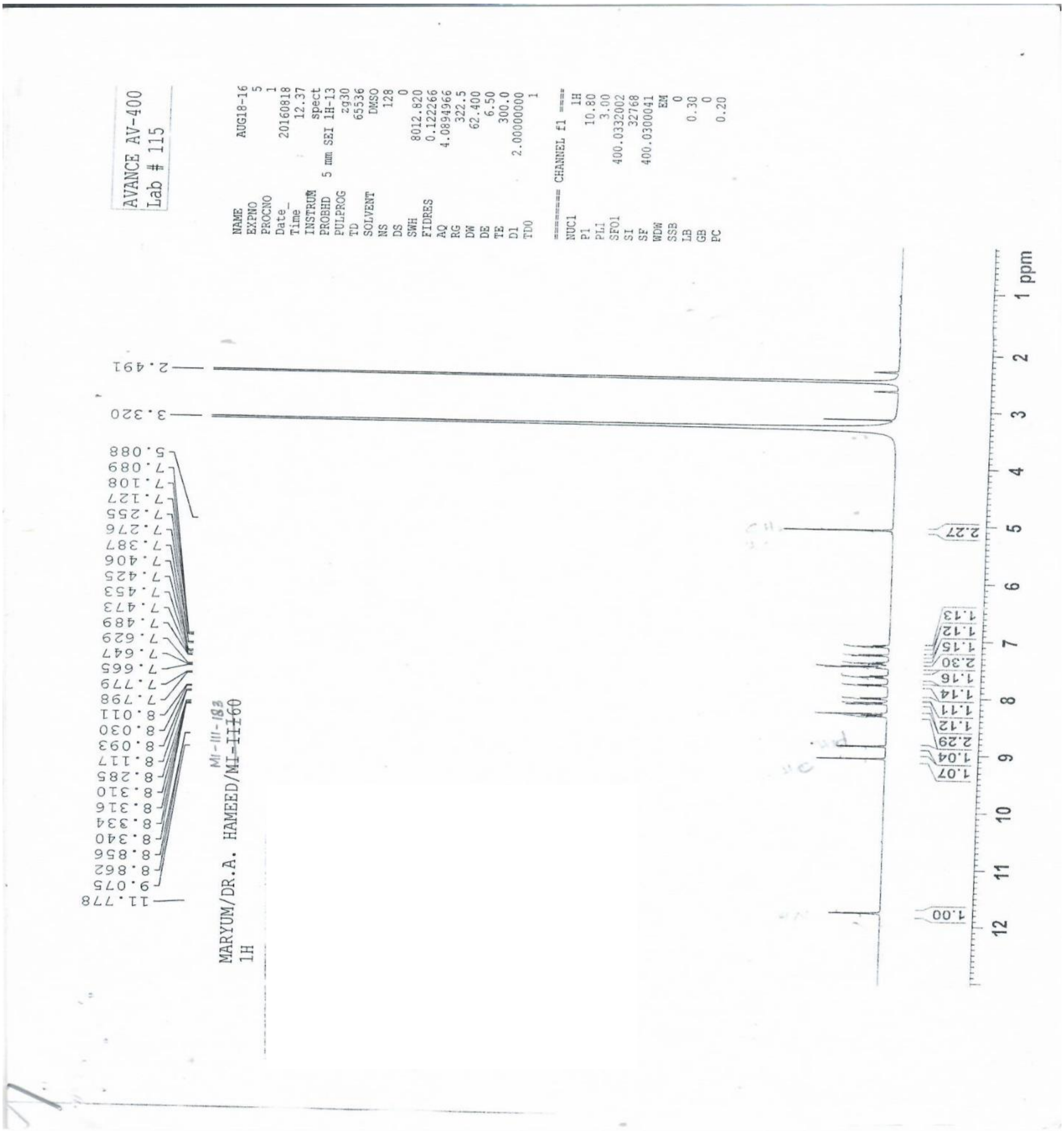

HEJ-ICCBS
4/23/2021 1:23:48 PM

File: MI-III-183 Date Run: 04-23-2021 (Time Run: 13:16:14)
Sample: MARIUM ISHTIAQ /DR. MARIA A. KHAN
Instrument: JEOL 600H-1 Run By: MASS LAB 104
Inlet: Direct Probe Ionization mode: EI+

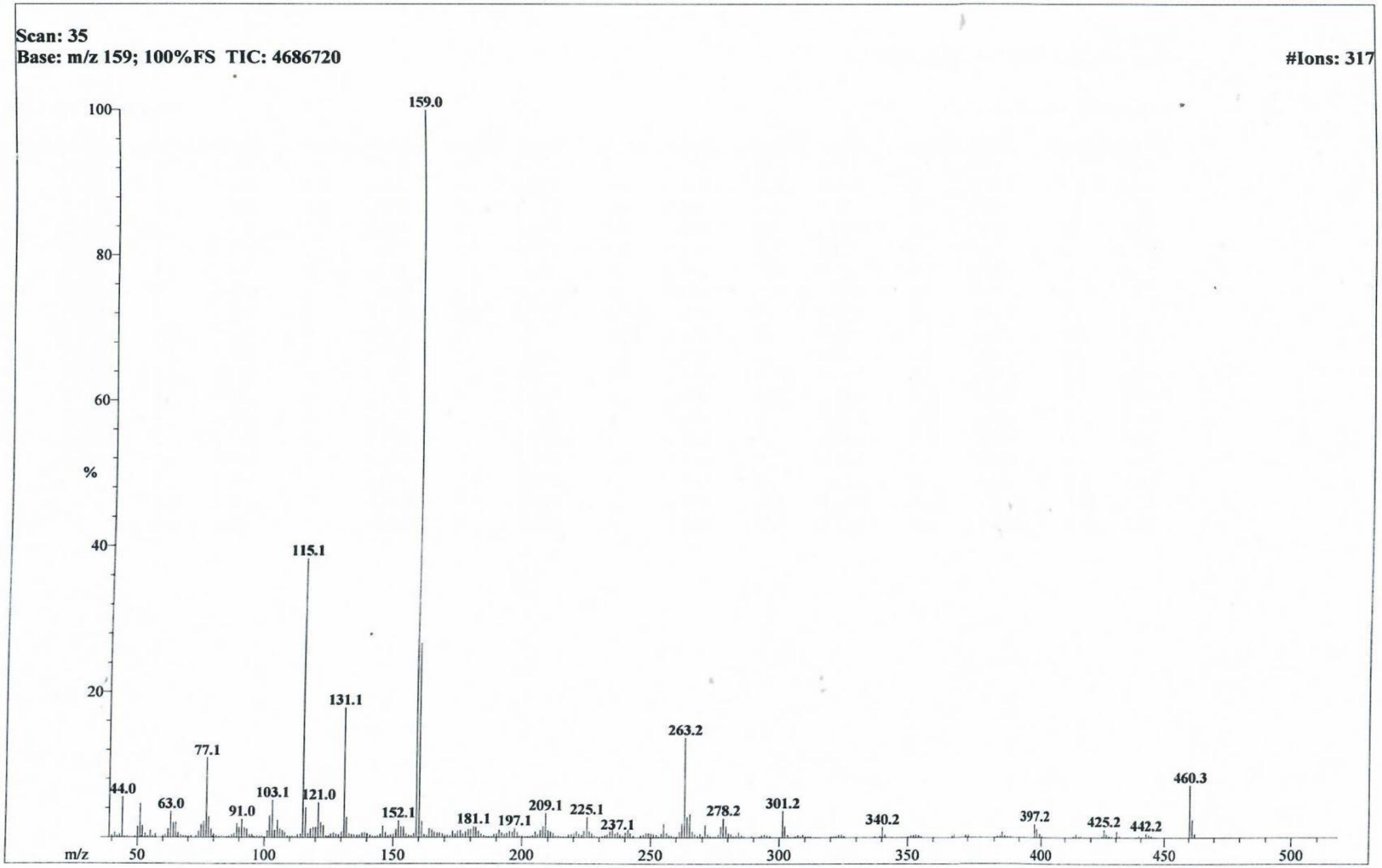

S. no.	Compound #	Structures	IC_{50} Graph for ALR2 Inhibition
1.	**5**	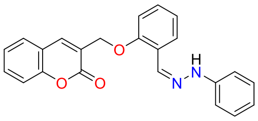<br>5	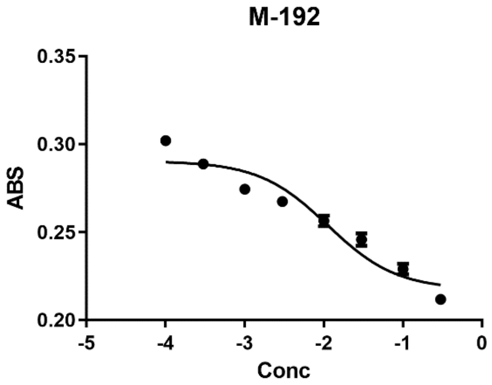
2.	**6**	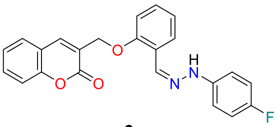<br>6	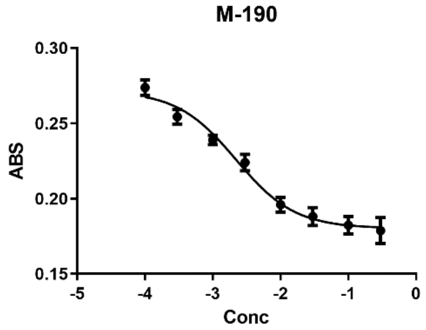
3.	**7**	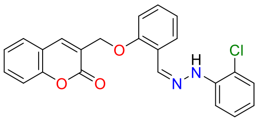<br>7	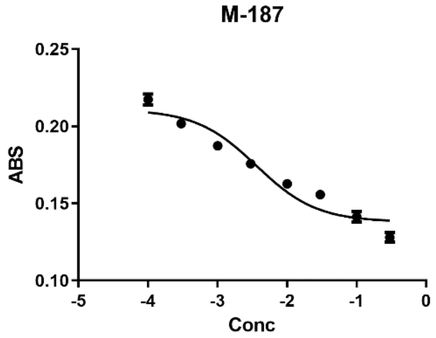
4.	**8**	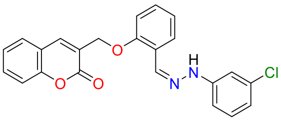<br>8	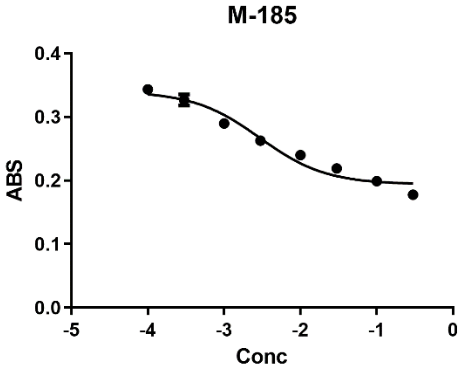

<table>
<tr><td>5.</td><td>9</td><td>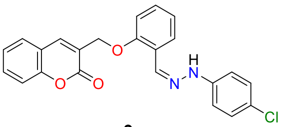
9</td><td>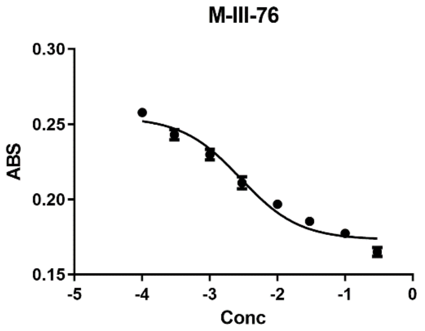</td></tr>
<tr><td>6.</td><td>12</td><td>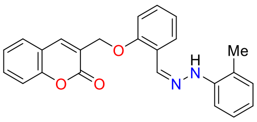
12</td><td>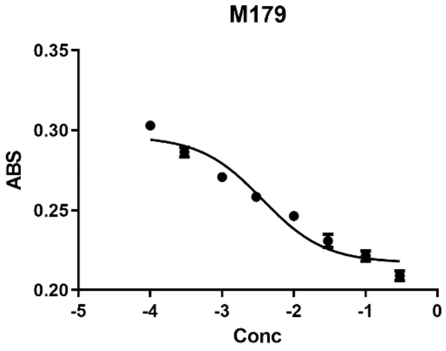</td></tr>
<tr><td>7.</td><td>13</td><td>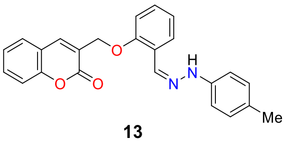
13</td><td>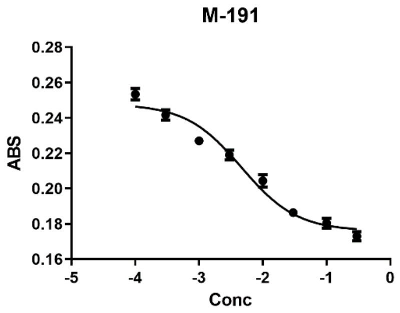</td></tr>
<tr><td>8.</td><td>14</td><td>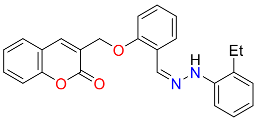
14</td><td>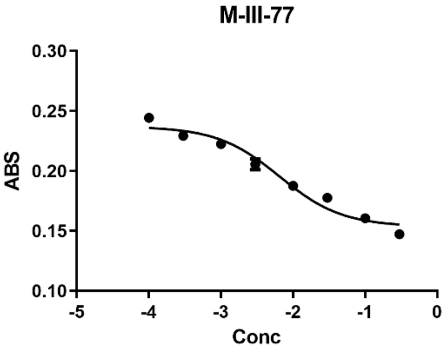</td></tr>
<tr><td>9.</td><td>15</td><td>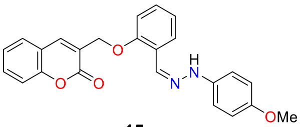
15</td><td>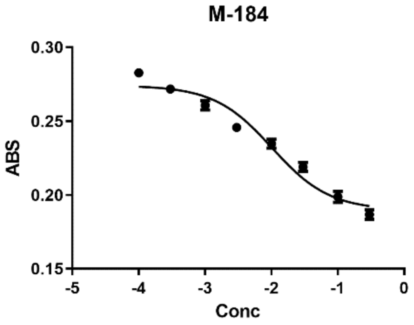</td></tr>
</table>

10.	**19**	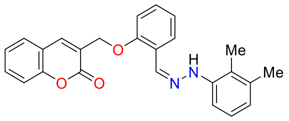 19	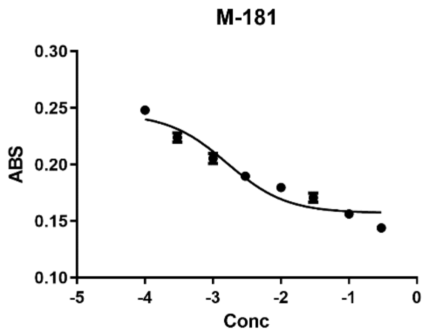
11.	**20**	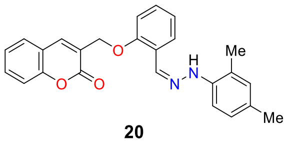 20	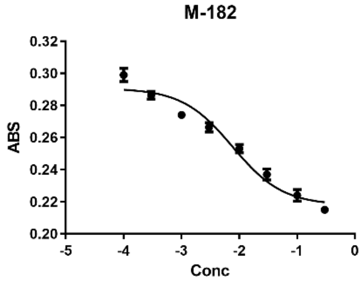
12.	**21**	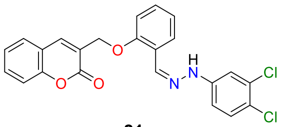 21	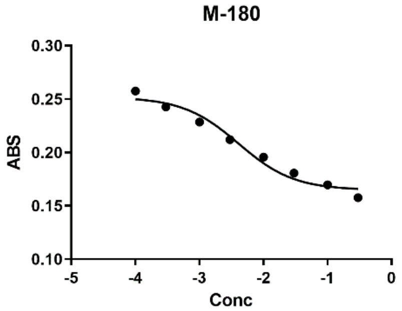

Table-S1: IC_{50} graphs of active compounds against ALR2 enzyme.

S. no.	Compound #	Structures	IC_{50} Graph for ALR1 Inhibition
1.	**7**	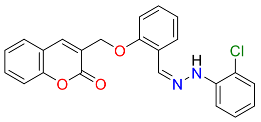 **7**	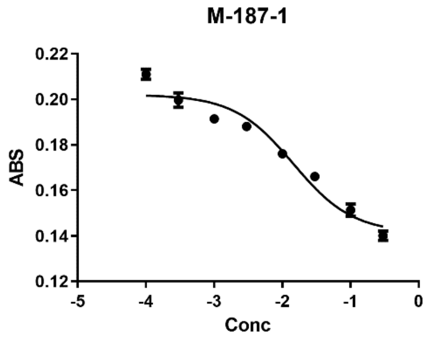
2.	**8**	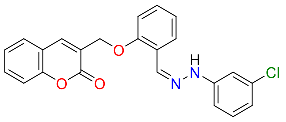 **8**	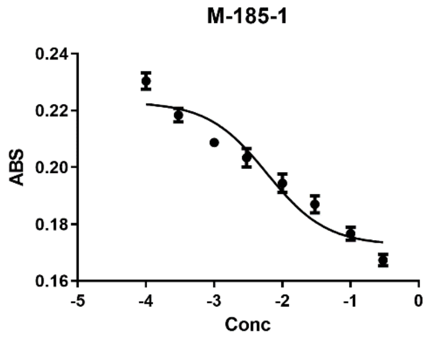
3.	**9**	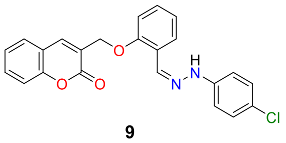 **9**	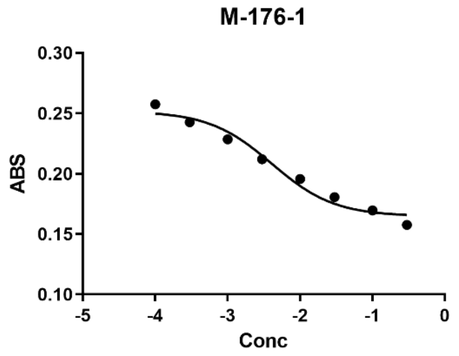
4.	**14**	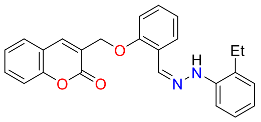 **14**	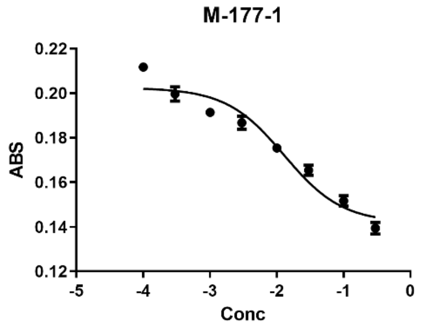

5.	**15**	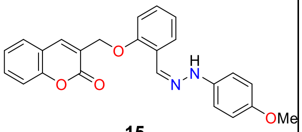<br>15	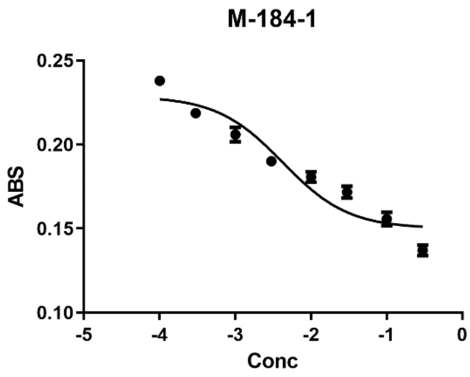
6.	**20**	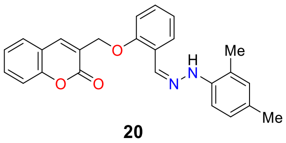<br>20	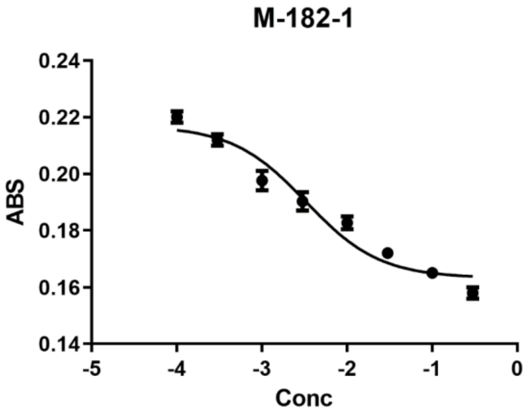
7.	**21**	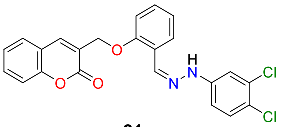<br>21	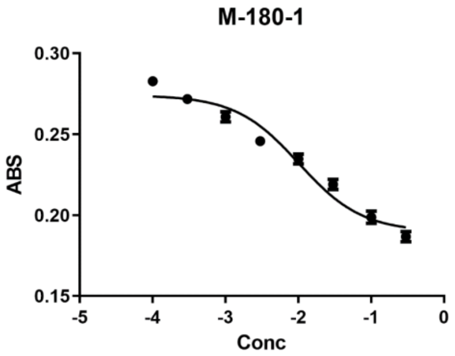

Table-S2: IC_{50} graphs of active compounds against ALR1 enzyme.

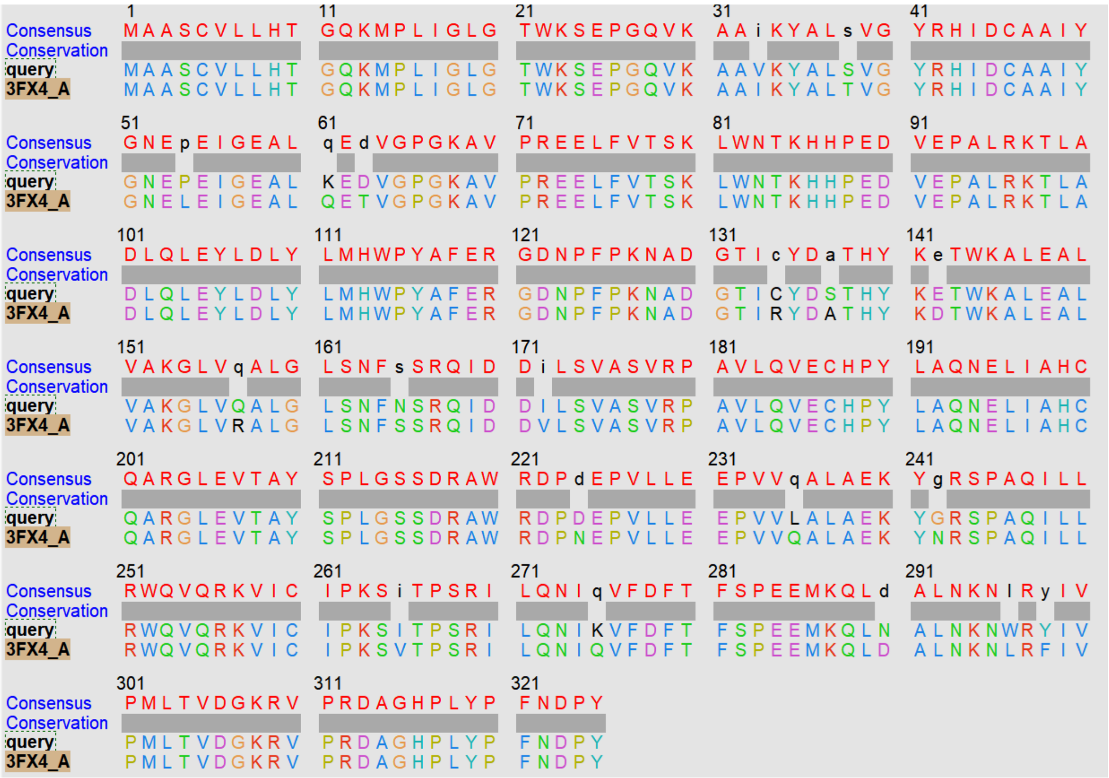

Fig. S1. Sequence alignment of target protein (hALR1) and template protein (porcine alcohol dehydrogenase, PDB id:3fx4a).

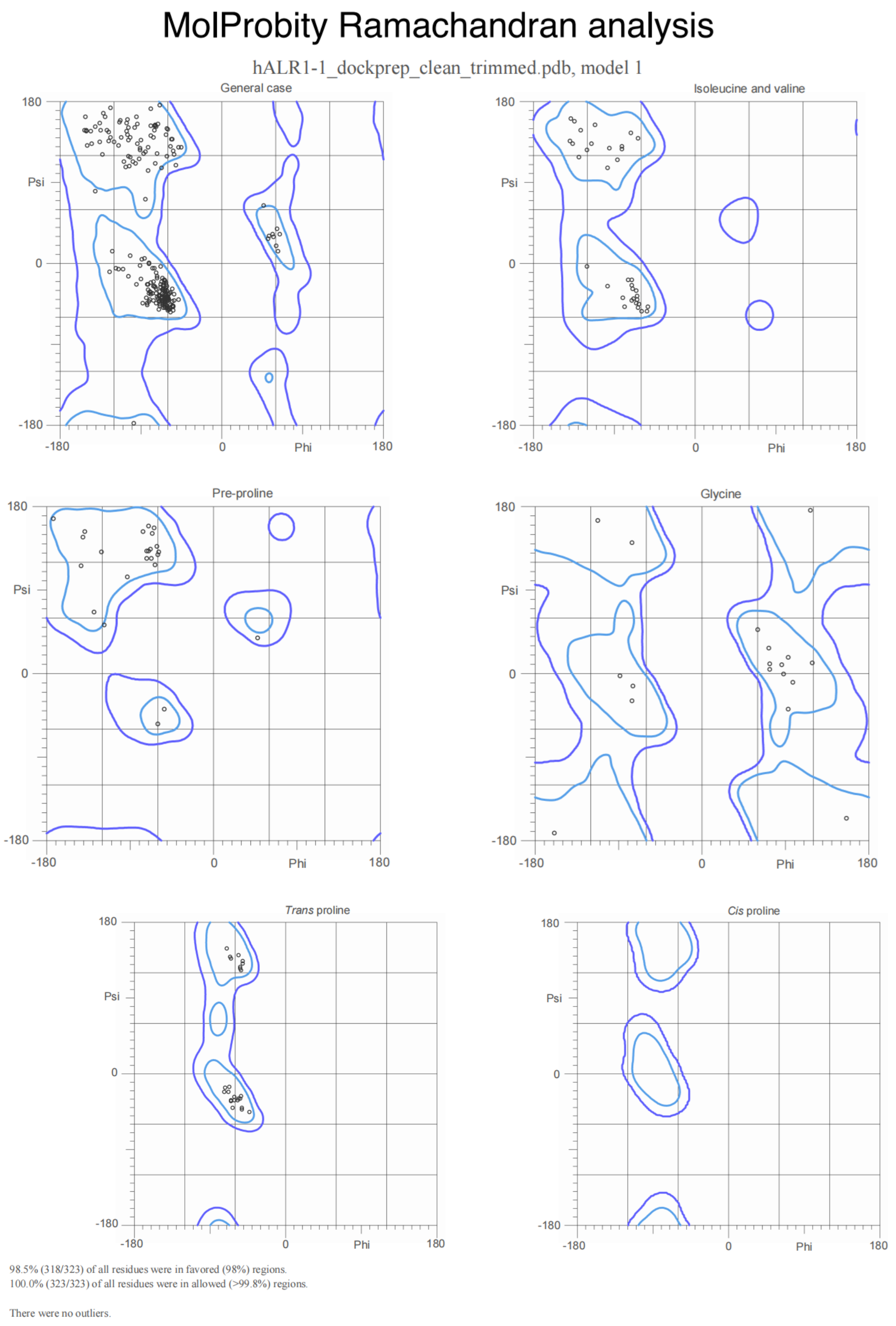

Fig. S2. Ramachadran plot of homology model of hALR1.

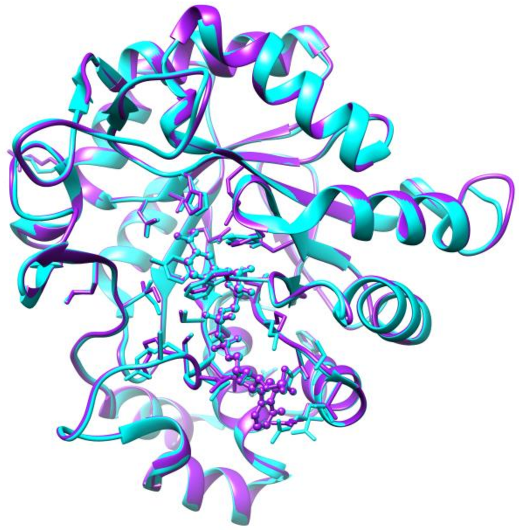

Fig. S3. Overlap of target protein (hALR1, *purple*) with template protein (porcine, alcohol dehydrogenase, *cyan*). NADPH co-factor is shown in balls and sticks model.

S. no.	Structures	Docking Score against ALR2 enzyme
**1.**	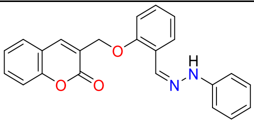 **5**	-23.7
**2.**	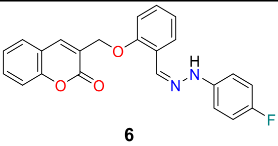 **6**	-26
**3.**	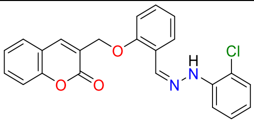 **7**	-29
**4.**	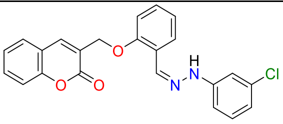 **8**	-26.5
**5.**	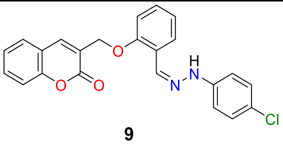 **9**	-20.6
**6.**	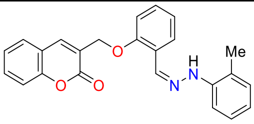 **12**	-26.4
**7.**	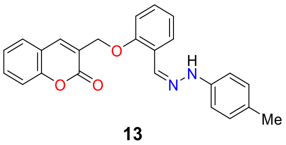 **13**	-21.3
**8.**	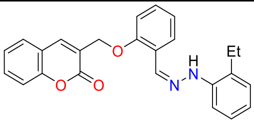 **14**	-26.6
**9.**	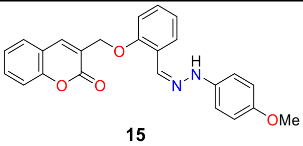 **15**	-24.1
**10.**	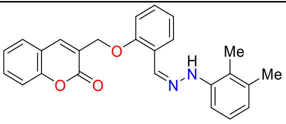 **19**	-27
**11.**	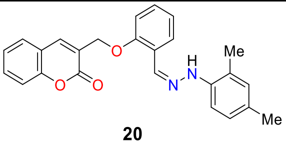 **20**	-24.6

**12.**	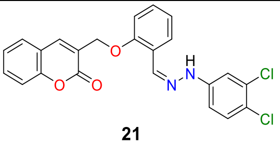<br>**21**	-24.9

Table-S3: Docking scores of active compounds against ALR2 enzyme.

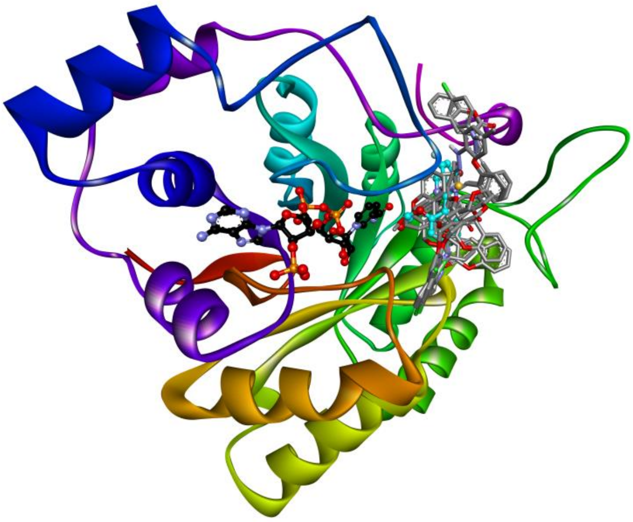

Fig. S4. Overlap of ALR2 inhibitors **5-9**, **12-15**, **19-21** (*grey*), all compounds bind with similar conformations in the same area of the binding site as the co-crystallized inhibitor (*cyan*).

S. no.	Structures	Docking Score against ALR1 enzyme
1.	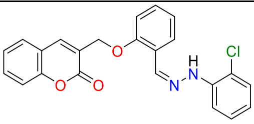 **7**	-21
2.	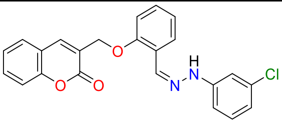 **8**	-24.4
3.	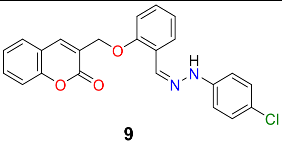 **9**	-23.3
4.	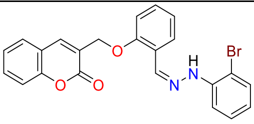 **10**	-23.6
5.	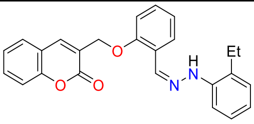 **14**	-23.5
6.	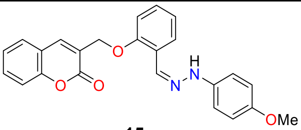 **15**	-23.3
7.	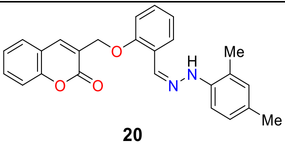 **20**	-23
8.	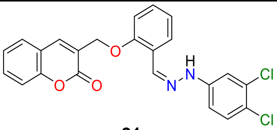 **21**	-21

Table-S4: Docking scores of active compounds against ALR1 enzyme.

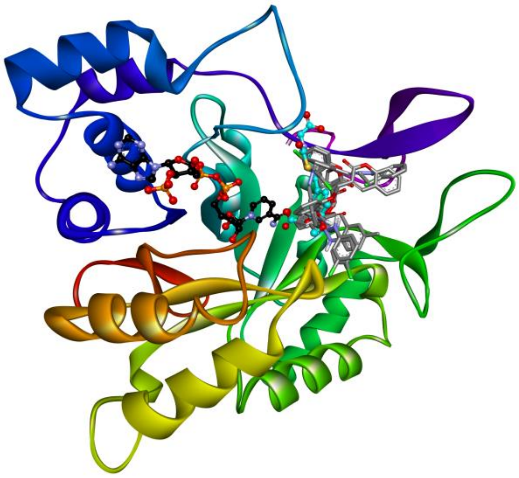

Fig. S5. Overlap of ALR1 inhibitors 7**-10, 14-15, 20** (*grey*), all compounds bind with similar conformations in the same area of the binding site as the co-crystallized inhibitor (*cyan*).
